# Analytical Review on Normal Brain Aging, Alzheimer’s Disease, and Stage 4S Neuroblastoma: Novel Insights Into Neuroprotection, Neurodegeneration, and Spontaneous Regression

**DOI:** 10.7759/cureus.93818

**Published:** 2025-10-04

**Authors:** Nevim Aygun

**Affiliations:** 1 Department of Medical Biochemistry, Faculty of Medicine, Ege University, Izmir, TUR

**Keywords:** alzheimer’s disease, neurodegeneration, neurodegenerative diseases, neuroprotection, normal brain aging, spontaneous regression, stage 4s neuroblastoma

## Abstract

The normal aging brain does not show massive neuron loss. However, neurodegenerative diseases are characterized by the progressive loss of neurons that differentiate from neuroblasts arising in the neural tube. Oxidative stress induces protein misfolding and aggregation that generates endoplasmic reticulum (ER) stress, leading to the activation of the unfolded protein response (UPR). Oxidative stress can also impair autophagy and mitophagy. Prolonged ER stress and impaired autophagy or mitophagy together can promote neuronal death. Neuroblastoma is an embryonal tumor developing from sympathetic neuroblasts. Stage 4S neuroblastoma is frequently associated with spontaneous regression. Autophagy decreases in stage 4S neuroblastoma, whereas apoptosis increases. The present review focuses on the molecular and cellular fundamentals of normal brain aging, neurodegenerative diseases, and neuroblastoma with spontaneous regression. Differential gene expressions and associated potential mechanisms of the normal aging brain, Alzheimer’s disease (AD) brain, and stage 4S neuroblastoma are investigated by analyzing the Gene Expression Omnibus (GEO) datasets and publications. In conclusion, there are gender- and region-specific differential expressions and connected differential molecular mechanisms in the normal aging brain and AD brain. PRH1 and *MIR181A1HG* are likely to play neuroprotective roles in certain brain regions of elderly females. The loss of myelinating oligodendrocytes in the entorhinal cortex (EC) and repressed adult hippocampal neurogenesis can contribute to cognitive impairment in AD. Neurodegeneration of mature neurons and spontaneous regression of immature sympathetic neuroblast-derived neuroblastomas may occur via similar cellular behavior of mature and immature nerve cells. Impaired autophagy and apoptosis can play crucial roles in both synaptic dysfunction and neuron death in AD and the spontaneous regression of stage 4S neuroblastoma. Finally, the present analytical review provides novel insights into apoptosis, neuronal differentiation, immune response, impaired autophagy, and impaired double-strand break (DSB) repair, considered potential mechanisms underlying spontaneous regression in stage 4S neuroblastoma.

## Introduction and background

The aging brain has many indicators, including dysregulation of energy metabolism, mitochondrial dysfunction, aberrant neuronal activity, oxidative damage, adaptive stress response failure, impairment of autophagy-lysosome and proteasome pathways, dysregulation of neuronal calcium homeostasis, glial cell activation, neuroinflammation, stem cell exhaustion, and inefficient DNA repair [1]. The aging process may increase the risk of neurodegeneration in the brain [2]. Neurodegeneration in neurodegenerative diseases leads to progressive loss of structure or function of mature neurons, which differentiate from neuroblasts arising from neural tube-derived neuroepithelial stem cells, in the central nervous system (CNS) [3,4]. Neurodegeneration can also be associated with the loss of myelin [5].

Neurodegenerative processes share common hallmarks, including disrupted proteostasis, metabolic dysfunction, oxidative stress, endoplasmic reticulum (ER) stress, and neuroimmune system alterations [6]. The proteostasis network, consisting of about 2,000 proteins, orchestrates the maintenance of protein homeostasis. It regulates all proteomic processes, including protein synthesis, folding, conformational stability, trafficking, aggregation/disaggregation, and degradation. Molecular chaperones, the ubiquitin-proteasome system, the autophagosomal-lysosomal system, and their regulators are basic components of the proteostasis network. External or endogenous factors such as heavy metals, heat shock, reactive oxygen species, mutations, defective mRNA, and translation errors may cause disrupted proteostasis by impairing homeostatic pathways and lead to misfolding and aggregation of specific proteins in older people. The accumulation of these protein aggregates damages postmitotic neurons in age-dependent neurodegenerative disorders [7]. The neurons are the most vulnerable cell type to protein aggregation in the brain. The protein homeostasis response against protein aggregation is weaker in postmitotic neurons compared to other non-neuronal cell types in the brain. In addition, there is a weaker response of the protein homeostasis system controlling the aggregation of metastable proteins, particularly in the oxidative phosphorylation pathway in the brain tissues, compared to other body tissues [8]. Neurons demonstrate high metabolic activity primarily dependent on the oxidative phosphorylation pathway in mitochondria, making them more vulnerable to oxidative stress. High metabolic activity elevates reactive oxygen species, forming an increased risk for DNA damage [9]. Oxidative stress triggers protein misfolding and aggregation, resulting in irreversible neuron damage and later cell death. There are oxidative stress-induced impairments in the ubiquitin-proteasome system, chaperone-mediated autophagy (CMA), macroautophagy, and mitophagy [10]. 

Misfolded protein aggregates induce ER stress, activating the unfolded protein response (UPR). Prolonged ER stress causes neuronal cell death [11]. However, chronic ER stress can result in the accumulation of protein aggregation as well. For example, disulfide-cross-linked wild-type superoxide dismutase 1 (SOD1) aggregates that are observed in amyotrophic lateral sclerosis (ALS) have been reported to accumulate in microsomes of the spinal cord during the aging of mice exposed to chronic ER stress. Although this aggregation does not lead to significant neuronal loss, it is associated with the activation of astrocytes under ER stress [12]. Another study exhibits that ER stress leads to cytosolic protein aggregation rather than proteins of the ER or secretory system due to the insufficiency of the chaperones in yeast. It triggers prion formation as well [13].

The vessel wall injury generated by endothelial and pericyte deterioration abrogates the integrity of the blood-brain barrier (BBB), resulting in neurodegeneration. Because the impairment of the BBB allows blood-borne neurotoxicity and neuroinflammation maintained by the central and peripheral immune systems. Peripheral immunity triggers innate and adaptive responses by neutrophils, macrophages, and T and B lymphocytes. Microglia and astrocytes, which are players of the central immune system, activate due to neurotoxic debris and decreased blood flow. Central immune cells secrete neurotoxic cytokines and chemokines, inducing neuroinflammation in the CNS. Upon BBB breakdown, these immune pathways mediate neuronal injury and synaptic dysfunction, resulting in neurodegeneration [14]. On the other hand, artificial amyloid-like β-sheet proteins produce a fibrillar aggregate structure. This fibrillation leads to the neurotoxic effect via the gain-of-function of protein aggregation in neurons. This abnormal structure creates lysosomal defects by affecting the late stages of autophagy [15]. Amyloid-β (Aβ) proteins can also be associated with the loss or degeneration of pericytes, resulting in disrupted BBB, followed by neurodegeneration [16].

Metabolic dysfunction is implicated in reduced glucose utilization, mitochondrial dysfunction, deregulated calcium signaling, impaired branched-chain amino acid metabolism, and dysregulated lipid metabolism in neurodegenerative diseases [17,18].

SH-SY5Y neuroblastoma cell line has been used as an *in vitro* model of many neurodegenerative disorders, including Alzheimer’s disease (AD), Parkinson’s disease (PD), multiple sclerosis (MS), ALS, Huntington’s disease (HD), and spinal muscular atrophy (SMA) [19-23]. Neuron-like cells can be derived from undifferentiated SH-SY5Y cells using various treatments, mostly retinoic acid and brain-derived neurotrophic factor [24].

Postmitotic neurons that are terminally differentiated are considered incapable of undergoing cell division. They usually survive under normal conditions throughout the lifespan of humans. Because normal aging does not cause massive neuronal loss. Adult neurogenesis, a process promoting the differentiation of neural stem cell-derived neuroblasts into mature neurons, can compensate for neuronal loss as well. However, neurodegenerative diseases show extensive neuron loss along with defective neurogenesis. Primary neurons re-enter the cell cycle during the developmental post-synaptogenesis stage. However, they mostly undergo cell death instead of cell division when they re-enter the cell cycle [25-29].

Tau, Aβ, the scrapie isoform of the prion protein (PrP^Sc^), α-synuclein (α-Syn), huntingtin (HTT), neurofilament, SOD1, TAR DNA binding protein 43 (TDP-43), and ataxins 1-3 (ATXN1-3) aggregates affect the pathogenesis of AD, progressive supranuclear palsy (PSP), prion diseases, PD, HD, ALS, frontotemporal dementia (FTD), and spinocerebellar ataxia (SCA) that are neurodegenerative diseases [30,31]. As a consequence of disrupted protein homeostasis, the toxic structure of misfolded protein aggregates plays an important role in neuronal cell death pathways, leading to neurodegeneration [32]. Evidence indicates that potential mechanisms of neuronal loss in neurodegenerative diseases include apoptosis, necrosis, necroptosis, ferroptosis, pyroptosis, and autophagic cell death [33].

Neuroblastoma is an embryonal tumor that develops from sympathetic neuroblasts arising from neural crest-derived progenitor cells of the peripheral sympathetic nervous system [34,35]. Heterogeneity reflecting clinical and genomic complexity is the most important hallmark of neuroblastoma [36]. Different clinicopathological, biological, biochemical, and genetic factors play a crucial role in determining the course of heterogeneous phenotypes in neuroblastoma. Molecular characteristics such as *MYCN* proto-oncogene, BHLH transcription factor (*MYCN*) amplification, 1p36 deletion, 11q loss, 17q gain, DNA ploidy level, the expression level of tyrosine kinase receptor A (*TRKA*)/neurotrophic receptor tyrosine kinase 1 (*NTRK1*), tyrosine kinase receptor B (*TRKB*)/neurotrophic receptor tyrosine kinase 2 (*NTRK2*), and tyrosine kinase receptor C (*TRKC*)/neurotrophic receptor tyrosine kinase 3 (*NTRK3*) receptors, and lactate dehydrogenase (LDH) level, as well as clinicopathological features such as age, stage, and histological type can predict poor or better prognosis of neuroblastoma tumor. The near-triploidy and the high expression of TRKA and TRKC are significantly correlated with better prognosis in stage 4S neuroblastoma, which is frequently associated with spontaneous regression [37,38]. Apoptosis, differentiation, T cell-mediated immunity, nerve growth factor (NGF) deprivation, impaired DNA repair, epigenetic control, and telomere shortening are among the main potential mechanisms of spontaneous regression in neuroblastoma [37].

The UPR after the occurrence of ER stress promotes the survival of neuroblastoma cells under normal conditions [39]. Upon stimulation of intense and prolonged ER stress, activation of the UPR pathway results in the death of neuroblastoma cells through cytotoxic autophagy and apoptosis [40]. However, autophagy promotes the survival of neuroblastoma cells by preventing cell death after ER stress. The autophagy system can operate in the degradation of unfolded proteins accumulated in the ER lumen in response to ER stress [41]. Autophagy is detected at basal levels in neuroblastoma patients and cell lines. It increases in stage 4 neuroblastoma compared to stage 4S, whereas apoptosis decreases in stage 4 neuroblastoma compared to stage 4S. In vitro experiments showed that autophagy increased after chemotherapeutic drug treatment in neuroblastoma cells. In addition, the inhibition of autophagy reduces tumor growth in mice treated with a chemotherapeutic agent. These findings suggest that autophagy is associated with chemoresistance in neuroblastoma, promoting the survival of tumor cells [42]. ER stress induced by prolonged treatment with thapsigargin and brefeldin A in non-*MYCN*-amplified SH-SY5Y cells augments cell death via the extracellular signal-regulated kinase (ERK) activation involving a caspase-independent pathway [43]. These findings suggest that exaggerated ER stress involving the accumulation of unfolded or misfolded proteins along with impaired autophagy may switch the course of the disease from malignant transformation to spontaneous regression through the induction of apoptosis in neuroblastoma.

Together, the published literature indicates that cell death, impaired autophagy, and immunity play crucial roles in both neurodegeneration that occurs in neurodegenerative diseases affecting the mature CNS neurons, which differentiate from neuroblasts arising from the division of the neural tube-derived neuroepithelial stem cells, and spontaneous regression of neuroblastoma developing from primitive sympathetic neuroblasts arising from neural crest-derived progenitor cells. However, a decline in adult neurogenesis, a process involved in the differentiation of neuroblasts derived from neural stem cells into mature neurons, is identified in neurodegenerative diseases, possibly exacerbating neurodegeneration. Conversely, neuronal differentiation of neuroblastoma cells represents a potential mechanism that contributes to the spontaneous regression of neuroblastoma. On the other hand, the normal aging brain does not show massive neuron loss. The neuroprotective and/or compensatory mechanisms may play important roles in resisting neurodegeneration in a healthy aging brain. Some mechanisms may be involved in a special gender-dependent differential gene expression pattern during brain aging. Gender-specific differences in the expression level of genes linked to the survival of neurons from certain brain regions during aging may decrease or increase the risk of developing a neurodegenerative disease associated with the brain region showing differential expression of that gene in females or males.

The present review contains a comprehensive survey and critical analysis of the published literature concerning the molecular and cellular fundamentals of normal brain aging, neurodegenerative diseases, AD, PD, dementia with Lewy bodies (DLB), HD, ALS, and MS, as well as neuroblastoma with spontaneous regression. Furthermore, by analyzing the Gene Expression Omnibus (GEO) dataset, the search tool for the retrieval of interacting genes/proteins (STRING) functional enrichments, and available scientific publications, differential gene expressions and associated molecular and cellular mechanisms of the normal aging brains of females and males are investigated in postmortem tissue samples dissected from multiple brain regions of females and males aged 71 years and over compared to females and males aged 40-70 years, respectively. Through the analysis of the differentially expressed genes (DEGs) of two GEO datasets including controls of AD and scientific publications, potential molecular and cellular mechanisms associated with the DEGs, which are involved in Aβ and tau pathologies, impaired autophagy, synaptic dysfunction, neuronal cell death, neurodegeneration, cognitive decline, and repressed adult neurogenesis, are identified in the entorhinal cortex (EC) and/or hippocampus (HC) of females and males with AD. Finally, the DEG analysis of a GEO dataset including microarray data of primary tumors of patients with stage 4S and stage 4 neuroblastoma and the assessment of the published papers provide further insights into potential mechanisms underlying spontaneous regression, such as apoptosis, neuronal differentiation, immune response, impaired autophagy, and impaired double-strand break (DSB) repair in stage 4S neuroblastoma.

## Review

Gender- and region-specific differential gene expressions and related molecular and cellular mechanisms of the normal aging human brain

Forebrain regions exhibit age-dependent changes in gene expression profiles. Superior frontal gyrus (SFG), postcentral gyrus (PCG), HC, and EC have a prominent differential gene expression pattern, particularly in persons aged 60-79 years compared to those aged 40-59 years. PCG, HC, and EC show greater numbers of downregulated genes in this period, whereas the number of upregulated genes is a little higher compared to that of downregulated genes in SFG. PCG and HC differentially express many genes in persons aged 40-59 years compared to those aged 20-39 years as well. PCG has about an equal number of downregulated and upregulated genes in huge numbers in persons aged 40-59 years. On the other hand, HC has dominantly downregulated gene expression in persons aged 40-59 years compared to those aged 20-39 years. There are remarkable gender-specific differences. The number of differential gene expressions in the brains of persons aged 60-79 years is much higher in males than in females, whereas females have excessively higher DEG numbers compared to males in persons aged 80 years and over [44].

A GEO dataset (GSE46706) includes gene expression microarray data derived from Affymetrix Human Exon 1.0 ST Array (transcript (gene) version) platform (GPL5175) of postmortem tissue samples dissected from up to 10 brain regions from 134 neurologically and neuropathologically normal human individuals [45-48].

Using the GEO2R tool, an R-based web application [45], the analysis of gene expression data generated via the GPL5175 microarray platform of the GSE46706 dataset exhibited that the frontal cortex (FC) [F, *n* (40-70 years/≥ 71 years) = 15/15; M, *n* = 59/18], occipital cortex (F, *n* = 16/15; M, *n* = 59/19), temporal cortex (TC) (F, *n* = 14/15; M, *n* = 51/22), thalamus (F, *n* = 15/14; M, *n* = 56/19), and medulla (F, *n* = 16/11; M, *n* = 55/17) in both genders aged 71 years and over compared to persons aged 40-70 years did not show a remarkable differential gene expression pattern meeting criteria of adjusted *p* ≤ 0.05 and │log2FC│ ≥ 1.0 (Data Appendix B, Data S1-S10). In addition, males did not demonstrate a differential gene expression pattern in the HC (*n* = 58/18) and substantia nigra (SN) (*n* = 42/18) (Data Appendix B, Data S11 and S12). The analysis also revealed that females did not have a differential gene expression in the white matter (WM) (*n* = 15/15) (Data Appendix B, Data S13).

**Figure 1:**
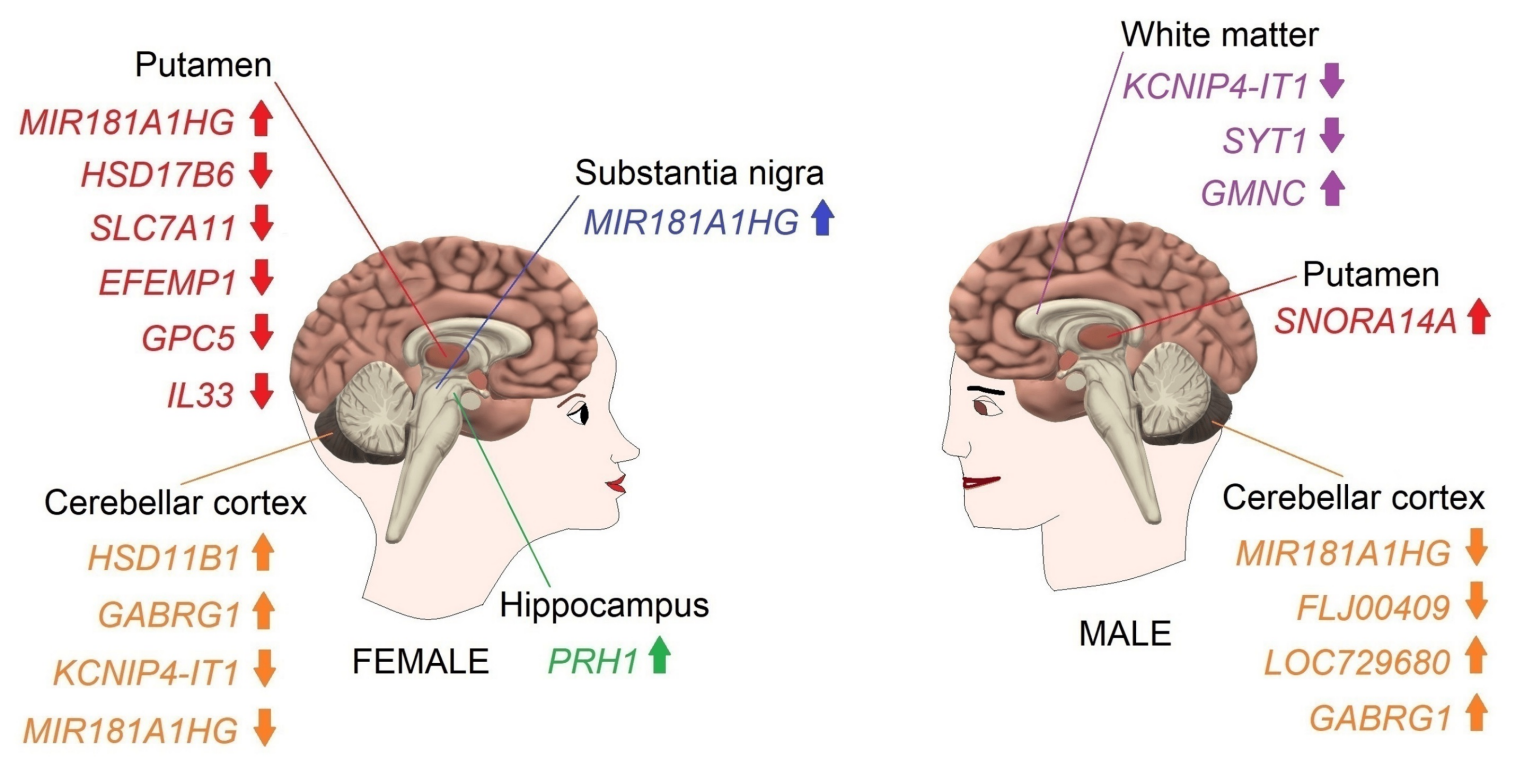
Differential gene expressions in postmortem tissue samples dissected from brain regions of neurologically and neuropathologically normal elderly adults compared to middle-aged adults Image created by the author N.A. solely for the present study using the Microsoft Paint and Paint 3D programs (Microsoft Corp., USA) The GEO2R analysis of gene expression data generated using the GPL5175 microarray platform of the GSE46706 dataset showed that the CC, HC, PUT, SN, and WM had significant differential gene expressions meeting criteria of adjusted *p* ≤ 0.05 and │log2FC│ ≥ 1.0 in females and/or males aged 71 years and over (elderly adults) compared to persons aged 40-70 years (middle-aged adults). The arrows represent downregulated (↓) and upregulated (↑) gene expressions. HC: hippocampus, CC: cerebellar cortex, PUT: putamen, WM: white matter

The GEO2R analysis exhibited that the cerebellar cortex (CC), HC, putamen (PUT), SN, and WM demonstrated significant differential gene expressions meeting criteria of adjusted *p* ≤ 0.05 and │log2FC│ ≥ 1.0 in females and/or males aged 71 years and over compared to persons aged 40-70 years (Table 1 and Data Appendix B, Data S14-S20). Older females and males had some DEGs in the CC and PUT, whereas only older females had a DEG in the HC and SN. Older males differentially expressed some genes in the WM as well (Figure 1).

**Table 1:** Differential gene expressions of neurologically and neuropathologically normal individuals aged 71 years and over compared to persons aged 40-70 years in aging brain regions The arrows represent downregulated (↓) and upregulated (↑) expressions. Using the GEO2R tool, gene expression data (GSE46706 dataset/GPL5175 platform) of elderly and middle-aged adults were compared separately for females and males. The selected options of GEO2R analysis are Benjamini & Hochberg False Discovery Rate for applying adjustment to the *p*-values, auto-detect for applying log transformation to the data, yes for applying limma precision weights (vooma), and yes for force normalization. The DEGs were determined based on the criteria of adjusted *p* ≤ 0.05 and │log2FC│ ≥ 1.0. *The values in parentheses of females and males represent the total numbers of individuals aged 40-70 years (group 1) versus 71 and over (group 2). The log2 fold change (log2FC) value shows a logarithmic fold change of differential gene expression. A negative log2FC value means that the expression of the gene is upregulated in group 2, whereas a positive log2FC value indicates that gene expression is downregulated in group 2. Chromosome locations were obtained from the gene database of NCBI [49]. CC: cerebellar cortex, F: female, HC: hippocampus, M: male, PUT: putamen, SN: substantia nigra, WM: white matter

Brain region	HC	CC	CC	SN	PUT	PUT	WM
Gender*	F (15/11)	F (16/15)	M (56/23)	F (11/14)	F (16/13)	M (59/21)	M (58/23)
Gene	PRH1	HSD11B1	MIR181A1HG	MIR181A1HG	EFEMP1	SNORA14A	KCNIP4-IT1
		GABRG1	LOC729680		MIR181A1HG		SYT1
		KCNIP4-IT1	GABRG1		IL33		GMNC
		MIR181A1HG	LOC643475		HSD17B6		
					SLC7A11		
					GPC5		
Accession	BC031043	BC012593	EF413001	EF413001	BC098561	NR_002955	NR_002813
		NM_173536	AK090664		EF413001		BC058917
		NR_002813	NM_173536		BC047085		NM_001146686
		EF413001	AK090485		NM_003725		
					NM_014331		
					NM_004466		
Expression	↑	↑	↓	↑	↓	↑	↓
		↑	↑		↑		↓
		↓	↑		↓		↑
		↓	↓		↓		
					↓		
					↓		
Adjusted P	0.0442	0.00354	2.27e-09	0.0052	0.01330	0.0018325	0.0079314
		0.00495	8.41e-09		0.01330		0.0120437
		0.00671	1.39e-07		0.01796		0.0170431
		0.02083	2.48e-06		0.01796		
					0.03493		
					0.05001		
Log2FC	-1.037	-1.185	1.056	-1.248	1.160	-1.151	1.039
		-1.106	-1.028		-1.113		1.004
		1.051	-1.057		1.094		-1.076
		1.193	1.172		1.004		
					1.115		
					1.012		
Location [49]	12p13.2	1q32.2	1q32.1	1q32.1	2p16.1	7q11.23	4p15.2
		4p12	13q12.11		1q32.1		12q21.2
		4p15.2	4p12		9p24.1		3q28
		1q32.1	10p12.2		12q13.3		
					4q28.3		
					13q31.3		
Strand	−	+	−	−	−	+	−
		−	−		−		+
		−	−		+		−
		−	−		+		
					−		
					+		

A Potential Neuroprotective Role for PRH1 Against Hippocampal Neurodegeneration in Elderly Females During Normal Brain Aging

**Figure 2:**
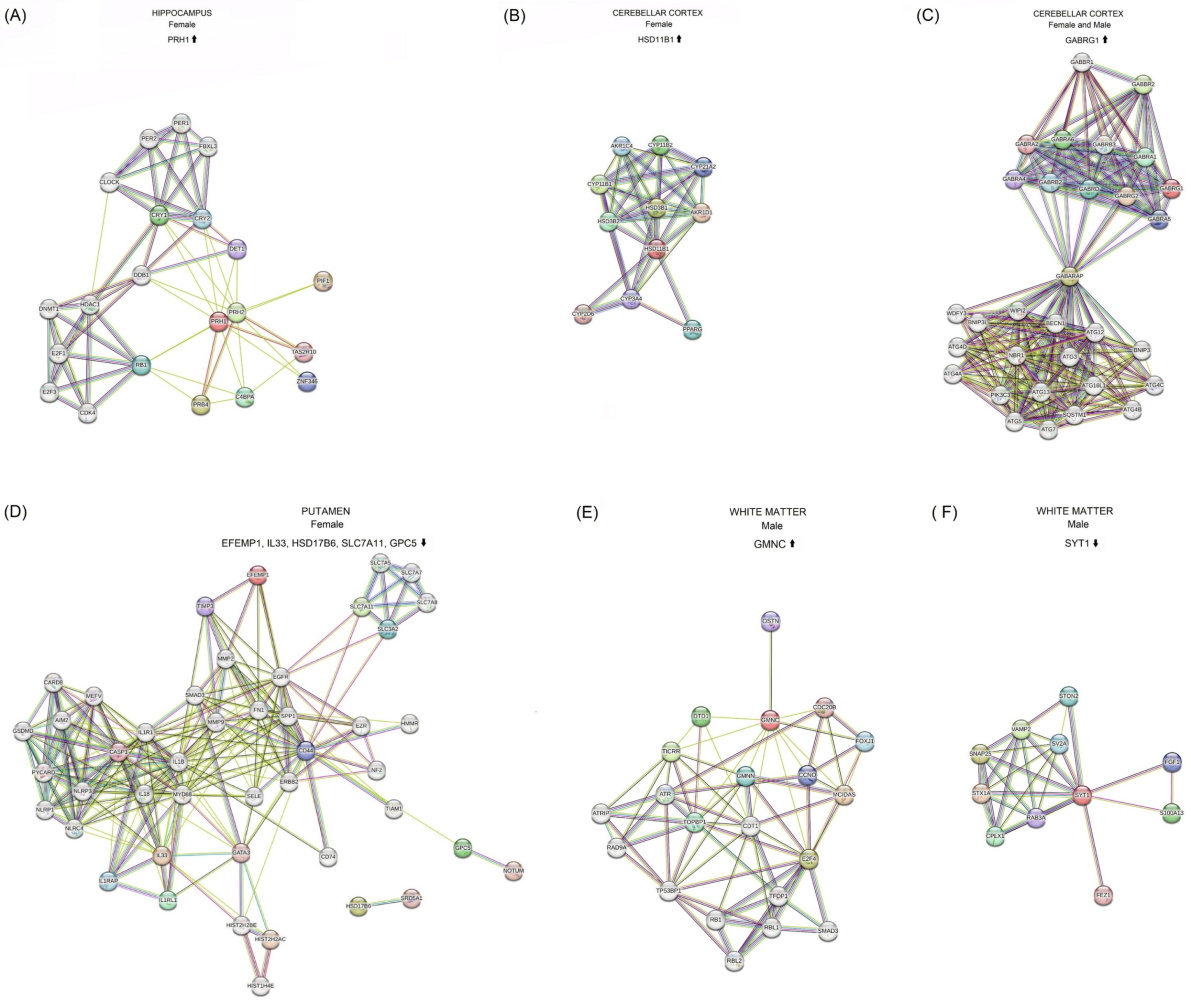
Functional enrichment analyses of the DEGs carried out using the STRING database Image created by the author N.A. solely for the present study using the Microsoft Paint program (Microsoft Corp., USA) Complex interaction networks of protein products of genes showing differential expression in the HC, CC, PUT, and WM in elderly females and/or males during normal brain aging are presented: (A) PRH1, (B) HSD11B1, (C) GABRG1, (D) EFEMP1/IL33/HSD17B6/SLC7A11/GPC5, (E) GMNC, and (F) SYT1. The main biological processes and network stats, including the *p*-value of functional enrichments, are presented in Appendices A-F. DEG: differentially expressed gene, HC: hippocampus, CC: cerebellar cortex, PUT: putamen, WM: white matter

The GEO2R analysis of the GSE46706 dataset demonstrated that the HC upregulated the expression of proline-rich protein HaeIII subfamily 1 (BC031043, *PRH1*) in females aged 71 years and older compared to females aged 40-70 years (Table 1 and Figure 1). PRH1 is a member of the human salivary proline-rich protein family. Proline-rich proteins have a protective effect on the tooth surface [50,51]. Not surprisingly, the salivary gland is the main site for the expression of *PRH1*. In the brain, it is mostly expressed in the WM, cerebral cortex, pons, and medulla oblongata, followed by the HC [52]. Functional enrichment analysis of PRH1 performed using the STRING database demonstrates that the glucocorticoid receptor signaling pathway, transcription involved in the G1/S transition of the mitotic cell cycle, and fat cell proliferation constitute the top three biological processes that are negatively regulated by a PRH1-centered protein network [53,54] (Figure 2A, Appendix A).

Glucocorticoids can exert pro-inflammatory or anti-inflammatory effects depending on the site of action, dose, and chronicity of exposure. Glucocorticoids can promote the anti-inflammatory response in both peripheral organs and the brain. The basal levels of glucocorticoids suppress the pro-inflammatory signaling regulated by nuclear factor-kappa B (NF-κB) via recruiting co-repressors of the pro-inflammation pathway upon binding to their glucocorticoid receptors. However, its higher levels can lead to a pro-inflammatory response in the brain. The prolonged exposure to glucocorticoids enforces NF-κB-mediated pro-inflammatory cytokine signaling through binding to the glucocorticoid receptors. The *in vivo* studies exhibit that chronic stress levels and prolonged exposure to glucocorticoids cause microglial activation and neuroinflammation response in the HC, resulting in neuronal loss. Glucocorticoids can inhibit neurogenesis and display pro-apoptotic effects in the HC. Glucocorticoids may trigger cognitive decline through dendritic atrophy and loss of synaptic connections of neurons in the HC [55-62].

Postmitotic neurons permanently enter the G0 phase of the cell cycle and remain terminally differentiated. Dysregulation of proteins controlling the cell cycle in postmitotic neurons may cause cell cycle re-entry followed by apoptotic cell death in pathological conditions. Upregulation of cyclins and increased activity of cyclin-dependent kinases (CDKs), particularly activation of the complex composed of cyclin D and its partners CDK4 and CDK6, are associated with neuronal apoptosis. Elevated expression of cyclin D ensures the transition from G0 to the G1 phase. The activated cyclin D-CDK4/6 complexes repress retinoblastoma 1 (RB1) through its phosphorylation, thereby derepressing E2F transcription factor 1 (E2F1). In this way, E2F1 induces the G1/S transition; however, it promotes apoptotic cell death instead of entry into mitosis in postmitotic neurons [63]. During the normal development of the nervous system, special neuronal cell populations such as retinal ganglion cells and cortical neurons undergo DNA replication after the cell cycle re-entry, resulting in tetraploidization with 4C DNA content. These tetraploid neurons persist throughout adult life. Moreover, cell cycle re-entry and tetraploidy are considered early hallmarks of neurodegeneration in neurodegenerative diseases. Tetraploid neurons are more susceptible to cell death compared to diploid neurons during the development of disease [64]. Tetraploidization is observed in superficial and deep cortical neurons of the cerebral cortex of wild-type mice during normal aging. E2F1 is required for the process of tetraploidization during DNA replication. Tetraploid neurons also show an increase in the normal human EC during aging. Notably, the proportion of tetraploidization of neurons in the FC significantly increases in Braak stage II before the formation of neurofibrillary tangles (NFTs) in AD patients. There is a significant correlation between neuritic plaque burden and tetraploidy levels of neurons in the FC of AD brains [65]. These findings suggest that tetraploidy may contribute to the pathogenesis of AD, probably by enhancing sensitivity to cell death.

A mouse model with hyperlipidemia demonstrates that lipid accumulation in the CA3 region of the HC leads to caspase-3-mediated apoptosis of hippocampal neurons. Hyperlipidemia also elevates the expression of beta-secretase 1 (BACE1). BACE1 enzyme catalyzes the cleavage of amyloid precursor protein (APP), giving rise to Aβ production. These results indicate that fat deposition in the HC may be a crucial risk factor for developing AD [66].

**Figure 3:**
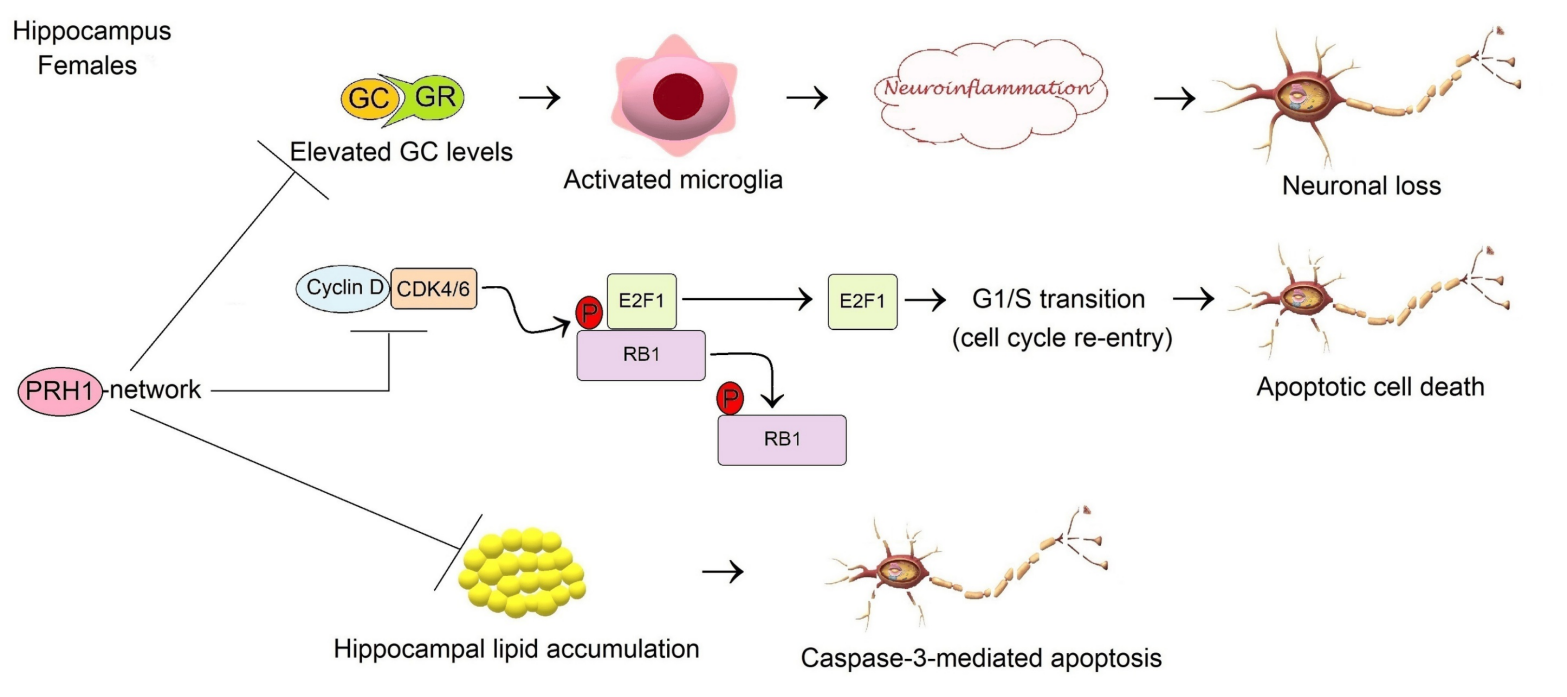
Potential neuroprotective mechanisms of PRH1-centered network against neurodegeneration in the HC of elderly females during normal brain aging Image created by the author N.A. solely for the present study using the Microsoft Paint and Paint 3D programs (Microsoft Corp., USA) The STRING database [54] and literature suggested that PRH1 was primarily implicated in the control of the glucocorticoid receptor (GR) signaling pathway, the G1/S transition, and hippocampal lipid accumulation. The PRH1-driven signal transduction network may suppress microglia-mediated neuroinflammation occurred due to elevated glucocorticoid (GC) levels, leading to neuronal loss [55-62]. On the other hand, the PRH1-involved network may prevent apoptotic neuron death through repression of cell cycle re-entry. Cyclin D/CDK4/6 complex derepresses E2F1 by phosphorylating RB1, in turn, E2F1 mediates G1/S transition, resulting in apoptosis [63]. PRH1 is also likely to be involved in the regulation of hippocampal lipid accumulation that induces caspase-3-mediated apoptosis of neurons in the hippocampus (HC) [66].

In light of available literature, the GEO2R analysis of microarray gene expression data generated using the GPL5175 platform of the GSE46706 dataset and functional enrichments based on the STRING database suggest that upregulation of *PRH1* may play a neuroprotective role against hippocampal neuron loss through negative regulation of glucocorticoid receptor signaling pathway, G1/S transition, and lipid accumulation in females aged 71 years and older (Figure 3). However, further research is needed to uncover whether PRH1 is involved in the mechanisms controlling the plasticity and integrity of postmitotic neurons in brain tissues during normal aging.

Differential Gene Expressions in the Cerebellar Cortex of Elderly Females and Males During Normal Brain Aging

The cerebellum (CER) makes up about 10% of brain mass, but it has the vast majority (~80%) of neurons located in the brain. Because the CER contains abundantly small tightly packed granule neurons arranged in the innermost granular layer of the CC covering its internal WM [67]. The CER coordinates both motor and non-motor functions, including coordination, balance, cognition, comprehension, vision, audition, and language. The CC is composed of three layers termed the molecular layer, the Purkinje cell layer, and the granular cell layer, arranged from the outermost to the innermost. Nine neuron types have been identified in the CC: stellate cells and basket cells in the molecular layer, Purkinje cells and candelabrum cells in the Purkinje cell layer, and granule cells, globular cells, Lugaro cells, Golgi cells, and unipolar brush cells in the granular layer [68]. The gray matter constitutes the CC located in the outer layer of the CER [69]. A meta-analysis demonstrates that cerebellar gray matter atrophy occurs in the brains of neurodegenerative AD, ALS, FTD, multiple system atrophy, and PSP patients. The pattern of the atrophy is highly disease-specific with characteristic cortical or subcortical changes for each condition [70].

The GEO2R analysis of gene expression data generated using the GPL5175 microarray platform of the GSE46706 dataset revealed that females aged 71 years and over, compared to females aged 40-70 years, had differential expressions meeting criteria of adjusted *p* ≤ 0.05 and │log2FC│ ≥ 1.0 for four genes in the CC (Table 1, Figure 1). The analysis demonstrated that hydroxysteroid 11-beta dehydrogenase 1 (BC012593, *HSD11B1*) and GABA type A receptor subunit gamma 1 (NM_173536, *GABRG1*) were upregulated, whereas KCNIP4 intronic transcript 1 (NR_002813, *KCNIP4-IT1*; also known as *NCRNA00099*) and MIR181A1 host gene (EF413001/LOC100131234, *MIR181A1HG*; also known as familial acute myelogenous leukemia related factor) were downregulated.

In addition, the GEO2R analysis of gene expression data produced using the same platform (GPL5175) exhibited that males aged 71 years and older, compared to males aged 40-70 years, showed differential expressions meeting criteria of adjusted *p* ≤ 0.05 and │log2FC│ ≥ 1.0 for four genes in the CC (Table 1, Figure 1). According to the results of this analysis, upregulated genes were uncharacterized LOC729680 (AK090664, *LOC729680*) and *GABRG1* (NM_173536), whereas downregulated genes were *MIR181A1HG* (EF413001) and FLJ00409 protein (AK090485, *LOC643475*).

Taken together, the CC data showed that females and males aged 71 years and older, compared to females and males aged 40-70 years, respectively, had two common DEGs: upregulated *GABRG1* and downregulated *MIR181A1HG*. However, downregulated *KCNIP4-IT1* (females) and *LOC643475* (males) and upregulated *HSD11B1* (females) and *LOC729680* (males) were not common DEGs shared by females and males.

Potential Involvement of HSD11B1 and Dysregulated Glucocorticoid Signaling in the Formation of Age-Related Cerebellar Motor Deficits in Elderly Females During Normal Brain Aging

The GEO2R analysis of gene expression data generated using the GPL5175 microarray platform of the GSE46706 dataset demonstrated that *HSD11B1* was upregulated in the CC of females aged 71 years and over compared to females aged 40-70 years (Table 1, Figure 1). *HSD11B1* (also known as *11β-HSD1*) is expressed in the HC, prefrontal cortex (PFC), and CER samples obtained from human postmortem brain tissues lacking evidence of CNS disorders. Its higher expression is detected in the hippocampal CA3, dentate gyrus (DG), granule cell layer of the CC, and all areas of the PFC [71]. *HSD11B1* has also been reported to be highly expressed in the CER, HC, and cortex of adult rats. It is upregulated in particular Purkinje cells of the CC, hippocampal CA3 pyramidal cells, and layer IV of the parietal cortex [72,73]. The mRNA expression of *HSD11B1* is elevated with age in the CA3 region of the HC and layer V of the parietal cortex in mice. This increase is significantly correlated with cognitive decline [74]. Glucocorticoids show intracellular local amplification, particularly in the HC and cortex cells, upon the action of HSD11B1 in the aging brain, leading to impairment in spatial learning [75]. Functional enrichment analysis [53,54] demonstrated that HSD11B1 was mainly involved in biological processes related to biosynthetic processes of mineralocorticoid, cortisol, aldosterone, and glucocorticoid (Figure 2B and Appendix B).

The mouse experiments exhibit that the oscillation of glucocorticoids inhibits the activation and proliferation of neural stem/precursor cells via glucocorticoid receptors in the HC of the aging brain. Glucocorticoids suppress adult hippocampal neurogenesis by regulating the methylation of specific gene promoters. Notably, the disruption of glucocorticoid oscillation induces the formation of newborn granule neurons derived from neural stem cells. These findings suggest that glucocorticoid oscillations may contribute to age-dependent cognitive decline by repressing adult hippocampal neurogenesis [76].

Glucocorticoids induce apoptotic death of neural progenitor cells by stimulating their glucocorticoid receptors in the external granule cell layer of the developing CER in the neonatal mouse. The apoptosis of neural progenitor cells results in a permanent decrease in the cell counts of internal granule layer neurons in the CC [77].

Exogenous glucocorticoid (corticosterone, CORT) administration upregulates the expression of the glucocorticoid receptor and *HSD11B1* in the CER of adult mice. The CORT impairs motor coordination, possibly through repression of the corticotropin-releasing hormone (CRH)/corticotropin-releasing hormone receptor 1 (CRH-R1) system in the CER [78]. The immunoreactivity and mRNA expression of the glucocorticoid receptor are moderately to strongly detected in the Purkinje cell layer and granule cell layer of the CC in the adult rat brain [79]. Intermittent treatment of CRH increases dendritic outgrowth and elongation of Purkinje cells in cerebellar slice cultures of rat CER [80]. CRH also promotes the survival of GABAergic interneurons via its CRH-R1 receptor in primary cell cultures established from mice CER [81].

The wild-type form of estrogen receptor 2 (ESR2, also known as ER-β) is predominantly expressed in the Purkinje cells and scattered large cells of the granule cell layer in the CER of both female and male adult rats, suggesting that the cerebellar neurons with the expression of ER-β are most likely to be targeted by the estrogens [82]. The cerebellar motor learning is more enhanced in female mice that produce high levels of endogenous 17β-estradiol (E2) compared to female mice producing low levels of E2 or male mice. E2 elevates the induction of long-term potentiation (LTP) in Purkinje cells and improves cerebellar plasticity and synaptic formation, thereby strengthening motor skills [83]. The production and level of E2 in women are prominently reduced during menopause. The proportion of women whose serum E2 levels are below the limit of detection increases gradually with advancing age among women aged 70 years and over [84]. The 24-hour urinary-free cortisol to creatinine ratio (UFC/Cr) shows a U-shaped trend that increases progressively, especially after age 60, throughout the human life span. The UFC/Cr is modestly higher in women than in men [85].

Mice experiments demonstrate that stereological neuron counts exhibit a significant loss of Purkinje neurons in 18- to 24-month-old mice during normal aging. Interestingly, the number of hippocampal pyramidal neurons remains unchanged in the same mice. In addition, long-term depression (LTD) impairment in the CER appears in four- to eight-month-old mice, suggesting that synaptic loss may trigger neuronal loss. Age-dependent cerebellar learning and memory deficits are most likely to occur due to the loss of Purkinje neurons and synaptic plasticity changes [86].

The senescence-accelerated mouse-prone 8 (SAMP8) strain, a mouse model of accelerated aging, exhibits that the brains of four- and seven-month-old SAMP8 mice show apoptotic death of Purkinje cells in the CER. The brains of the senescence-accelerated mouse resistant 1 (SAMR1) mice, control animals of normal aging, also have apoptotic Purkinje cells in the CER. The age-related increase in apoptotic death of Purkinje cells depends on the activation of the caspase-3 enzyme. The increase in apoptotic Purkinje cells of SAMP8 and SAMR1 mice is associated with a decrease in the level of tyrosine hydroxylase and choline acetyltransferase enzymes. However, the level of tyrosine hydroxylase is comparatively more reduced compared to that of choline acetyltransferase, indicating that apoptotic Purkinje cells can be primarily catecholaminergic neurons [87].

Overall, the literature, GEO2R analysis, and functional enrichment method suggest that upregulation of *HSD11B1* and dysregulation of glucocorticoid signaling may be implicated in the formation of age-dependent motor deficits through cerebellar gray matter atrophy, especially involving Purkinje cell loss, during normal brain aging. The abrogation of cerebellar plasticity and synapse homeostasis may cause caspase-3-dependent apoptotic death of particularly Purkinje cells and therefore age-related impairment in cerebellar motor coordination and motor learning in the absence of the neuroprotective effects of E2/ER-β signaling on the CC neurons of the CER in elderly females.

A Possible Novel Role for GABRG1 to Compensate for Compromised GABAergic Transmission in the Aging Cerebellar Cortex

Gamma-aminobutyric acid (GABA) functions as a primary inhibitory neurotransmitter in the human adult brain [88]. Loss of GABAergic neurons and synapses, the reduced response rate of postsynaptic neurons, or failure of neurotransmitter release cause an imbalance between inhibitory and excitatory neurotransmission, resulting in impaired neuronal plasticity. If compensatory mechanisms are impaired as well, neurodegenerative conditions may occur. However, the GABAergic system can show more resistance against neurodegeneration in contrast to cholinergic and glutamatergic systems [89,90]. GABA_A_ and GABA_B_ receptors mediate the inhibitory effects of GABA in the CNS. GABA provides chloride ion influx and hyperpolarizes the membrane via binding to GABA_A_ receptors, decreasing neuronal excitability. GABA_A _receptors serve as ligand-gated chloride ion channels in the human mature brain. The GABA_A_ receptor family is composed of pentameric structures assembling different combinations of subunits. GABRG1 is one of three gamma-type GABA_A_ receptor subunits [88,91].

The protein expression of *GABRG1* is mostly observed in the basal ganglia, cerebral cortex, and CER, whereas it is less frequently expressed in the HC. The mRNA expression of *GABRG1* is detected in all brain regions, including the CER. The thalamus, midbrain, hypothalamus, cerebral cortex, medulla oblongata, pons, and WM are the leading sites of expression [52].

The GEO2R analysis of gene expression data generated using the GPL5175 microarray platform of the GSE46706 dataset revealed that the mRNA expression of *GABRG1* was significantly upregulated in the CC of females and males aged 71 years and over compared to females and males aged 40-70 years, respectively (Table 1, Figure 1). The impairment of GABAergic transmission during aging can trigger some compensatory mechanisms such as upregulation of the α1 subunit expression of postsynaptic GABA_A_ receptors, thereby providing higher sensitivity of GABA_A_ receptors to GABA in the aged rat HC [89,90,92], suggesting that dysregulation of GABAergic system may also be compensated through upregulation of the expression of some GABA_A _receptor subunits including GABRG1 in the CC during normal brain aging. A compensatory mechanism involving the GABA_A_ receptors with overexpression of GABRG1 may contribute to the GABAergic system-mediated resistance against neurodegeneration. Upon the decrease in GABAergic input during brain aging, the sensitivity of GABA_A_ receptors to GABA is likely to be elevated through the compensatory increase of the GABRG1 subunit within the mature GABA_A_ receptor.

Functional enrichment analysis [53,54] demonstrated that GABRG1 was primarily implicated in biological processes related to C-terminal protein lipidation, protein lipidation involved in autophagosome assembly, piecemeal microautophagy of the nucleus, GABAergic synaptic transmission, aggrephagy, autophagy of nucleus, inhibitory synapse assembly, GABA signaling pathway, mitophagy, and mitochondrial fragmentation involved in the apoptotic process (Figure 2C and Appendix C).

The autophagic process eliminates many cellular components, such as unnecessary cytoplasmic structures, defective organelles, and misfolded proteins, via lysosomes. There are different types of autophagy, such as macroautophagy (simply termed autophagy) with general bulk degradation, microautophagy, including mitophagy of mitochondria and nucleophagy of the nucleus, and CMA, which use selective or non-selective mechanisms. Autophagy has a crucial function for neurons, which are long-lived postmitotic cells. It destroys misfolded proteins, prolonging the survival of neurons [93,94]. An *in vivo* study with adolescent mice exhibits that the deletion of autophagy-related gene 7 (*ATG7*) in forebrain neurons leads to a deficiency in the autophagy process, causing aggregation of sequestosome 1 (SQSTM1/p62) adaptor protein. This adaptor protein plays a role in loading ubiquitinated substrates into autophagosomes. The levels of p62 are used as a marker for detecting defective autophagy. Its aggregates sequester GABA type A receptor-associated protein (GABARAP) family proteins, resulting in decreased surface expression of GABA_A_ receptors and the impairment of inhibitory GABAergic signaling. Hyperactivation of the mechanistic target of rapamycin kinase (mTOR) in neurons also abrogates GABAergic signaling. These findings indicate that an efficient autophagic process is associated with functional GABAergic signal transduction [95,96].

However, yeast experiments demonstrate that increases in the level of GABA cause the inhibition of mitophagy and pexophagy, elevating oxidative stress. In addition, the mouse experiments show that the accumulation of GABA augments mitochondria numbers due to the inhibition of mitophagy in the brain. Increased mitochondrial numbers form excessive reactive oxygen species, leading to intracellular oxidative stress. Cell death occurs through GABA-induced oxidative stress due to the inhibition of mitophagy [97].

The knockdown of lysosome-associated protein 2A (LAMP2A), which is a CMA protein, performed using the microRNA (miRNA) in the CC neurons of mice causes the loss of Purkinje cells, granule cells, satellite cells, and basket cells, resulting in progressive motor impairment. These findings demonstrate that defective CMA leads to neurodegeneration in the CC [98]. In addition, a mouse model of Purkinje cell degeneration shows that the loss of Purkinje cells occurs via a dysregulated autophagy pathway involving aberrant mitophagy [99].

The Purkinje cells are GABAergic inhibitory neurons. They receive both excitatory glutamatergic and inhibitory GABAergic inputs. The Purkinje cells have inhibitory effects on the cerebellar and vestibular nuclei neurons of the CER. The basket cells and stellate cells located in the molecular layer are inhibitory GABAergic interneurons. They receive excitatory inputs from parallel fibers. The stellate and basket interneurons show an inhibitory effect on the Purkinje cells. The granule cells are glutamatergic excitatory neurons of the CC [100].

Collectively, these findings suggest that increased GABAergic input may be implicated in neuronal cell death via dysregulated autophagy involving aberrant mitophagy, followed by the generation of oxidative stress in neurodegenerative conditions. However, an efficient autophagic process facilitates functional GABAergic signal transduction. Current research suggests that physiologically appropriate levels of GABA and functional autophagy are required for stabilizing the level of GABA receptors and ensuring efficient GABAergic transmission to sustain neuronal homeostasis. The imbalance between GABAergic, cholinergic, and glutamatergic neurotransmission may increase the sensitivity of neurons to neurodegeneration. During aging, the compromised GABAergic system and autophagic process, including mitophagy and CMA, may show adaptation to brain aging to slow the impairment in cerebellar plasticity and motor behavior. To increase the resistance against synaptic loss, GABRG1 may play a key role as part of a novel compensatory mechanism supporting the efficient autophagic process, possibly by increasing the sensitivity of GABA_A_ receptors to GABA during normal brain aging.

Potential Contribution of Reduced MIR181A1HG in the Cerebellar Cortex to the Age-Dependent Decline in Emotional Memory and Fear Learning

The GEO2R analysis of gene expression data derived from the GPL5175 microarray platform of the GSE46706 dataset exhibited that the expression of *MIR181A1HG* was significantly downregulated in the CC of females and males aged 71 years and over compared to females and males aged 40-70 years, respectively (Table 1, Figure 1). *MIR181A1HG*, a long noncoding RNA (lncRNA) gene, is the host gene for hsa‑miR‑181a‑1 and hsa‑miR‑181b‑1 [101,102]. The *MIR181A1* and *MIR181B1* genes are located in the intron of *MIR181A1HG* [101,103]. miR-181b represses the expression of *FAMLF* (or *MIR181A1HG*) through binding to its 5'-untranslated region (5'-UTR) in Burkitt lymphoma cells. There is a significant inverse correlation between *FAMLF* and miR-181b expression in Burkitt lymphoma patients [103]. In addition, the expression of the *FAMLF1* splice variant is significantly positively correlated with the expression of miR-181a in AML patients [104].

miR-181a is necessary for the formation of hippocampal long-term memory in mice [105]. A whole-brain functional magnetic resonance imaging (MRI) study exhibits that CER and corticocerebellar connections, including interactions related to the amygdala and HC, contribute to the formation of emotionally-enhanced memory in the human. The CER functions as an integral part of the emotionally-enhanced episodic memory encoding process [106]. An increased level of miR-181a has been shown to consolidate the contextual fear memory in the HC of adult mice [107]. The CER controls fear learning and memory formation [108]. The aged rat experiments demonstrate that contextual fear learning, emotional memory, and hippocampal LTP are abrogated during aging [109]. These findings suggest that the downregulation of *MIR181A1HG* and possibly *MIR181A1* in the CC may be linked to the decline in emotional memory and fear learning during brain aging.

Overexpression of miR-181a decreases the expression and aggregation of mutant ATXN3 protein via targeting its 3'-UTR sequences and thereby reduces neurodegeneration and neuroinflammation in the CC of transgenic mice in a lentiviral mouse model of SCA type 3 [110]. There is no statistically significant neuronal loss in most brain regions, such as the HC, TC, and FC, during normal aging [111]. The CC shows a volume decrease of about 16% with age. The mean perikaryon volume of Purkinje cells in the human CER significantly declines at a rate of 33.2% with age. Notably, there is a decrease of about 41% with age in the number of both Purkinje and granule cells in the anterior lobe [112].

The deletion of *ATG5* in the Purkinje cells of mice causes the swelling of axons followed by progressive neurodegeneration, suggesting that autophagy contributes to the conservation of axonal morphology and resistance of the Purkinje cells against neurodegeneration [113]. A mouse model of Purkinje cell degeneration demonstrates that the activation of the dysregulated autophagy pathway involving aberrant mitophagy mediates Purkinje cell death in the brain CER [99]. The apoptotic death that is dependent on caspase-3 of the Purkinje cells of the CER in the senescence-accelerated mouse (SAMP8) strain has also been reported [87]. miR-181a is one of the miRNAs protecting Purkinje cells against slow degeneration. A group of miRNAs, including miR-181a, cannot be detected in the Purkinje cells with a deficiency in the enzyme Dicer. Therefore, Purkinje cells undergo apoptosis in the CER. However, there are autophagic vacuoles together with the apoptotic phenotype as well [114].

**Figure 4:**
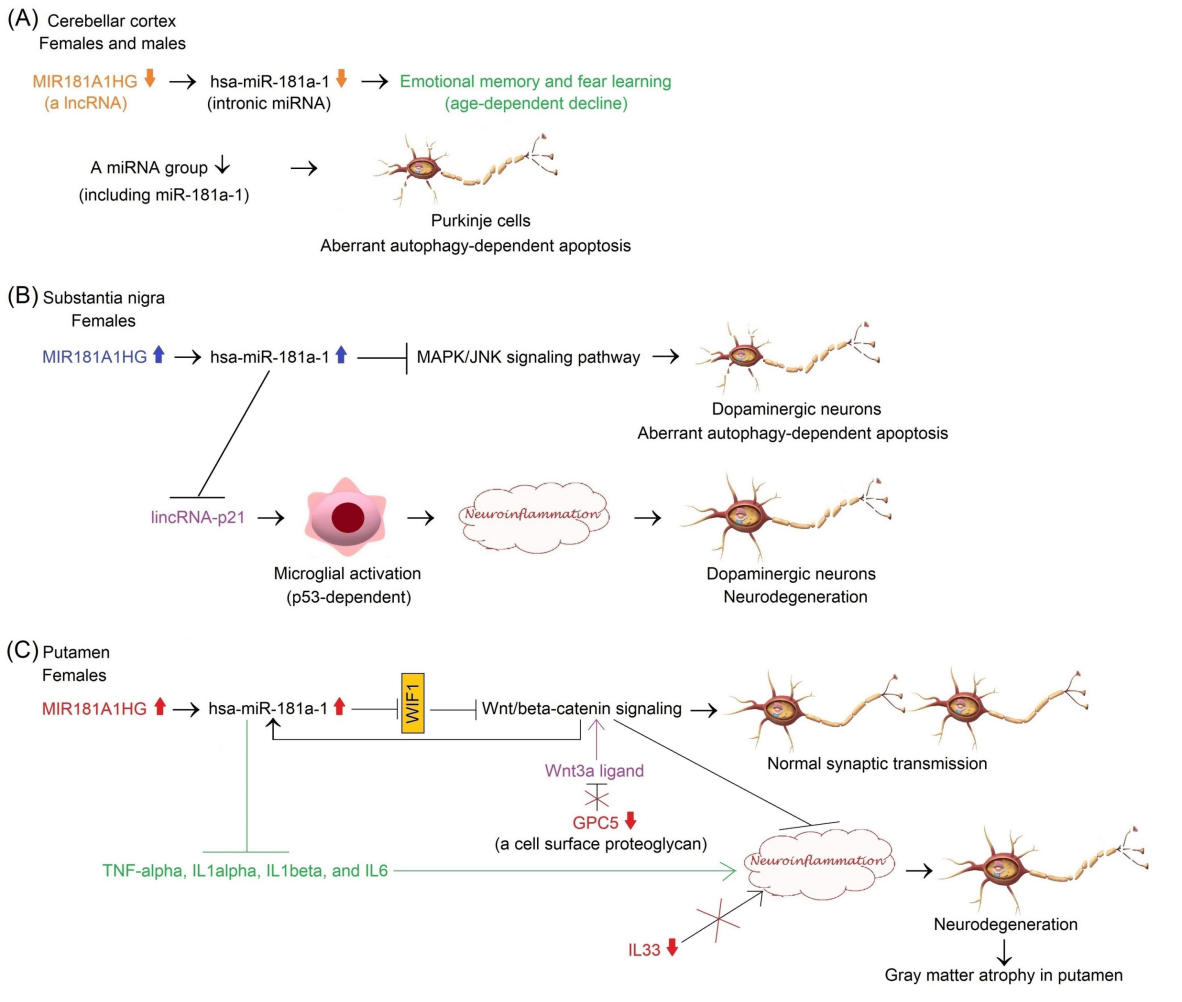
Potential mechanisms mediating the effects of differential expression of lncRNA MIR181A1HG and its intronic miRNA miR-181a-1 on neurodegeneration in the CC, SN, and PUT of elderly adults compared to middle-aged adults during normal brain aging Image created by the author N.A. solely for the present study using the Microsoft Paint and Paint 3D programs (Microsoft Corp., USA) (A) Downregulation of *MIR181A1HG* and possibly its intronic miRNA miR-181a-1 in the CC may mediate an age-dependent decline in emotional memory and fear learning in elderly females and males [105-109]. The decrease in the level of a miRNA group including miR-181a-1 can cause aberrant autophagy-dependent apoptosis of Purkinje cells [114]. (B) Upregulation of *MIR181A1HG* and possibly miR-181a-1 may inhibit aberrant autophagy-dependent apoptosis of dopaminergic neurons by suppressing the MAPK/JNK signaling pathway in the SN of elderly females [115]. Their upregulation may also prevent lincRNA-p21-mediated microglial neuroinflammation and neurodegeneration of dopaminergic neurons [116]. (C) Upregulation of *MIR181A1HG* and possibly miR-181a-1 may activate the Wnt/β-catenin signaling through repression of WIF1 and thereby support normal synaptic transmission in the PUT of elderly females [117-119]. The Wnt/β-catenin signaling suppresses neuroinflammation, suggesting that it may serve as a safeguard against neurodegeneration and gray matter atrophy in the PUT [120]. In addition, miR-181a functions as a key anti-inflammatory miRNA during inflammation [121]. Therefore, upregulation of miR-181a-1 may prevent neuroinflammation followed by neurodegeneration and gray matter atrophy in the PUT of females [122]. The GEO2R analysis of gene expression data generated using the GPL5175 microarray platform of the GSE46706 dataset also demonstrated that GPC5 and IL33 were downregulated in the PUT. The downregulation of GPC5, a cell surface proteoglycan [51], causes the activation of Wnt/β-catenin signaling due to the derepression of Wnt3a ligand [123,124]. On the other hand, the downregulation of IL33 leads to the suppression of neuroinflammation [125]. CC: cerebellar cortex, PUT: putamen

Taken together, decreased expression of *MIR181A1HG* and possibly *MIR181A1* in the aging CC may affect emotional memory and fear learning in elderly females and males (Figure 4A). However, the mechanisms underlying decreased expression of miR-181a-1 and its host gene on dysregulated autophagy pathway and apoptotic cell death of cerebellar Purkinje cells during normal brain aging remain to be fully elucidated.

A Potential Neuroprotective Role for MIR181A1HG in Resisting Neurodegeneration in the Substantia Nigra and Putamen of Elderly Females During Normal Brain Aging

However, the GEO2R analysis of gene expression data generated using the GPL5175 microarray platform of the GSE46706 dataset revealed that the SN and PUT significantly increased the expression of *MIR181A1HG*, in contrast to the CC, in females aged 71 years and over compared to females aged 40-70 years (Table 1, Figure 1). During normal brain aging, the number of pigmented dopaminergic neurons decreases with age at a rate of 4.7% per decade in caudal substantia nigra pars compacta (SNpc). The greatest loss of neurons happens in the dorsal tier of the SN at a frequency of 6.9% per decade [126]. Wild-type C57BL/6 mice experiments show that locomotor dysfunction is associated with the loss of dopaminergic neurons and a decrease in the levels of dopamine in aged mice. In addition, dopaminergic neurons in aged mice contain fragmented mitochondria, suggesting that mitochondrial damage may induce dopaminergic neuron loss during brain aging [127]. The dopaminergic neurons lacking PTEN-induced kinase 1 (*PINK1*) are lost due to mitochondrial apoptosis. These neurons have fragmented mitochondria as well [128]. The protein expression of parkin RBR E3 ubiquitin-protein ligase (PRKN/PARK2) is significantly downregulated in the SN, CER, striatum, and brain stem of aged male and female mice, showing age-dependent dopaminergic neuron loss. Restoring the PRKN expression using diaminodiphenyl sulfone (DDS) impedes age-related dopaminergic neuron loss. DDS generates mild ER stress, activating the PRKR-like endoplasmic reticulum kinase (PERK)/activating transcription factor 4 (ATF4)/PRKN axis in response to the UPR induction in the aging brains of old mice and SH-SY5Y cells. Mild ER stress prevents the loss of dopaminergic neurons, whereas severe ER stress triggers apoptotic cell death [129].

The PINK1/PRKN pathway controls mitophagy and apoptosis through polyubiquitination and monoubiquitination of voltage-dependent anion-selective channel protein 1 (VDAC1), respectively. The deficiency in polyubiquitination of VDAC1 suppresses mitophagy regulated by the PINK1/PRKN pathway in VDAC1 knockout mouse embryonic fibroblast cells, whereas defective monoubiquitination of VDAC1 promotes apoptotic cell death, accompanied by a decline in the number of dopaminergic neurons in transgenic flies expressing a mutant form of *Drosophila porin* that is the ortholog of human *VDAC1*. These findings indicate that the PINK1/PRKN pathway supports the survival of postmitotic dopaminergic neurons by removing damaged mitochondria via mitophagy and suppressing apoptosis [130].

A PD model of SK-N-SH neuroblastoma cells suggests that the expression of miR-181a is downregulated in PD. Overexpression of miR-181a attenuates neuronal apoptosis by repressing the p38 mitogen-activated protein kinase (MAPK)/c-Jun N-terminal kinase (JNK) signaling pathway and inhibits autophagy [115], suggesting that miR-181a may protect dopaminergic neurons against aberrant autophagy-dependent apoptotic cell death.

miR-181a mimic downregulates the expression of the inflammatory factors tumor necrosis factor-alpha (TNF-α), interleukin-1 alpha (IL1α), interleukin-1 beta (IL1β), and interleukin-6 (IL6) induced in monocytes and macrophages. This study provides evidence for the anti-inflammatory effects of miR-181a during inflammation [121].

*In vitro* and *in vivo* experiments demonstrate that long intergenic noncoding RNA-p21 (lincRNA-p21) induces tumor protein p53 (p53)-dependent microglial activation, leading to inflammation of the SN and death of dopaminergic neurons. Notably, miR-181a protects the cells against neuroinflammation and neurodegeneration by disrupting the p53-induced lincRNA-p21 signaling via the degradation of lincRNA-p21 and repression of protein kinase C delta (PKC-δ) [116].

Besides the loss of dopaminergic neurons in the SN, PD cases demonstrate the atrophy of gray matter in both cortical and subcortical brain regions. The accelerated gray matter atrophy in the striatum, including the PUT and caudate, occurs in the early to middle stages within the first five years of the disease. The rate of gray matter atrophy in the PUT and caudate slows in the late stage. The authors of this study recommend that nigrostriatal dopamine denervation may trigger striatal atrophy [131]. During normal aging, age-dependent accelerated loss of gray matter is observed bilaterally in the posterior PUT in men; however, women do not show evidence of accelerated gray matter loss in the PUT [122]. A meta-analysis demonstrates that the age-specific pooled male-to-female incidence ratio among individuals with PD increases progressively with age. These ratios markedly increase from 1.34 between 40 and 59 years to 1.46 between 60 and 79 years, and 1.93 in patients aged 80 years and over [132], revealing that men are more and more prone to develop PD with advancing age compared to women.

Altogether, increased expression of *MIR181A1HG* and possibly *MIR181A1* in the SN and PUT of elderly females with a normal aging brain may exert the neuroprotective effects against dopaminergic neuron loss and atrophy of gray matter in the PUT and thereby contribute to resistance against the PD disease in females aged 71 years and over during normal brain aging (Figures 4B, 4C). However, future research is required to determine the exact function of lncRNA *MIR181A1HG* and its intronic miRNAs miR-181a-1 and miR-181b-1 on neuron survival, dopaminergic transmission, and putamen gray matter plasticity.

Potential Mechanisms Involved in MIR181A1HG, the Canonical Wnt Signaling, GPC5, and IL33 That Resist Gray Matter Atrophy of the Putamen in Elderly Females

In addition, the GEO2R analysis of gene expression data generated using the GPL5175 microarray platform of the GSE46706 dataset demonstrated that females aged 71 years and over, compared to females aged 40-70 years, had differential expressions meeting criteria of adjusted *p* ≤ 0.05 and │log2FC│ ≥ 1.0 for six genes in the PUT (Table 1 and Figure 1). The analysis exhibited that EGF containing fibulin extracellular matrix protein 1 (BC098561, *EFEMP1*), interleukin 33 (BC047085, *IL33*), hydroxysteroid 17-beta dehydrogenase 6 (NM_003725, *HSD17B6*), solute carrier family 7 member 11 (NM_014331, *SLC7A11*), and glypican 5 (NM_004466, *GPC5*) were downregulated.

In addition, the GEO2R analysis of gene expression data produced using the same platform (GPL5175) showed that males aged 71 years and older, compared to males aged 40-70 years, had a differential expression meeting criteria of adjusted *p* ≤ 0.05 and │log2FC│ ≥ 1.0 for one gene in the PUT (Table 1, Figure 1). According to the result of this analysis, small nucleolar RNA, H/ACA box 14A (NR_002955, *SNORA14A*) was upregulated.

EFEMP1 (or Fibulin-3) functions as an extracellular matrix glycoprotein of the fibulin family [133]. Downregulation of EFEMP1 in the HC leads to a decline in spatial learning and memory in rats [134]. However, the role of the differential expression of *EFEMP1* in the PUT on brain aging remains unclear.

Functional enrichment analysis performed using the STRING database revealed that the complex interaction network of protein products of downregulated *IL33*, *EFEMP1*, *SLC7A11*, *GPC5*, and *HSD17B6* genes in the PUT of elderly females compared to females aged 40-70 years was mainly involved in biological processes related to interleukin-33-mediated signaling pathway, NLRP3 inflammasome complex assembly, positive regulation of T-helper 1 cell cytokine production, monocyte aggregation, interleukin-1 beta production, positive regulation of interleukin-5 production, positive regulation of T-helper 2 cell differentiation and cytokine production, positive regulation of interleukin-13 production, pyroptosis, interleukin-4 production, interleukin-6 production, negative regulation of DNA damage response-signal transduction by p53 class mediator, glial cell proliferation, astrocyte activation, and interferon-gamma production (Figure 2D and Appendix D).

IL33 acts as a cytokine showing a dual function in inflammation. Its physiological levels contribute to the anti-inflammatory response, whereas increased levels of IL33 can induce the pro-inflammatory signaling cascade, including cytokines, chemokines, and neurotoxic molecules in inflammatory cells such as astrocytes and microglia. IL33-induced cascade causes inflammation of neurons in the CNS, resulting in neurodegeneration and the impairment of cognitive function [125]. Oligodendrocytes are the predominant cell type, followed by neurons, astrocytes, and microglia, respectively, in the PUT of neurologically healthy control subjects [135]. Oligodendrocytes release IL33 after CNS injury, recruiting monocytes for recovery [136]. The expression of IL33 is upregulated in the midbrain and striatum of PD patients compared to the control group. IL33, reactive astrocytes, and mast cells that promote neuroinflammation are detected in the same areas, indicating that IL33 is released by glial cells and mast cells upon neuronal injury in PD [137].

SLC7A11 is a subunit of the cystine/glutamate antiporter system x_c_^-^. It mediates the uptake of cystine and biosynthesis of glutathione, thereby protecting the cells from oxidative stress and death via ferroptosis [138]. The overexpression of SLC7A11 inhibits ferroptosis during nerve damage triggered by lead exposure in HT22 mouse hippocampal neuronal cells [139]. The effect of SLC7A11 on ferroptosis remains unknown in the PUT.

Overexpression of GPC5 inhibits canonical wingless-type MMTV integration site family (Wnt)/β-catenin signaling to abrogate the interaction between Wnt3a and its cell surface receptor Frizzled8 by directly and competitively binding to the Wnt3a ligand at the surface of A549 lung adenocarcinoma cells [123,124]. Blocking the canonical Wnt/β-catenin signaling causes the loss of dopamine receptor clusters and dopaminergic synapse degeneration in the dorsal striatum, composed of the putamen and caudate nucleus, in the absence of axon retraction and neuron death in the secreted Wnt antagonist Dickkopf1 (Dkk1)-expressing adult transgenic mice, resulting in impairment in motor coordination. This study suggests that impaired canonical Wnt/β-catenin signaling triggers synaptic degeneration before neurodegeneration at early stages of neurodegenerative conditions, including PD [140,141]. The expression of the transcripts involving the canonical Wnt/β-catenin signaling, neurogenesis, synaptogenesis, and synaptic pruning is downregulated in the dorsal striatum of human postmortem cases, whereas the expression of the transcripts related to neuroinflammation is upregulated. The canonical Wnt/β-catenin pathway is age-dependent, dysregulated, suggesting that impaired canonical Wnt signaling may contribute to neuroinflammation and age-related deficiency in neurogenesis and synaptic plasticity in the striatum of elderly subjects with a normal aging brain [120].

In addition, the anti-inflammatory effects of miR-181a have been reported in monocytes and macrophages during inflammation [121]. The mature miR-181a-5p activates Wnt/β-catenin signaling by targeting Wnt inhibitory factor 1 (WIF1) in acute lymphoblastic leukemia (ALL) cells [117]. On the other hand, Wnt/β-catenin signaling can induce the expression of miR-181a in some cancer cells [118,119].

Overall, lncRNA *MIR181A1HG* and its intronic miRNA miR-181a-1 are likely to be involved in defending the striatum against neuroinflammation and synaptic degeneration through crosstalk with the canonical Wnt signaling in elderly females during normal brain aging (Figure 4C). GPC5 and IL33 could be targeted to inhibit synaptic loss and neuroinflammation, followed by neurodegeneration, leading to gray matter atrophy in the PUT, particularly in PD.

Potential Mechanisms Involved in Reduced SYT1 and Age-Dependent Cholesterol Depletion Associated With Cognitive Decline in the White Matter of Elderly Males

The GEO2R analysis of gene expression data generated using the GPL5175 microarray platform of the GSE46706 dataset revealed that males aged 71 years and over, compared to males aged 40-70 years, had differential expressions meeting criteria of adjusted *p* ≤ 0.05 and │log2FC│ ≥ 1.0 for three genes in the WM (Table 1 and Figure 1). The analysis exhibited that KCNIP4 intronic transcript 1 (NR_002813, *KCNIP4-IT1*; also known as *NCRNA00099*) and synaptotagmin 1 (BC058917, *SYT1*) were downregulated, whereas geminin coiled-coil domain containing (NM_001146686, *GMNC*; also known as *GEMC1*) (Figure 2E and Appendix E) was upregulated.

The synaptotagmin protein SYT1 is an integral membrane glycoprotein of synaptic vesicles and secretory granules in neurons and neuroendocrine cells, respectively. It functions as a primary Ca^2+^ sensor for vesicle fusion and exocytosis during neuronal transmission. It facilitates vesicle docking, priming, fusion, and exocytosis, supporting the efficient release of neurotransmitters upon calcium influx. SYT1 also has a regulatory function in endocytosis [142-144] (Figure 2F and Appendix F). During aging, there are prominent changes such as larger volume, thicker myelin, dysfunctional elongated mitochondria, a decline in the number of mitochondria, and decreased mitochondria-smooth ER contacts in the WM axons of mouse optic nerves. The WM axons in normal aging brains can adapt to age-related conditions. However, impairment in mitochondria, ER, and their interaction can cause dysregulated Ca^2+^ signaling, ER stress, deficient UPR, increased oxidative stress, and reduced adenosine 5′-triphosphate (ATP) levels in aging axons [145]. Cholesterol depletion abolishes SYT1-mediated synaptic vesicle fusion dependent on Ca^2+^ influx, indicating that cholesterol is required for Ca^2+^-dependent vesicular fusion [146].

Cholesterol is abundantly present in the myelin sheath wrapping around axons of the WM in the brain. The remaining is found in the plasma membranes or subcellular membranes. During aging, reduced cholesterol level or its varied localization within the plasma membrane leads to decreased release of neurotransmitters and impaired synaptic plasticity. Its age-related reduced levels also abolish the stability of SNARE complexes. In addition, the aging brain demonstrates decreased levels of plasma membrane receptors such as N-methyl-D-aspartate receptors (NMDARs) and α-amino-3-hydroxy-5-methyl-4-isoxazolepropionic acid receptors (AMPARs) that control calcium influx, leading to increased excitotoxic stress. There are also age-related abrogations in cholesterol biosynthesis, transport, and uptake in the brain [147].

The integrity of the WM is impaired during normal aging. The WM alterations are associated with cognitive decline. This study suggests that cortical network communication is weakened due to impaired WM integrity with advancing age, resulting in decreased performance in cognition [148].

**Figure 5:**
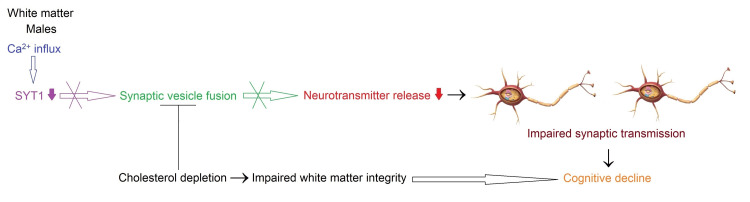
Potential mechanism underlying impairment in synaptic plasticity related to differential expression of SYT1 and varied cholesterol level in the WM of elderly males compared to middle-aged adult males during normal aging Image created by the author N.A. solely for the present study using the Microsoft Paint and Paint 3D programs (Microsoft Corp., USA) Upon Ca^2+^ influx, SYT1 plays a crucial role in synaptic vesicle fusion [142-144]. However, the downregulation of SYT1 and cholesterol depletion during normal brain aging can suppress synaptic vesicle fusion and decrease neurotransmitter release, causing impaired synaptic transmission and cognitive decline [146,147]. In addition, cholesterol depletion can trigger an impairment in the integrity of the white matter (WM), leading to cognitive decline [147,148].

Taken together, the downregulation of *SYT1* in the WM of elderly males may be related to cholesterol depletion in aging. The decrease in SYT1 expression and cholesterol level may contribute to impairment in synaptic plasticity, the release of neurotransmitters, and the WM integrity, resulting in the weakening of axonal connections between neurons in different gray matter regions (Figure 5).

Neurodegenerative diseases: general characteristics and cell death mechanisms

Programmed cell death pathways such as apoptosis, pyroptosis, ferroptosis, necroptosis, and autophagy-dependent cell death have been investigated as cellular mechanisms underlying neuronal cell loss in different neurodegenerative diseases (Table 2). These cell death pathways and the cross-talk between them can play crucial regulatory roles in neurodegeneration [149]. Disrupted proteostasis associated with the formation of misfolded protein aggregates is one of the primary hallmarks of neurodegenerative diseases [6]. Misfolded proteins in neurodegenerative disorders aggregate and form abnormal deposits such as β-amyloid plaques, tau tangles, and Lewy bodies. These toxic protein deposits activate the prodegenerative signaling pathways such as caspases/calpains, BCL2 apoptosis regulator (BCL2)/BCL2-associated X, apoptosis regulator (BAX), receptor-interacting serine/threonine kinase (RIPK), MAPK, sterile alpha, and TIR motif containing 1 (SARM1), and UPR, leading to neurodegeneration via some cell death mechanisms such as apoptosis, ferroptosis, necroptosis, parthanatos, aberrant autophagy, and autolysis [32,150].

**Table 2:** General characteristics and cell death mechanisms along with key players in neurodegenerative disorders AD: Alzheimer’s disease, ALS: amyotrophic lateral sclerosis, AMY: amygdala, BRS: brainstem, CAU: caudate, CER: cerebellum, CERH: cerebral hemispheres, CLS: claustrum, CNS: central nervous system, COR: cortex, CSTs: corticospinal tracts, dGM: deep gray matter, DCLs: deeper cortical layers, DLB: dementia with Lewy bodies, DMV: the dorsal motor nucleus of the vagus, EO: early-onset, EC: entorhinal cortex, f: familial, HC: hippocampus, HD: Huntington’s disease, HTHA: hypothalamus, LO: late-onset, m: mutant, MS: multiple sclerosis, ON: optic nerve, PLD: pallidum, PHG: parahippocampal gyrus, PD: Parkinson’s disease, PPN: pedunculopontine nucleus, PCG: posterior cingulate gyrus, PDD: PD with dementia, PFC: prefrontal cortex, PMC: primary motor cortex, PUT: putamen, s: sporadic, SPC: spinal cord, STR: striatum, SNpc: substantia nigra pars compacta, THA: thalamus, WM: white matter

References	Disorder	Gene variation	Aggregating protein/peptide	Affected CNS structure	Cell death mechanisms/key players
[151-165]	AD	EO: *APP*, *PSEN1*	Aβ, tau	PHG, HC, EC, AMY, PCG, PFC	Pyroptosis/caspase-1
		LO: *APOE*			Necroptosis/TNF-α
					Apoptosis/caspase-3, PUMA
					Impaired autophagy/Beclin 1
					Impaired mitophagy/PINK1 and PRKN
[166-181]	PD	fPD: *PARK2*, *VPS35*, *LRRK2*, *GBA*, *PINK1*, *SNCA*	PD: α-Syn, tau	SNpc, STR, PPN, DMV, AMY, COR	Apoptosis/caspase-8, ROCK1, DRP1
		sPD: *INPP5F*, *MAPT*, *KANSL1*, *SNCA*, *LRRK2*	PDD: α-Syn, tau, Aβ		Pyroptosis/caspase-1
					Impaired autophagy/SNAP29, TFEB, Beclin 1
					Impaired mitophagy/MIRO1, PINK1, parkin (PARK2)
[182-195]	DLB	sDLB: *APOE*, *GBA*, *SNCA*, *CHMP2B*, *SQSTM1*, *PARK2*, *EIF4G1*, *GIGYF2*	α-Syn, tau, Aβ	EC, HC, AMY, PHG	Apoptosis/caspase-3
		fDLB: *VPS13C*, *APOE*			Impaired autophagy/mTOR, ATG7
[196-207]	HD	*HTT* (CAG repeat expansion)	mHTT	STR, DCLs, HC, CER, AMY, HTHA, THA	Apoptosis/caspases-1 and 3
					Impaired autophagy/Beclin 1
[208-220]	ALS	sALS: *C9ORF72*, *NEK1*, *SOD1*, *DCTN1*, *TARDBP*, *OPTN*, *KIF5A*, *TBK1*, *SETX*, *ERBB4*, *SQSTM1*, *MATR3*, *FUS*	sALS: TDP-43, ATXN2, UBQLN2, OPTN, FUS, C9ORF72, SOD1	PMC, BRS, SPC, CSTs	C9ORF72 ALS: apoptosis/ p53, caspase-3
		fALS: *C9ORF72*, *SOD1*, *TARDBP*, *OPTN*, *KIF5A*	fALS: SOD1, TDP-43, FUS, C9ORF72		SOD1 fALS: apoptosis/caspases-1 and 3
					OPTN ALS: impaired autophagy/TBK1
					Apoptosis/caspase-3
					Impaired mitophagy/OPTN, TBK1
[221-228]	MS	*DRB1*, *DQB1*, *DRB5*	Tau, APP, RIPK1, RIPK3	ON, BRS, CER, SPC, CERH, dGM, THA, CAU, PUT, HTHA, AMY, SN, PLD, CLS, WM	Necroptosis/TNF-α, RIPK1, RIPK3, FADD, MLKL
					Impaired autophagy/apoptosis/caspase-3
					Apoptosis/necroptosis/RAB32

Alzheimer’s Disease

Volumetric atrophy occurs in the parahippocampal gyrus, entorhinal cortex, HC, amygdala, and posterior cingulate gyrus in the initial stages of AD. The reduction of these sites continues in the late stages. In the later stages of AD, the PFC shows volumetric reduction as well [151]. The Aβ and tau are the main aggregating molecules of AD [152]. Amyloid deposition initially forms in the temporobasal and frontomedial regions and later progresses to the remaining associative neocortex, primary sensory-motor cortex, medial temporal lobe, and finally striatum in AD [153]. Tau accumulation is initially observed in the EC and Brodmann area 35 in AD. Tau pathology later progresses into the HC, Brodmann area 36, and parahippocampal cortex. Neocortical temporal and parietal brain areas are affected by tau pathology at the final stages of the disease [154,155].

Aβ peptides are derived from APP, a transmembrane protein, through the proteolytic action of BACE1 and the γ-secretase enzyme complex containing presenilin 1 (PSEN1, also known as PS1). Rare mutations of *APP* and *PSEN1* in familial early-onset AD lead to the accumulation of pathological Aβ species, particularly Aβ_40_ and Aβ_42_ in the brain. Environmental risk factors and apolipoprotein E4 (ApoE4) encoded by the ε4 allele of the *APOE* gene can contribute to toxic Aβ species-induced tau pathology in late-onset AD. Increased levels of Aβ_42_, which is more hydrophobic than Aβ_40_, cause the formation of the amyloid fibrillar structure constituting senile plaques [156,157]. Specifically, Aβ_42_ interacts with glycogen synthase kinase 3α (GSK3α), which hyperphosphorylates tau protein [158]. 3-repeat (3R) and 4-repeat (4R) tau isoforms are hyperphosphorylated and included in pathological inclusions in AD. These inclusions exist as NFTs in neuronal cell bodies, whereas tau inclusions are observed as threads in dendrites and axons. Tau toxicity leads to cell cycle re-entry and synaptic dysfunction in neurons. Consequently, neuronal loss causes memory impairment and cognitive decline in AD [156,157].

The deficiency of programmed cell death 1 (PD1) in the APP/PS1 mouse model of AD increases the formation of Aβ plaques in cortical and hippocampal areas. Because microglial cells are incapable of efficient uptake of Aβ due to deficient PD1. Therefore, increased deposition of Aβ plaques leads to chronic neuroinflammation in AD [159]. Microglial uptake and degradation of Aβ is reduced by producing pro-inflammatory cytokines in response to Aβ deposition upon progression in aging APP/PS1 transgenic mouse, resulting in Aβ accumulation [160]. Aβ_1-42_ treatment promotes pyroptotic neuronal death dependent on caspase-1 through upregulation of the expression of NLR family pyrin domain containing 1 (NLRP1) in cultured rat cortical neurons and APP/PS1 transgenic mouse model. The activation of NLRP1 inflammasome in response to Aβ accumulation promotes caspase-1-dependent pyroptotic neuron death, causing the cognitive decline in AD [161]. In addition, microglial cells mediate necroptotic neuronal death through the release of TNF-α in response to Aβ oligomer treatment, resulting in neurodegeneration and memory deficits in AD [162].

In an apoE(-/-) hyperlipidemic mouse model, hippocampal lipid accumulation results in caspase-3-mediated apoptotic death of the CA3 neurons in the HC. High-fat diet upregulates the expression of proprotein convertase subtilisin/kexin type 9 (PCSK9) and BACE1 during apoptosis in the HC. Upregulation of BACE1 triggers the formation of Aβ plaques, leading to neuronal apoptosis. This study suggests that Aβ plaques may be involved in the caspase-3-dependent apoptotic death of hippocampal neurons in AD [66]. The oligomeric Aβ treatment simultaneously induces apoptosis and autophagy-dependent cell death in cultured neuronal cells and the brain of 5xFAD mice. Upon Aβ toxicity, autophagic Beclin 1 and apoptotic p53-upregulated modulator of apoptosis (PUMA) proteins interact with each other and simultaneously induce aberrant autophagy and apoptosis. They impair autophagy flux and promote neuronal apoptosis [163].

The intracellular accumulation of tau in HEK293T cells and rat hippocampal neurons suppresses autophagosome formation through activation of the mammalian target of rapamycin kinase complex 1 (mTORC1) pathway, causing impairment in the autophagy process [164]. The overexpression of tau protein is associated with an increase in mitochondrial markers cytochrome c oxidase subunit IV (COXIV) and translocase of outer mitochondrial membrane 20 (TOMM20) levels in postmortem tissues from AD brains, a transgenic mouse model of AD, and primary hippocampal neurons. *In vitro* and *in vivo* experiments exhibit that intracellular tau accumulation causes mitophagy deficiency by reducing the levels of PINK1 and PRKN (or parkin) in mitochondria [165].

Parkinson’s Disease

PD is characterized by the loss of dopaminergic neurons in the SNpc and striatal dopamine terminals, causing motor impairments. The neuronal loss also occurs to a lesser extent in the pedunculopontine nucleus, the dorsal motor nucleus of the vagus, the amygdala, and the cortex [166]. Presynaptic neuronal protein α-Syn is primarily an aggregated protein inside the Lewy bodies in PD. But tau has been reported to colocalize with α-Syn in the Lewy bodies in postmortem brain tissues of PD patients [167]. α-Syn, Aβ, and tau pathologies are more frequently observed in PD with dementia (PDD) than in non-demented PD [168]. Leucine-rich repeat kinase 2 (*LRRK2*), *PARK2*, VPS35 retromer complex component (*VPS35*), and synuclein alpha (*SNCA*) gene mutations are most commonly detected in familial PD (fPD) cases in the world. Some mutations of *PARK2*, *LRRK2*, glucosylceramidase beta (*GBA*), *PINK1*, and *SNCA* are among the top genetic variants increasing the risk for fPD, whereas inositol polyphosphate-5-phosphatase F (*INPP5F*), microtubule-associated protein tau (*MAPT*), and KAT8 regulatory NSL complex subunit 1 (*KANSL1*) with single-nucleotide polymorphisms (SNPs) constitute the top three polymorphic genes enhancing the risk for developing sporadic PD (sPD) [169]. Some mutations of *SNCA* and *LRRK2* are significant risk factors for sPD as well [170].

Caspase-8 is more activated in dopaminergic neurons of SNpc in postmortem tissues from PD patients compared to the control group. The Parkinsonian toxin 1-methyl-4-phenyl-1,2,3,6-tetrahydropyridine (MPTP)-induced mouse model of PD demonstrates that caspase-8-dependent apoptotic dopaminergic neuron death occurs in the SNpc of mice [171]. *In vitro* and *in vivo* studies demonstrate that Rho-associated coiled-coil-containing protein kinase 1 (ROCK1) promotes aberrant mitochondrial fission and apoptosis dependent on dynamin-related protein 1 (DRP1) activation in 1-methyl-4-phenylpyridinium (MPP^+^)-treated PC12 cells, a cell culture model of PD. Inhibition of ROCK1 results in the repression of apoptosis of dopaminergic neurons in the MPTP-induced mouse model of PD [172]. α-Syn-induced mitochondrial dysfunction leads to neurodegeneration, particularly via caspase-1 activation in dopaminergic Lund human mesencephalic (LUHMES) neurons [173]. The caspase-1-dependent signaling pathway is associated with pyroptosis in the MPTP-induced mouse model of PD. Pyroptosis has been observed as widespread in PD mice [174]. Activated inflammasome complex, including caspase-1, triggers the aggregation of α-Syn in the neuronal cell model of PD. Caspase-1 generates highly aggregation-prone forms of α-Syn by directly cleaving it, resulting in toxicity in PD neurons [175].

Overexpression of α-Syn reduces the turnover of autophagy to impair the fusion between the autophagosome and lysosome by decreasing the expression of synaptosome-associated protein 29 (SNAP29) in cultured human dopaminergic neurons. Upon the inhibition of autophagy, the elevation of the abundance of oligomeric α-Syn is highly likely to induce neuronal cell death. The expression of SNAP29 is decreased at an early stage of disease in SNpc dopaminergic neurons of postmortem brain tissues of patients with Lewy body pathology. A compensatory mechanism for dysfunctional autophagy facilitates the fusion of autophagosomes with the plasma membrane, releasing extracellular vesicles into the extracellular space [176]. This study suggests that the compensatory release of extracellular vesicles, including α-Syn aggregates from cells, may contribute to the spreading of α-Syn pathology in PD brain. In line with the above results, a rat model demonstrates that increased levels of α-Syn suppress the autophagy-lysosome pathway and cause cytoplasmic retention of transcription factor EB (TFEB) that regulates this pathway. Overexpression of TFEB or Beclin 1 blocks α-Syn pathology and thereby prevents degeneration of dopaminergic neurons. In addition, the nuclear expression of TFEB declines in postmortem midbrain dopaminergic neurons of PD patients [177]. Mitochondrial Rho GTPase 1 (MIRO1) expression increases in the SN dopaminergic neurons of postmortem brain tissues of PD patients. MIRO1 protein is located on the outer mitochondrial membrane. It mediates the motility of mitochondria. During mitophagy, decreasing levels of MIRO1 contribute to the clearance of damaged mitochondria. The accumulation of α-Syn upregulates MIRO1, leading to delayed mitophagy and neurodegeneration [178,179]. Exogenous α-Syn treatment decreases the mitochondrial level of parkin and impairs the mitophagy process in neuronal PC12 cells, leading to the accumulation of defective mitochondria [180]. Vulnerability to the aggregation of α-Syn and dopaminergic neurodegeneration is elevated in the *PINK1*-knockout rat model of PD [181].

Dementia With Lewy Bodies

DLB is associated with remarkable symptoms, including cognitive fluctuations, spontaneous extrapyramidal motor characteristics, visual hallucinations, and rapid eye movement sleep behavior disorder [182]. PDD cases show similar clinical features to DLB individuals. However, they can develop dementia at least one year after diagnosis of PD, whereas dementia occurs before or concurrently with the onset of Parkinsonism in DLB [183]. DLB is characterized by moderate atrophic areas, particularly in the HC, EC, amygdala, and right parahippocampal gyrus [184]. DLB has α-Syn Lewy body pathology in limbic and cortical areas. PDD patients demonstrate α-Syn Lewy body pathology mostly in medial temporal lobe regions, but later progress to neocortical and subcortical areas. A group of PDD cases also harbors Aβ plaques and phosphorylated tau tangles in the cortex [185]. The accumulation of tau is observed primarily in temporal and parietal regions in DLB. Aβ burden can be detected in the neocortex of DLB patients [186].

The *APOE* E4 allele identified in AD has been detected in sporadic DLB as well. *GBA* and *SNCA* variations found in PD have also been identified in sporadic DLB [187,188]. A study of whole-exome sequencing identified mutations of charged multivesicular body protein 2B (*CHMP2B*), *SQSTM1*, *PARK2*, eukaryotic translation initiation factor 4 gamma 1 (*EIF4G1*), and GRB10 interacting GYF protein 2 (*GIGYF2*) in patients with sporadic DLB [189]. On the other hand, the *APOE* E4 allele and the mutations of vacuolar protein sorting 13 homolog C (*VPS13C*) have been identified in familial DLB [190,191].

The brain homogenates from DLB cases demonstrate that small α-Syn aggregates accumulate at presynaptic terminals and cause severe loss of dendritic spines at the postsynaptic area [192]. Presynaptic terminal density significantly decreases in both cortical regions and the SN of living DLB and PDD cases, whereas it is significantly reduced only in the SN of non-demented PD patients. This study suggests that progressive cognitive impairment in dementia may be related to reduced synaptic density primarily in cortical areas [193]. A study demonstrates that α-Syn aggregates can be transmitted cell-to-cell via endocytosis. In this way, α-Syn deposits form Lewy-like inclusions in acceptor neurons. Particularly, lysosomal system failure facilitates the accumulation of α-Syn aggregates and the formation of Lewy-like inclusions in acceptor neurons. These acceptor cells expose caspase-3-dependent apoptosis upon transmission and propagation of α-Syn [194]. This study suggests that presynaptic α-Syn accumulation may trigger cell-to-cell transmission of aggregates, leading to apoptotic death of acceptor neurons in Lewy body diseases. Defective autophagy induced by upregulated mTOR and downregulated ATG7 may lead to neurodegeneration due to the accumulation of α-Syn in postmortem tissues of DLB cases and α-Syn transgenic mice [195].

Huntington’s Disease

HD is a monogenic neurodegenerative disorder that is inherited in an autosomal dominant manner. HD definitively occurs in individuals who inherited an allele with a cytosine-adenine-guanine (CAG) trinucleotide repeat number greater than 39 in the *HTT* gene located on chromosome 4p16.3, leading to disease at full penetrance. Healthy individuals in the general population have a CAG repeat number between 6 and 35, but a repeat range of 27-35 harbors an expansion risk for the next generation, generally through paternal transmission. A mutant allele with a CAG repeat number between 36 and 39 is associated with incomplete penetrance, causing slower disease progression [196-198].

Neurodegeneration triggered by mutant HTT in HD selectively occurs in the striatum and deeper layers of the cortex in the early stages of the disease. Atrophy is also observed in other brain areas, including the HC, CER, amygdala, hypothalamus, and some nuclei in the thalamus in advanced stages [199]. The pathological polyglutamine (polyQ) expansion involving the HTT protein in HD causes motor, psychiatric, and cognitive impairments [200].

The Q150 knock-in mouse model of HD demonstrates that NH2-terminal mutant HTT fragments generate nuclear HTT inclusions that are prone to aggregation in neurons. Decreased proteasome activity in the aging brain contributes to the toxic aggregation and nuclear accumulation of mutant HTT fragments due to the inability to clear the degraded fragments. These findings suggest that proteasome failure may lead to the progressive accumulation of mutant HTT fragments in late-onset HD [201]. A *Drosophila *model of polyQ expansion demonstrates that long polyQ peptides exhibit intrinsic cytotoxic effects dependent on cell type, leading to neuronal degeneration [202]. The increased copper content in the brain facilitates HTT-polyQ aggregation by directly binding to exon 1 in a *Drosophila* model of HD. The copper binding and the toxicity of HTT exon 1 with expanded polyQ affect the progression of HD [203]. The polyQ-expanded HTT protein abrogates the turnover of ER proteins in yeast cells and neuron-like PC12 cells. Impairment in ER homeostasis induces ER stress, leading to the activation of the UPR. However, the heat-shock protein response does not occur. Therefore, misfolded HTT protein with polyQ expansion cannot be exposed to clearance by the heat-shock protein response system in neuronal cells [204]. 

Intranuclear mutant HTT induces apoptosis through the activation of caspases 1 and 3 in PC12 cells transfected with polyQ (150 Q)-expanded HTT protein [205]. A study reveals that wild-type HTT protein is required for normal selective autophagy, including mitophagy and nucleophagy, in *Drosophila* and mice [206]. *In vitro* and *in vivo* experiments suggest that accumulated polyQ-expanded mutant HTT selectively inhibits Beclin 1-mediated long-lived protein turnover of the autophagic process. The authors recommend that late-onset HD may progress through the accumulation of mutant HTT triggered by the suppression of Beclin 1-dependent autophagy upon the decrease of Beclin 1 in the aging brain [207].

Amyotrophic Lateral Sclerosis

ALS is a neurodegenerative disease characterized by progressive loss of upper and lower motor neurons. ALS commonly occurs in the sporadic form; however, approximately 5-10% of ALS cases have a family history [208]. The areas affected by neurodegeneration in ALS are the primary motor cortex, brainstem, spinal cord, and corticospinal tracts [209]. Misfolded protein aggregates in ALS generate intracellular insoluble inclusions. These inclusions can be found in the brain stem, spinal cord, FC, temporal cortex, thalamus, HC, and CER [210]. The misfolded and aggregated proteins identified in sporadic ALS include TDP-43, ATXN2, ubiquilin 2 (UBQLN2), optineurin (OPTN), FUS RNA-binding protein (FUS), C9orf72-SMCR8 complex subunit (C9ORF72), and SOD1. SOD1, TDP-43, FUS, and C9ORF72 aggregates have also been detected in familial ALS [211-214].

*C9ORF72*, NIMA-related kinase 1 (*NEK1*), *SOD1*, dynactin subunit 1 (*DCTN1*), TAR DNA binding protein (*TARDBP*), *OPTN*, kinesin family member 5A (*KIF5A*), TANK binding kinase 1 (*TBK1*), senataxin (*SETX*), Erb-B2 receptor tyrosine kinase 4 (*ERBB4*), *SQSTM1*, matrin 3 (*MATR3*), and *FUS* are among the most commonly mutated genes in sporadic ALS. Familial ALS is more frequently associated with mutations of *C9ORF72*, *SOD1*, *TARDBP*, *OPTN*, and *KIF5A* [215].

The mouse, fly, and human models of *C9ORF72* repeat expansion demonstrate that p53 mediates neurodegeneration induced by *C9ORF72* mutation. Induced pluripotent stem cell (iPSC)-derived motor neurons of *C9ORF72* ALS patients undergo caspase-3-dependent apoptosis induced by p53 in response to DNA damage [216]. *In vitro* and *in vivo* experiments demonstrate that motor neuron death depends on sequential activation of caspase-1 and caspase-3 in familial ALS with mutant *SOD1*. Increased oxidative stress caused by mutant SOD1 leads to activation of caspase-1, resulting in caspase-3-dependent axonal loss and apoptotic cell death of motor neurons in the spinal cord and cortex [217]. A mutant TDP-43 causing ALS leads to the death of motor neurons in mice [218].

An insertional mutation of *OPTN* associated with ALS causes the activation of TBK1, resulting in deficient autophagy and thereby accumulation of aggregates in motor neuron-like cell line NSC-34. The impairment of autophagy triggers ER stress, followed by cell death dependent on caspase-3 [219]. The mutations of *OPTN* or *TBK1* associated with ALS impede the efficient formation of the autophagosome, causing inhibition of selective mitophagy [220].

Multiple Sclerosis

MS is characterized by chronic inflammation, demyelination, and neurodegeneration in the CNS. Most MS patients experience their initial attack during the clinically isolated syndrome (CIS) phase. In the CIS phase, inflammatory demyelinating lesions can be observed in the optic nerve, brainstem, CER, spinal cord, and cerebral hemispheres [221]. Demyelinating lesions of deep gray matter in MS are detected in the thalamus, caudate, PUT, hypothalamus, amygdala, SN, pallidum, and claustrum. Inflammation and axonal damage/loss usually occur in the WM. The loss of neuronal and glial cells is more frequently observed in the caudate nucleus and thalamic mediodorsal nucleus in demyelinated deep gray matter compared to non-demyelinated deep gray matter [222].

Insoluble hyperphosphorylated tau accumulates at chronic WM lesions in postmortem cerebral tissues of secondary progressive MS patients. Moreover, axonal APP is detected abundantly in active lesions [223]. Multiple allelic variants of human leukocyte antigen (HLA) genes *DRB1*, *DQB1*, and *DRB5* in the MHC class II region are associated with an increased risk of developing MS [224].

*In vitro* and *in vivo* experiments demonstrate that TNF-α induces the loss of oligodendrocytes and inflammation via RIPK1/RIPK3-dependent necroptosis in MS. This study also demonstrates that RIPK1 and RIPK3 form insoluble aggregates in postmortem cortical lesions of human MS patients [225]. An experimental autoimmune encephalomyelitis (EAE) mouse model of MS demonstrates that defective autophagy causes the activation of the caspase-3-mediated apoptotic pathway, resulting in neuronal damage in the spinal cord of EAE mice [226]. Postmortem cortical gray matter tissues of secondary progressive MS (SPMS) individuals exhibit that cell death of cortical neurons occurs through necroptosis dependent on the TNF/TNFR1 signaling pathway involving the activation of Fas-associated via death domain (FADD), RIPK1, RIPK3, and mixed lineage kinase domain-like pseudokinase (MLKL) [227]. Postmortem deep WM tissues of MS cases, EAE mice tissues, human primary neurons, and neuronal cells reveal that ER stress causes upregulation of UPR-targeted RAB32, member RAS oncogene family (RAB32) during the progression of MS. Overexpression of RAB32 leads to mitochondrial dysfunction and activation of apoptosis and necroptosis, resulting in neuronal cell death in the MS brain [228], suggesting that defective mitophagy may cause activation of apoptosis/necroptosis pathway in progressive MS brain.

Gender-specific differential gene expressions and related molecular and cellular mechanisms in the entorhinal cortex and hippocampus in AD

A GEO dataset (GSE118553) includes microarray transcriptome data generated using the Illumina beadArray platform of postmortem tissue samples dissected from four brain regions, i.e., EC, TC, FC, and CER, of 52 AD patients and 27 controls without dementia and neurodegeneration [45,48,229].

Differential Gene Expressions and Associated Potential Mechanisms Identified in the Entorhinal Cortex of Females With AD

Using the GEO2R tool, the analysis of microarray gene expression data of the GSE118553 dataset demonstrated that the EC downregulated the expression of 16 genes [myelin basic protein (4155, *MBP*), progestin and adipoQ receptor family member 6 (79957, *PAQR6*), tumor protein p53 inducible nuclear protein 2 (58476, *TP53INP2*), myelin associated oligodendrocyte basic protein (4336, *MOBP*), solute carrier family 4 member 8 (9498, *SLC4A8*), potassium sodium-activated channel subfamily T member 1 (57582, *KCNT1*), brain enriched myelin associated protein 1 (8537, *BCAS1*), extracellular leucine rich repeat and fibronectin type III domain containing 2 (114794, *ELFN2*), stathmin 2 (11075, *STMN2*), pannexin 2 (56666, *PANX2*), nucleophosmin/nucleoplasmin 2 (10361, *NPM2*), adenylate cyclase 2 (108, *ADCY2*), carnosine synthase 1 (57571, *CARNS1*), transgelin 3 (29114, *TAGLN3*), SV2 related protein (55530, *SVOP*), and BCL11 transcription factor A (53335, *BCL11A*)] in female AD patients (*n* = 21, age range: 61-98 years) compared to female controls (*n* = 5, age range: 51-92 years) (Data Appendix B, Data S21). The selected options of GEO2R analysis are Benjamini & Hochberg False Discovery Rate for applying adjustment to the *p*-values, auto-detect for applying log transformation to the data, yes for applying limma precision weights (vooma), and yes for force normalization. The DEGs were determined based on the criteria of adjusted *p* ≤ 0.05 and │log2FC│ ≥ 1.0.

Functional enrichment analysis showed that the complex interaction network of protein products of downregulated *BCAS1*, *MOBP*, *MBP*, and *CARNS1* genes in the EC of female AD patients compared to female controls was mainly involved in biological processes related to carnosine metabolic process, histidine catabolic process, positive regulation of myelination, CNS myelination, positive regulation of gliogenesis, glial cell differentiation, and axon development (Appendix G) [53,54].

**Figure 6:**
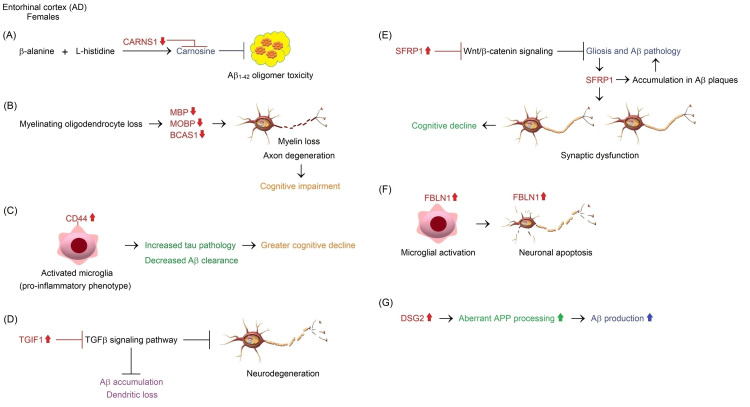
Potential molecular and cellular mechanisms related to some differential gene expressions identified in the EC of female AD patients compared to female controls Image created by the author N.A. solely for the present study using the Microsoft Paint and Paint 3D programs (Microsoft Corp., USA) (A) Downregulation of the CARNS1 enzyme causes the repression of carnosine production and thereby increases neuronal vulnerability to the toxic effects of Aβ1-42 oligomers in the EC of female AD brains [230-233]. (B) The loss of myelinating oligodendrocytes causes a decrease in the expression of myelin proteins MBP, MOBP, and BCAS1, resulting in myelin loss and axon degeneration. Demyelination and axonal degeneration of certain EC neurons may contribute to early cognitive impairment [234-240]. (C) Upregulation of CD44 expression on the surface of activated microglial cells, along with the acquisition of a pro-inflammatory phenotype, increases tau pathology and decreases the efficiency of Aβ clearance, leading to greater cognitive decline [241]. (D) Upregulation of TGIF1 represses the TGFβ signaling pathway and thereby increases Aβ accumulation, dendritic loss, and neurodegeneration [242,243]. (E) Upregulation of SFRP1 suppresses the canonical Wnt/β-catenin signaling pathway, enhancing gliosis and Aβ pathology in AD. Glial cell-derived SFRP1 localizes to the synapses, causing synaptic dysfunction and cognitive decline. In addition, SFRP1 binds to Aβ and accumulates in the amyloid plaques, increasing Aβ pathology. The complex interplay between SFRP1 and the canonical Wnt signaling pathway may play critical roles in the pathogenesis of female AD in the EC [244-246]. (F) Upregulation of FBLN1 expression in microglia and neurons may contribute to neuronal cell death through microglial activation and the induction of apoptosis, respectively [247]. (G) Upregulation of DSG2 increases aberrant APP processing and Aβ production [248]. EC: entorhinal cortex, AD: Alzheimer’s disease, APP: amyloid precursor protein

Carnosine, a histidine-containing dipeptide, is synthesized from β-alanine and L-histidine amino acids through the action of ATP-dependent CARNS1 enzyme [230-232]. Carnosine inhibits the toxic effects of Aβ_1-42 _in mixed cell cultures treated with Aβ_1-42_ oligomers of rat cortical neurons, astrocytes, and microglia [233] (Figure 6A).

Mature oligodendrocytes produce myelin and myelin proteins, including the MBP, in the CNS. The plasma membrane of the oligodendrocytes generates the compact myelin sheath that wraps tightly around the axons. Oligodendrocytes and myelin sheath facilitate the fast transmission of action potentials. They also ensure metabolic support to the axons [234]. The q-space myelin map imaging demonstrates that myelin density significantly decreases in the left HC, right precuneus, left insula, and bilateral anterior cingulate areas of AD patients compared to healthy controls [249]. In addition, the loss of oligodendrocytes and myelin is detected in the EC of AD patients. In line with the EC data of AD cases from the GSE118553 dataset, the expression of MBP significantly declines in postmortem EC tissues of cases with AD pathology compared to the control group [235] (Figure 6B).

The oligodendrocyte subpopulations o3 and o5 upregulate the expression of myelination-related genes, including *MOBP*, indicating that these subpopulations are mature myelinating oligodendrocytes. The o3 and o5 subpopulations are downregulated in AD, showing that the number of myelin-producing mature oligodendrocytes declines in AD. The expression of *MOBP* is downregulated in postmortem PFC tissues of AD cases compared to the normal control group [236] (Figure 6B).

BCAS1 is a myelin-related protein. It is expressed specifically in mature oligodendrocytes. Notably, the loss of *BCAS1* triggers hypomyelination and upregulates the expression of the inflammation-related genes such as S100 calcium-binding protein A8 (*S100a8*), S100 calcium-binding protein A9 (*S100a9*), and growth hormone (*GH*) in the brain [237] (Figure 6B).

AD patients demonstrate a substantial decrease of 48% in the mean total neuron number of the EC compared to non-demented controls. There are significant decreases in neuron numbers of all layers of the EC in AD; however, layers II and IV show greater declines than other layers. The decreases start in very mild AD and continue in severe AD. The neuronal loss is positively correlated with the burden of NFTs and neuritic plaques, but not that of amyloid plaques, in the EC. The results exhibit that the neuropathological change and the death of the HC and EC neurons contribute to cognitive decline and memory loss in AD [250,251].

Using the GEO2R tool, the analysis of microarray gene expression data of the GSE118553 dataset revealed that the EC upregulated the expression of 29 genes [ANTXR cell adhesion molecule 1 (84168, *ANTXR1*), TGFB-induced factor homeobox 1 (7050, *TGIF1*), carnitine palmitoyltransferase 1A (1374, *CPT1A*), family with sequence similarity 84 member B (157638, *FAM84B*), IQ motif containing GTPase activating protein 2 (10788, *IQGAP2*), dynein axonemal light intermediate chain 1 (7802, *DNALI1*), RNA binding motif protein 47 (54502, *RBM47*), CLN5 intracellular trafficking protein (1203, *CLN5*), EGF containing fibulin extracellular matrix protein 1 (2202, *EFEMP1*), solute carrier family 16 member 10 (117247, *SLC16A10*), TNF receptor superfamily member 11b (4982, *TNFRSF11B*), enoyl-CoA hydratase domain containing 3 (79746, *ECHDC3*), small nucleolar RNA, C/D box 71 (692111, *SNORD71*), ETS homologous factor (26298, *EHF*), matrilin 2 (4147, *MATN2*), desmoglein 2 (1829, *DSG2*), Ras association domain family member 9 (9182, *RASSF9*), C1q and TNF related 5 (114902, *C1QTNF5*), solute carrier family 6 member 15 (55117, *SLC6A15*), solute carrier family 39 member 12 (221074, *SLC39A12*), syntrophin beta 1 (6641, *SNTB1*), fibulin 1 (2192, *FBLN1*), hydroxyacyl-CoA dehydrogenase (3033, *HADH*), calmodulin like 4 (91860, *CALML4*), solute carrier family 15 member 4 (121260, *SLC15A4*), integrin subunit beta 4 (3691, *ITGB4*), secreted frizzled related protein 1 (6422, *SFRP1*), WD repeat-containing protein 78 (79819, *WDR78*), and CD44 molecule (Indian blood group) (960, *CD44*)] in female AD patients (*n* = 21, age range: 61-98 years) compared to female controls (*n* = 5, age range: 51-92 years) (Data Appendix B, Data S21). The DEGs were determined based on the criteria of adjusted *p* ≤ 0.05 and │log2FC│ ≥ 1.0.

Functional enrichment analysis demonstrated that the complex interaction network of protein products of upregulated *FBLN1*, *EFEMP1*, and *ANTXR1* genes in the EC of female AD patients compared to female controls was mainly involved in biological processes related to positive regulation of substrate-dependent cell migration, negative regulation of transforming growth factor beta (TGFβ) production, toxin transport, and regulation of ERK1 and ERK2 cascade (Appendix H) [53,54].

The cell surface markers CD44 and leukocyte common antigen (CD45), and potassium voltage-gated channel subfamily A member 3 (KCNA3, also known as Kv1.3) are expressed at a higher level in the activated microglial cells with a pro-inflammatory phenotype than in the anti-inflammatory microglial cells in AD mouse models. The inhibition of the pro-inflammatory microglial phenotype, along with the promotion of the anti-inflammatory microglial phenotype, ensures an efficient Aβ clearance by enhancing phagocytic activity in AD models. The pro-inflammatory microglial proteins, including CD44, are upregulated in the preclinical stages of AD. The expression of these proteins is positively correlated with tau pathology, resulting in cognitive decline [241] (Figure 6C).

An hAPP transgenic mouse model of AD demonstrates that the suppression of neuronal TGFβ signaling leads to increased Aβ accumulation and dendritic loss in neurons. In addition, repressing TGFβ signaling causes age-dependent neurodegeneration in mice. The protein levels of TGFβ type II receptor (TβRII) are downregulated in the mid-frontal cortical gray matter from postmortem AD brains, indicating the impairment in neuronal TGFβ signaling in AD brains [242]. TGIF1 inhibits the TGFβ signaling pathway through the recruitment of a histone deacetylase (HDAC) corepressor complex in the nucleus [243] (Figure 6D). 

The focal lesion of EC formed by the injection of kainic acid in rats causes neuronal cell death in the EC and the hippocampal CA3 region. Upon the formation of the EC lesion, reactive astrocytes are observed primarily in the HC. The EC damage induces upregulation of cyclin D1 in the G1 phase and cyclin B in the G2-M transition through the activation of ERK1/2 in hippocampal CA3 pyramidal neurons, promoting cell cycle re-entry followed by the death of postmitotic neurons [252]. The expression of cyclin D1, CDK6, cyclin B, and CDK2 increases in the EC and DG after an excitotoxic lesion generated by the injection of kainic acid in the DG of rats. The protein expression of apoptotic BAX is also elevated in the DG and EC after the formation of the lesion in rats. The AD-related phosphorylated tau and APP proteins show a progressive increase in the EC and DG after the DG lesion. This study suggests that cell cycle re-entry via upregulation of cell cycle regulators increases tau and APP pathology and later promotes apoptotic cell death, resulting in neurodegeneration in the EC and DG areas of AD brains upon synaptic damage caused by the DG lesion [253].

*SFRP1* is expressed in microglia, astrocytes, and choroid plexus in APP/PS1 mice. Its expression is increased in the EC and FC of human AD patients, compared to the control group. It accumulates in the amyloid plaques and binds to Aβ. Glial cell-derived SFRP1 is localized to the synapses of the APP/PS1 mouse brain, leading to synaptic dysfunction and cognitive decline [244]. The canonical Wnt/β-catenin signaling activity decreases in the EC of AD mice compared to wild-type mice. The decrease in the Wnt/β-catenin signaling activity precedes gliosis and Aβ pathology [245]. SFRP1 inhibits the activity of the canonical Wnt signaling [246] (Figure 6E).

Differential Gene Expressions and Associated Potential Mechanisms Identified in the Entorhinal Cortex of Males With AD

Using the GEO2R tool, the analysis of microarray gene expression data of the GSE118553 dataset demonstrated that the expression of 13 genes [growth arrest specific 7 (8522, *GAS7*), DIRAS family GTPase 2 (54769, *DIRAS2*), tumor protein p53 inducible nuclear protein 2 (58476, *TP53INP2*), complexin 1 (10815, *CPLX1*), myelin associated oligodendrocyte basic protein (4336, *MOBP*), myelin basic protein (4155, *MBP*), rabphilin 3A (22895, *RPH3A*), septin 4 (5414, *SEPT4*), aspartate beta-hydroxylase domain containing 1 (253982, *ASPHD1*), engulfment and cell motility 1 (9844, *ELMO1*), olfactory receptor family 2 subfamily M member 3 (127062, *OR2M3*), transgelin 3 (29114, *TAGLN3*), and potassium sodium-activated channel subfamily T member 1 (57582, *KCNT1*)] was downregulated in the EC of male AD patients (*n* = 14, age range: 67-105 years) compared to male controls (*n* = 8, age range: 65-81 years) (Data Appendix B, Data S22). The DEGs were determined based on the criteria of adjusted *p* ≤ 0.05 and │log2FC│ ≥ 1.0.

Functional enrichment analysis showed that the complex interaction network of protein products of downregulated *DIRAS2*, *CPLX1*, *MOBP*, *MBP*, *RPH3A*, *SEPT4*, and *ELMO1* genes in the EC of male AD patients compared to male controls was mainly involved in biological processes related to the exocytic insertion of neurotransmitter receptor to postsynaptic membrane, glial cell apoptotic process, spontaneous neurotransmitter secretion, synaptic vesicle fusion to presynaptic active zone membrane, regulation of synaptic vesicle priming, SNARE complex assembly, glutamate secretion, synaptic vesicle exocytosis, positive regulation of calcium ion-dependent exocytosis, Rac protein signal transduction, leukocyte apoptotic process, synaptic vesicle recycling, and phagocytosis-engulfment (Appendix I) [53,54].

**Figure 7:**
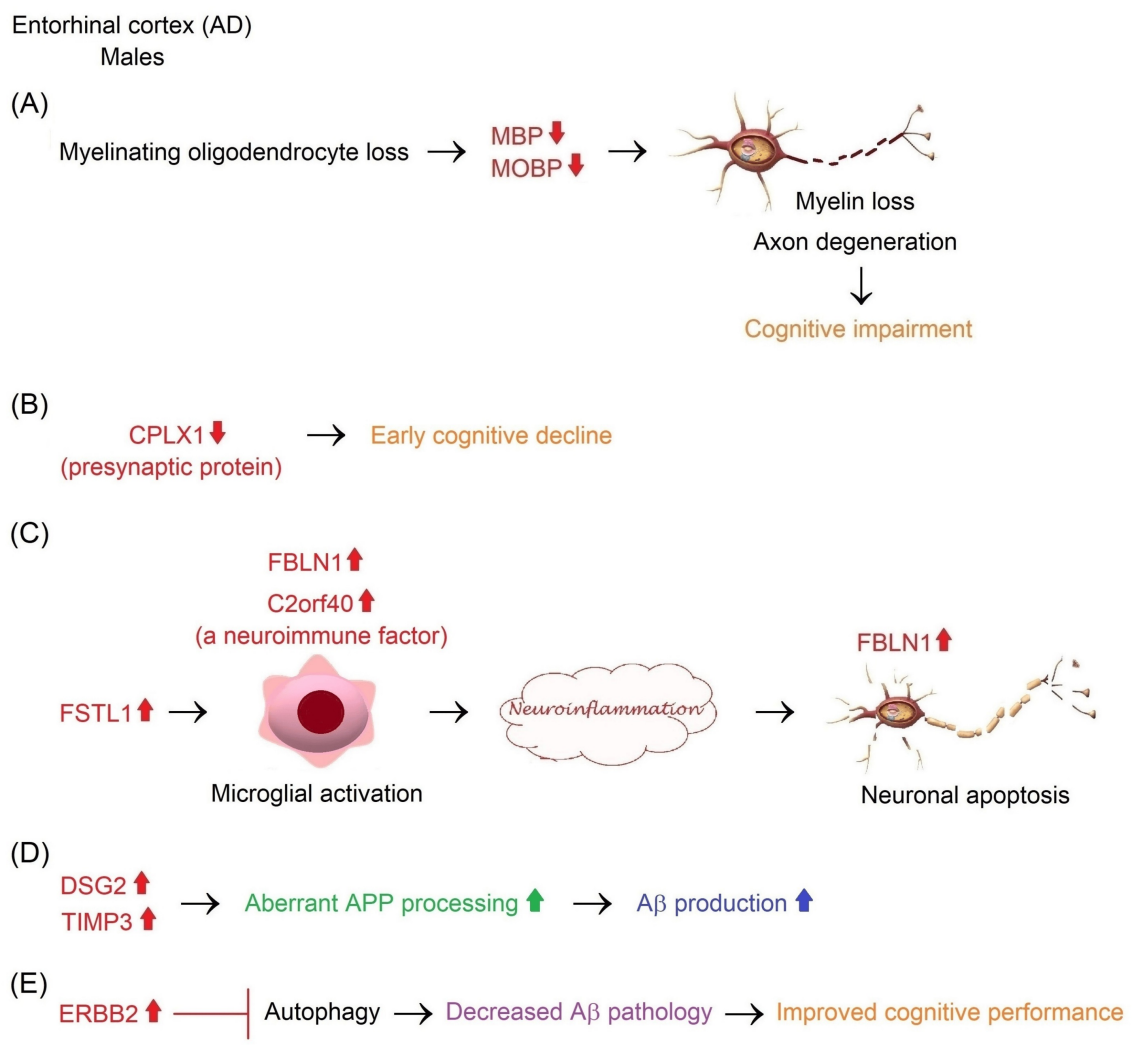
Potential molecular and cellular mechanisms related to some differential gene expressions identified in the EC of male AD patients compared to male controls Image created by the author N.A. solely for the present study using the Microsoft Paint and Paint 3D programs (Microsoft Corp., USA) (A) The loss of myelinating oligodendrocytes causes a decrease in the expression of myelin proteins MBP and MOBP, resulting in myelin loss and axon degeneration in the EC of AD patients. Demyelination and axonal degeneration of certain EC neurons may contribute to early cognitive impairment [234-236,238-240]. (B) Downregulation of the presynaptic protein CPLX1 in the EC may contribute to early cognitive decline in male AD cases [255]. (C) Upregulation of FSTL1 stimulates activated microglia-mediated neuroinflammation and neuronal apoptosis. Upregulation of C2orf40, a neuroimmune factor, in activated microglia may be linked to neuroimmune response and neuroinflammation in the EC of AD brains. In addition, the upregulation of FBLN1 in activated microglia and neurons may contribute to neuronal apoptosis [247,256,257]. (D) Upregulation of DSG2 and TIMP3 increases aberrant APP processing and Aβ production [248,258]. (E) Upregulation of ERBB2 may suppress autophagy via binding to Beclin 1 and thereby lead to increased Aβ pathology in the EC of male AD patients, causing worse cognitive performance [259]. EC: entorhinal cortex, AD: Alzheimer’s disease

The synaptic density is significantly reduced in the neuropil of layers 2 and 3 of the EC in AD cases compared to control subjects [254]. The disruption in myelination is observed in layers 2 and 3 of the EC and the CA1 region of the HC before the occurrence of Aβ and tau pathology in a triple-transgenic mouse model of AD. The expression level of myelin/oligodendrocyte markers MBP and 2',3'-cyclic nucleotide 3' phosphodiesterase (CNPase) decreases in these subregions of the HC and EC [238]. A glycolytic deficiency in oligodendrocytes induces glycolytic stress that triggers inflammatory pyroptotic damage of the mature oligodendrocytes. Oligodendroglial damage causes demyelination and axonal degeneration, resulting in cognitive impairment in AD [239] (Figure 7A).

The lower expression of the presynaptic protein CPLX1, especially in the EC, contributes to cognitive impairment mostly in the early stages of AD. CPLX1 is expressed predominantly in GABAergic inhibitory presynaptic terminals. The disruption of GABAergic presynaptic terminals may cause early cognitive decline [255] (Figure 7B). 

Using the GEO2R tool, the analysis of microarray gene expression data of the GSE118553 dataset revealed that the expression of 78 genes [fibulin 1 (2192, *FBLN1*), neuron derived neurotrophic factor (79625, *NDNF*), FAT atypical cadherin 1 (2195, *FAT1*), enoyl-CoA hydratase domain containing 3 (79746, *ECHDC3*), transient receptor potential cation channel subfamily M member 3 (80036, *TRPM3*), tyrosinase related protein 1 (7306, *TYRP1*), DDB1 and CUL4 associated factor 12 (25853, *DCAF12*), decorin (1634, *DCN*), KDEL (Lys-Asp-Glu-Leu) containing 2 (143888, *KDELC2*), follistatin like 1 (11167, *FSTL1*), desmoglein 2 (1829, *DSG2*), membrane frizzled-related protein (83552, *MFRP*), chromosome 9 open reading frame 135 (138255, *C9orf135*), ANTXR cell adhesion molecule 1 (84168, *ANTXR1*), protocadherin 18 (54510, *PCDH18*), C1q and TNF related 5 (114902, *C1QTNF5*), WAP, follistatin/kazal, immunoglobulin, kunitz and netrin domain containing 2 (124857, *WFIKKN2*), dynein assembly factor with WD repeats 1 (164781, *DAW1*), transmembrane protein 64 (169200, *TMEM64*), Yes1 associated transcriptional regulator (10413, *YAP1*), serpin family I member 2 (5276, *SERPINI2*), IQ motif containing GTPase activating protein 2 (10788, *IQGAP2*), small nucleolar RNA, C/D box 83B (116938, *SNORD83B*), folate receptor alpha (2348, *FOLR1*), peptidylprolyl isomerase C (5480, *PPIC*), erb-b2 receptor tyrosine kinase 2 (2064, *ERBB2*), solute carrier family 16 member 10 (117247, *SLC16A10*), solute carrier family 16 member 9 (220963, *SLC16A9*), claudin 2 (9075, *CLDN2*), RAB11 family interacting protein 1 (80223, *RAB11FIP1*), NAD(P)H quinone dehydrogenase 1 (1728, *NQO1*), dehydrogenase/reductase 3 (9249, *DHRS3*), cilia and flagella associated protein 43 (80217, *CFAP43*), transforming acidic coiled-coil containing protein 1 (6867, *TACC1*), msh homeobox 1 (4487, *MSX1*), cilia and flagella associated protein 206 (154313, *CFAP206*), tetraspanin 6 (7105, *TSPAN6*), syntrophin beta 1 (6641, *SNTB1*), receptor tyrosine kinase like orphan receptor 2 (4920, *ROR2*), RB binding protein 8, endonuclease (5932, *RBBP8*), collectin subfamily member 12 (81035, *COLEC12*), TIMP metallopeptidase inhibitor 3 (7078, *TIMP3*), ATP binding cassette subfamily C member 4 (10257, *ABCC4*), plectin (5339, *PLEC*), coagulation factor V (2153, *F5*), small nucleolar RNA, H/ACA box 35 (677816, *SNORA35*), calcium binding protein 39 like (81617, *CAB39L*), superoxide dismutase 3 (6649, *SOD3*), meiosis specific nuclear structural 1 (55329, *MNS1*), dynein axonemal light intermediate chain 1 (7802, *DNALI1*), annexin A4 (307, *ANXA4*), golgi integral membrane protein 4 (27333, *GOLIM4*), eukaryotic translation elongation factor 1 alpha 1 (1915, *EEF1A1*), atypical chemokine receptor 3 (57007, *ACKR3*), solute carrier family 13 member 4 (26266, *SLC13A4*), parvin alpha (55742, *PARVA*), NPC intracellular cholesterol transporter 2 (10577, *NPC2*), synaptotagmin like 4 (94121, *SYTL4*), TEA domain transcription factor 2 (8463, *TEAD2*), secreted protein acidic and cysteine rich (6678, *SPARC*), TGFB induced factor homeobox 2 (60436, *TGIF2*), integrin subunit alpha 6 (3655, *ITGA6*), MYCBP/AMY-1-associated testis-expressed protein 1 (89876, *MAATS1*), EYA transcriptional coactivator and phosphatase 4 (2070, *EYA4*), carboxypeptidase Q (10404, *CPQ*), adrenoceptor alpha 1A (148, *ADRA1A*), chloride intracellular channel 6 (54102, *CLIC6*), DNA damage inducible transcript 4 like (115265, *DDIT4L*), family with sequence similarity 84 member B (157638, *FAM84B*), solute carrier family 15 member 3 (51296, *SLC15A3*), chromosome 21 open reading frame 62 (56245, *C21orf62*), small nucleolar RNA, H/ACA box 73B (26768, *SNORA73B*), solute carrier family 16 member 4 (9122, *SLC16A4*), INS-IGF2 readthrough (723961, *INS-IGF2*), phosphoinositide interacting regulator of transient receptor potential channels (644139, *PIRT*), nucleoporin 62 C-terminal like (54830, *NUP62CL*), chromosome 2 open reading frame 40 (84417, *C2orf40*), calmodulin like 4 (91860, *CALML4*)] was upregulated in the EC of male AD patients (*n* = 14, age range: 67-105 years) compared to male controls (*n* = 8, age range: 65-81 years) (Data Appendix B, Data S22). The DEGs were determined based on the criteria of adjusted *p* ≤ 0.05 and │log2FC│ ≥ 1.0.

Functional enrichment analysis exhibited that the complex interaction network of protein products of upregulated *FBLN1*, *FAT1*, *DCN*, *FSTL1*, *DSG2*, *WFIKKN2*, *YAP1*, *IQGAP2*, *FOLR1*, *PPIC*, *ERBB2*, *MSX1*, *TIMP3*, *PLEC*, *F5*, *SPARC*, *EEF1A1*, *ITGA6*, *PARVA*, *EYA4*, and *TEAD2* genes in the EC of male AD patients compared to male controls was mainly involved in biological processes related to nail development, lateral mesoderm development, hemidesmosome assembly, regulation of extrinsic apoptotic signaling pathway, regulation of cellular response to growth factor stimulus, regulation of transmembrane receptor protein serine/threonine kinase signaling pathway, and regulation of apoptotic signaling pathway (Appendix J) [53,54].

The EC shows selective vulnerability to neurodegeneration in the early stage of AD. The EC neurons are susceptible to cell damage triggered by oxidative stress and mitochondrial dysfunction. In addition, astrocytic dysfunction, microglial activation, neuroinflammation, and the activation of stress kinases contribute to the early vulnerability of the EC to AD. The neuronal and synaptic degeneration of the EC disrupts hippocampal inputs, leading to early cognitive deficiency [240].

Intracellular Aβ deposition occurs at three months of age and later increases at six months of age in the EC and HC in a triple transgenic mouse model of AD. Extracellular Aβ accumulation does not occur at the early ages of two to six months. The mRNA expression of proinflammatory molecules *TNF-α* and C-C motif chemokine ligand 2 (*CCL2*, also known as *MCP1*) is selectively upregulated in the EC of transgenic mice before overt Aβ plaque pathology. The increase begins at three months of age compared to two months of age, but statistically significant upregulation is observed at six months of age. No significant change is found in the expression level of *TNF-α* and *CCL2* in the HC at these early stages of AD. The increase of *TNF-α* and *CCL2* expression in the EC is correlated with the increased number of microglia and macrophages in the same area. These findings show that an inflammatory response occurs at the early stage of the disease before significant extracellular Aβ accumulation selectively in the EC in the brain of triple transgenic AD mice [260].

The knockdown of *FSTL1* suppresses microglial activation, neuroinflammation, and neuronal apoptosis in AD-like mice, resulting in reduced Aβ plaque and improved synaptic plasticity [256]. The immunoreactivity of C2orf40, a neuroimmune factor, is decreased in neurons of the EC in AD patients compared to controls. However, activated microglial cells that show strong immunoreactivity for C2orf40 are detected more frequently in the WM of the EC in AD cases compared to controls. In addition, C2orf40 staining is prominently observed in NFTs of gray matter in AD. The findings suggest that C2orf40 contributes to the neuroimmune response in AD [257] (Figure 7C).

Knocking down *DSG2* decreases the expression and secretion of Aβ_42_ peptides and suppresses the accumulation of Aβ polymers in an induced neuron model of *APOE ε*3/4 allele-associated sporadic AD with overexpression of mutant APP and hyperphosphorylation of tau, indicating that DSG2 contributes to defective processing of APP and increased Aβ aggregation in sporadic AD [248]. Overexpression of TIMP3 increases β-secretase cleavage of APP in COS7 cells, whereas its overexpression decreases α-cleavage of APP by α-secretase ADAM metallopeptidase domain 10 (ADAM10). This change in APP processing causes increased levels of Aβ_40_ and Aβ_42_ peptides in COS7 cells. Similarly, upregulation of TIMP3 leads to increased Aβ production in primary cortical neurons [258] (Figure 7D).

ERBB2 represses autophagic flux via binding to Beclin 1, which prevents the assembly of the tripartite complex consisting of Beclin 1, phosphatidylinositol 3-kinase catalytic subunit type 3 (PIK3C3, also known as VPS34), and phosphoinositide-3-kinase regulatory subunit 4 (PIK3R4, also known as VPS15) in HEK293 cells. Downregulating ERBB2 attenuates Aβ pathology and improves cognitive performance by activating autophagy in AD models [259] (Figure 7E).

The enzymatic activity of NQO1 increases in amyloid beta plaques and the areas of tau NFT in the HC of AD patients. The authors recommend that NQO1 may exert a neuroprotective effect in response to the progression of AD through its antioxidant activity [261,262].

The expression of FBLN1 increases in neurons and microglia after spinal cord injury in adult rats. Interestingly, FBLN1 is colocalized with caspase-3 in neurons upon spinal cord injury, suggesting that FBLN1 may be implicated in apoptotic neuronal cell death. FBLN1 is expressed in reactive microglia and activated F4/80-positive microglia in the spinal cord, suggesting that FBLN1 may also be involved in microglial activation following spinal cord injury [247] (Figure 7C).

Differential Gene Expressions and Associated Potential Mechanisms Identified in the Hippocampus of Females With AD

Using the GEO2R tool, the analysis of microarray gene expression data of the GSE48350 dataset [45,48,263] demonstrated that the expression of 12 genes [RTF1 homolog, Paf1/RNA polymerase II complex component (23168, *RTF1*), long intergenic non-protein coding RNA 3007 (646588, *LOC646588*/*LINC03007*), ribonucleic acid export 1 (8480, *RAE1*), long intergenic non-protein coding RNA 2987 (101927151, *LOC101927151*/*LINC02987*), NSF attachment protein alpha (8775, *NAPA*), phosphoglycerate kinase 1 (5230, *PGK1*), transmembrane protein 70 (54968, *TMEM70*), attractin like 1 (26033, *ATRNL1*), neurexin 1 (9378, *NRXN1*), cAMP-dependent protein kinase inhibitor beta (5570, *PKIB*), visinin like 1 (7447, *VSNL1*), and interferon related developmental regulator 1 (3475, *IFRD1*)] was downregulated in the HC of female AD patients (*n* = 10, age range: 60-91 years) compared to female controls (*n* = 12, age range: 64-99 years) (Data Appendix B, Data S23). The DEGs were determined based on the criteria of adjusted *p *≤ 0.05 and │log2FC│ ≥ 1.0.

RTF1 is a member of the PAF1 complex (PAF1C), which is conserved among different organisms from yeast to humans. PAF1C functions in the transcription of genes that are required for self-renewal, pluripotency, and differentiation of stem cells. The transcription factor Rtf1 contributes to wing development in *Drosophila* and somite segmentation in zebrafish by regulating Notch signaling. PAF1C also participates in the cotranscriptional modification of histones during the transcription of active genes. It functions primarily in the monoubiquitination of H2B (H2Bub) and methylation of H3K4 and H3K79 [264,265]. Rtf1 promotes the monoubiquitination of histone H2B via direct interaction with the ubiquitin conjugase Rad6 [266].

**Figure 8:**
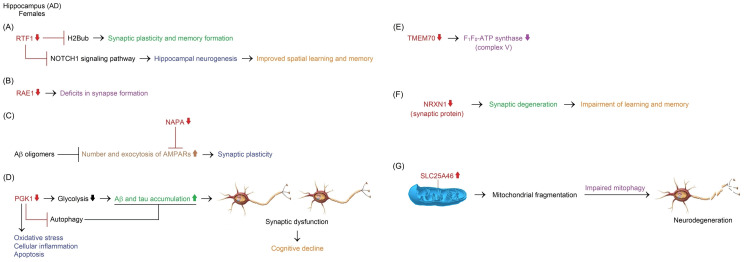
Potential molecular and cellular mechanisms related to some differential gene expressions identified in the HC of female AD patients compared to female controls Image created by the author N.A. solely for the present study using the Microsoft Paint and Paint 3D programs (Microsoft Corp., USA) (A) Downregulation of RTF1 suppresses the monoubiquitination of H2B (H2Bub), possibly leading to the impairment in synaptic plasticity and memory formation in the HC of AD brains. In addition, the downregulation of RTF1 may repress the NOTCH1 signaling that promotes adult hippocampal neurogenesis. The repression of compensatory adult neurogenesis in the DG of the hippocampal formation can limit the improvement in spatial learning and memory [264-268]. (B) The downregulation of RAE1 gives rise to deficits in synapse formation [269]. (C) Oligomers of Aβ suppress the increase in the number and exocytosis of AMPARs during LTP in postsynaptic and extrasynaptic membranes in hippocampal neurons, triggering the impairment in synaptic plasticity. Downregulation of NAPA may cause decreased expression of AMPARs on the cell surface of neurons [270,271]. (D) Downregulation of PGK1 can reduce glycolysis and thereby enhance Aβ and tau accumulation, resulting in synaptic dysfunction and cognitive decline. Reduced expression of PGK1 may also cause the repression of autophagy, increasing Aβ and tau pathology. In addition, its downregulation can induce oxidative stress, cellular inflammation, and apoptosis [272-276]. (E) Downregulation of TMEM70 may contribute to mitochondrial F1F0-ATP synthase deficiency [277,278]. (F) Downregulation of NRXN1 may be involved in synaptic degeneration, resulting in the impairment of learning and memory [279]. (G) Upregulation of SLC25A46 localized in the mitochondrial outer membrane triggers mitochondrial fragmentation. Impaired mitophagy may lead to the accumulation of fragmented mitochondria, contributing to neurodegeneration [280-283]. HC: hippocampus, AD: Alzheimer’s disease, DG: dentate gyrus, AMPAR: α-amino-3-hydroxy-5-methyl-4-isoxazolepropionic acid receptor, LTP: long-term potentiation

H2Bub transiently increases in the CA1 area of the HC in response to learning in rats. It promotes learning-dependent synaptic plasticity and long-term memory formation in the HC. H2Bub elevates the level of trimethylation of histone H3 at lysine 4 (H3K4me3) by recruiting the 19S proteasome subunit RPT6 to mediate the activation of transcription of genes related to synaptic plasticity and memory formation in the hippocampal CA1 region [267], suggesting that RTF1 may contribute to synaptic plasticity and memory formation through the monoubiquitination of histone H2B. Activation of NOTCH1 signaling stimulates the proliferation and differentiation of neural stem cells in the DG of the hippocampal formation and improves spatial learning and memory after traumatic brain injury in adult rats, indicating that the NOTCH1 signaling pathway promotes neurogenesis in the hippocampal zone [268]. In light of available literature, the analysis of the GSE48350 dataset suggests that the downregulation of *RTF1* may be implicated in synaptic dysfunction, learning and memory deficits, and the impairment of adult neurogenesis in the HC of AD brains (Figure 8A).

The cytoplasmic phosphorylated tau drives the depletion of nucleoporin 98 and 96 precursor (NUP98) from the nuclear envelope and its mislocalization to the cytosol in hippocampal CA1 neurons of AD brains. NUP98 is colocalized aberrantly with phosphorylated tau in the cytosol of CA1 neurons. NUP98 facilitates the fibrilization of tau *in vitro*, possibly contributing to the aggregation of tau and the formation of NFT in the somata of AD neurons. Phosphorylated tau impairs the nuclear import and export of proteins through the mislocalization of NUP98 and the disruption of the nuclear pore complex diffusion barrier in hippocampal nuclei from AD brains [284]. RAE1 is a transport protein that shuttles between the nucleus and the cytoplasm. It functions in the nuclear export of mRNAs via binding to a specific motif of NUP98 at the nuclear pore complex. RAE1 is primarily localized in the nuclear envelope; however, it is also present in the nucleus and cytoplasm [285]. In addition, RAE1 is found in the perisynaptic zone of presynaptic terminals. The loss of RAE1 function causes deficits in axon termination and synapse formation [269] (Figure 8B).

The overexpression of *NAPA* (also known as *α-SNAP*) increases the expression of AMPA receptors on the cell surface of hippocampal neurons [270]. Oligomers of Aβ inhibit the elevation in the number of the subunit GluA1-containing AMPARs and the frequency of their exocytosis in postsynaptic and extrasynaptic membranes during LTP in hippocampal neurons, causing the impairment of synaptic plasticity. Oligomers of Aβ trigger the retraction of the dendritic spine [271] (Figure 8C).

PGK1 catalyzes the production of ATP by mediating phosphate transfer from 1,3-bisphosphoglycerate to adenosine 5′-diphosphate (ADP) in aerobic glycolysis. It also catalyzes the reverse reaction of the same step in gluconeogenesis. On the other hand, PGK1 functions in the induction of autophagy by phosphorylating Beclin 1 as well [272,273]. The activation of PGK1 reduces apoptosis and thereby exerts a neuroprotective effect after neuronal damage caused by the oxygen-glucose stripping reperfusion (OGD/R) model in rat pheochromocytoma PC12 cells. Moreover, the activation of PGK1 protects against oxidative stress and cellular inflammation [274]. Reduced glycolytic flux can enhance amyloid plaque load and tau accumulation and worsen the severity of disease in AD. The glycolytic impairment may create defects in metabolic and synaptic homeostasis, leading to misfolded protein aggregation and cognitive decline in AD [275] (Figure 8D). The inner mitochondrial membrane proteins TMEM70 and TMEM242 contribute to the assembly of the c_8_-ring of human ATP synthase [277]. The concentration of mitochondrial F_1_F_0_-ATP synthase (complex V) is statistically significantly reduced in the HC of AD patients compared to the control group [278] (Figure 8E).

The expression of *NRXN1* is reduced in the HC of 12-month-old mice with the expression of APP harboring a mutation linked to familial AD/early-onset AD compared to control animals. The authors conclude that downregulation of synaptic proteins NRXN1, CPLX1, neurofilament light chain (NEFL), SYT1, synaptosome-associated protein 25 (SNAP25), and syntaxin 1A (STX1A) in the HC may be linked with synaptic degeneration and the impairment of learning and memory in the brain of AD mice [279] (Figure 8F).

Using the GEO2R tool, the analysis of microarray gene expression data of the GSE48350 dataset revealed that the expression of two genes [solute carrier family 25 member 46 (91137, *SLC25A46*) and zinc finger protein 621 (285268, *ZNF621*)] was upregulated in the HC of female AD patients (*n* = 10, age range: 60-91 years) compared to female controls (*n* = 12, age range: 64-99 years) (Data Appendix B, Data S23). The DEGs were determined based on the criteria of adjusted *p* ≤ 0.05 and │log2FC│ ≥ 1.0.

The overexpression of SLC25A46 localized in the mitochondrial outer membrane causes mitochondrial fragmentation and the disruption of the mitochondrial network in the neurites of cultured neurons derived from the brain of zebrafish embryos [280]. Damaged or dysfunctional mitochondria are exposed to excessive DRP1-mediated fission followed by increased fragmentation in AD brains, suggesting that impaired mitophagy may cause defects in the elimination of fragmented mitochondria, resulting in the accumulation of fragmented mitochondria [281]. DRP1 is colocalized with Aβ oligomers in hippocampal neurons of APP transgenic mice. The accumulation of oligomeric Aβ causes the degeneration of primary hippocampal neurons. In addition, DRP1 is colocalized with hyperphosphorylated tau in the HC of AD brains. These findings suggest that excessive DRP1-mediated mitochondrial fragmentation contributes to neurodegeneration in AD [282,283] (Figure 8G).

Differential Gene Expressions and Associated Potential Mechanisms Identified in the Hippocampus of Males With AD

Using the GEO2R tool, the analysis of microarray gene expression data of the GSE48350 dataset demonstrated that the expression of four genes [RTF1 homolog, Paf1/RNA polymerase II complex component (23168, *RTF1*), long intergenic non-protein coding RNA 3007 (646588, *LOC646588*/*LINC03007*), long intergenic non-protein coding RNA 2987 (101927151, *LOC101927151*/*LINC02987*), and ribonucleic acid export 1 (8480, *RAE1*)] was downregulated in the HC of male AD patients (*n* = 9, age range: 76-94 years) compared to male controls (*n* = 10, age range: 75-97 years) (Data Appendix B, Data S24). The DEGs were determined based on the criteria of adjusted *p *≤ 0.05 and │log2FC│ ≥ 1.0.

Like in the HC of female AD patients compared to female controls, the analysis of the GSE48350 dataset exhibited that the expression of *RTF1*, *LOC646588*, *LOC101927151*, and *RAE1* genes was also downregulated in the HC of male AD patients compared to male controls. Downregulation of *RTF1* may be involved in synaptic dysfunction, learning and memory deficits, and the impairment of adult neurogenesis in the HC of AD brains [264-268]. The loss of *RAE1* function leads to deficits in axon termination and synapse formation [269]. Downregulation of *RAE1* and *RTF1* may exert similar effects on hippocampal neurons of male and female brains in AD. The mechanisms underlying the effects of the loss of their function, particularly on synaptic transmission, could be fully uncovered by *in vitro* and *in vivo* models of AD.

Using the GEO2R tool, the analysis of microarray gene expression data of the GSE48350 dataset revealed that the expression of two genes [FRAS1-related extracellular matrix 3 (166752, *FREM3*) and ankyrin repeat and IBR domain-containing 1 (54467, *ANKIB1*)] was upregulated in the HC of male AD patients (*n* = 9, age range: 76-94 years) compared to male controls (*n *= 10, age range: 75-97 years) (Data Appendix B, Data S24). The DEGs were determined based on the criteria of adjusted *p* ≤ 0.05 and │log2FC│ ≥ 1.0.

Differential gene expression in stage 4S neuroblastoma: further insights into potential mechanisms underlying spontaneous regression

Spontaneous regression can occur in many different cancers. However, the frequency of spontaneous regression changes based on the type of tumor. Neuroblastoma is among the tumors that are more prone to spontaneous regression [286]. Spontaneous regression is described as the partial or complete disappearance of a tumor mass that is not exposed to any treatment. Neuroblastoma patients aged less than one year with stage 4S without *MYCN* amplification are more likely to experience spontaneous regression of their tumor. Spontaneous regression can be observed in the primary and/or metastatic tumor sites. The most common metastatic sites of stage 4S neuroblastoma are the liver, bone marrow, and skin [37,286]. The potential mechanisms of spontaneous regression in neuroblastoma are involved in apoptosis, differentiation, impaired DNA repair, adaptive T cell-mediated immunity, NGF deprivation, telomere shortening, and epigenetic control [37].

Stage 4S neuroblastoma patients compared to stage 4 neuroblastoma patients show significantly higher five-year overall and event-free survival rates. Moreover, stage 4S neuroblastoma patients with *MYCN* amplification have better outcomes than stage 4 neuroblastoma patients with *MYCN* amplification. These results indicate that stage 4S neuroblastoma, compared to stage 4 neuroblastoma, demonstrates different favorable biological characteristics [287,288].

Using the GEO2R tool, the analysis of microarray gene expression data of the GSE45547 dataset [45,48,289] demonstrated that the expression of 14 genes [asparaginase and isoaspartyl peptidase 1 (80150, *ASRGL1*), TOB1 antisense RNA 1 (400604, *TOB1-AS1*/*LOC400604*), RAB39B, member RAS oncogene family (116442, *RAB39B*), proline-rich 7, synaptic (80758, *PRR7*), chromobox 8 (57332, *CBX8*), flap structure-specific endonuclease 1 (2237, *FEN1*), transmembrane O-mannosyltransferase targeting cadherins 1 (83857, *TMTC1*), firre intergenic repeating RNA element (286467, *FIRRE*/*LINC01200*/*LOC286467*), DPY30 domain-containing 2 (84332, *DYDC2*), serine/threonine kinase 33 (65975, *STK33*), musashi RNA-binding protein 2 (124540, *MSI2*), double homeobox A pseudogene 10 (503639, *DUXAP10*), PRAME nuclear receptor transcriptional regulator (23532, *PRAME*), and calcitonin-related polypeptide beta (797, *CALCB*)] was downregulated in primary tumors from 78 stage 4S neuroblastoma cases compared to primary tumors from 214 stage 4 neuroblastoma cases (Data Appendix B, Data S25). The DEGs were determined based on the criteria of adjusted *p* ≤ 0.05 and │log2FC│ ≥ 1.0.

**Figure 9:**
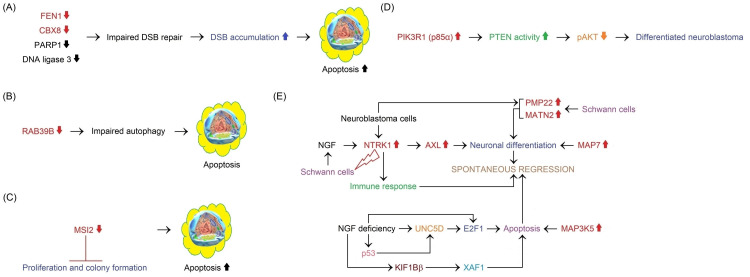
Potential molecular and cellular mechanisms of spontaneous regression related to some differential gene expressions identified in stage 4S neuroblastoma patients compared to stage 4 neuroblastoma patients Image created by the author N.A. solely for the present study using the Microsoft Paint and Paint 3D programs (Microsoft Corp., USA) (A) Downregulation of FEN1, CBX8, PARP1, and DNA ligase 3 causes impaired DSB repair. Disrupted DSB repair can lead to enhanced DSB accumulation, resulting in increased apoptotic cell death in stage 4S neuroblastoma. Downregulation of FEN1, PARP1, and DNA ligase 3 involving MMEJ may also protect neuroblastoma tumors prone to regression against chromosomal rearrangements resulting from the error-prone repair mechanism [290-293,295-302]. (B) Downregulation of RAB39B may cause impaired autophagy, contributing to apoptosis [303]. (C) Downregulation of MSI2 may repress proliferation and colony formation and increase apoptosis [304]. (D) Upregulation of PIK3R1 increases the lipid phosphatase activity of PTEN, causing decreased pAKT levels. The tumor suppressor PTEN is upregulated in well-differentiated neuroblastomas [305-307]. (E) Upregulation of NTRK1 stimulates the proliferation and migration of Schwann cells. Schwann cells then induce the differentiation of neuroblastoma cells by secreting NGF. NTRK1 may play a pivotal role in spontaneous regression by promoting NGF-dependent neuronal differentiation and enhancing the immune response. Also, PMP22 and MATN2 may contribute to neuronal differentiation. MAP7 linked to neuronal differentiation is significantly upregulated in stage 4S neuroblastoma. The deficiency of NGF may upregulate the expression of UNC5D, E2F1, and p53. The interaction between E2F1 and the intracellular fragment of UNC5D transactivates the pro-apoptotic target genes. KIF1Bβ and XAF1 may mediate apoptosis in response to the deficiency of NGF. Upregulation of MAP3K5 may also contribute to apoptosis [308-318]. DSB: double-strand break, MMEJ: microhomology-mediated end-joining, PTEN: phosphatase and tensin homolog, NGF: nerve growth factor

FEN1, a structure-specific endonuclease that removes single-stranded flaps, functions in long-patch base excision repair in vertebrate cells. The stalling of replication forks augments, the rate of DNA replication slows, and the apoptosis increases after DNA damage induced by H_2_O_2_ in FEN1-null cells [290,291]. FEN1 also plays a role in homologous recombination (HR) and nonhomologous end-joining (NHEJ), which are DSB repair mechanisms, in eukaryotes [292,293]. Overexpression of *FEN1* is correlated with poor overall survival, high risk, and advanced stage in neuroblastoma [294]. CBX8, a chromodomain-containing protein, is recruited to the sites of DNA damage upon the activation of poly(ADP-ribose) polymerase 1 (PARP1). The knockdown of *CBX8* decreases the efficiency of NHEJ and HR, exhibiting that CBX8 is essential for efficient DNA repair [295] (Figure 9A).

The alternative forms of NHEJ are microhomology-mediated end-joining (MMEJ), alternative NHEJ (alt-NHEJ), and alternative end-joining (a-EJ). MMEJ, alt-NHEJ, and a-EJ can cause chromosomal rearrangements [296-299]. The MRN complex (MRE11 homolog, double-strand break repair nuclease (MRE11), RAD50 double-strand break repair protein (RAD50), and nibrin (NBN/NBS1)), X-ray repair cross-complementing 1 (XRCC1), FEN1, PARP1, and DNA ligase 3 function in MMEJ [300]. The expression of *FEN1*, *PARP1*, and DNA ligase 3 significantly increases in high-risk neuroblastoma. The inhibition of DNA ligase 3 or PARP1 enhances the accumulation of DSBs and promotes apoptotic cell death in neuroblastoma cells. The high expression of DNA ligase 3, *PARP1*, and *FEN1* is significantly associated with poor overall survival in neuroblastoma [301,302] (Figure 9A).

RAB39B is required for the survival of SH-SY5Y dopaminergic neuroblastoma cells. Downregulation of *RAB39B* expression leads to impaired autophagy and results in apoptosis in these dopaminergic cells. The apoptosis depends on caspases 3 and 9 [303] (Figure 9B). The high expression of *STK33* is significantly associated with poor overall and event-free survival in neuroblastoma [319]. *MSI2* is highly expressed in neuroblastoma. Its increased expression is associated with a worse prognosis in neuroblastoma. The knockdown of *MSI2* decreases the proliferation and colony formation and increases apoptosis in human neuroblastoma cell lines [304] (Figure 9C). *PRAME* is significantly higher expressed in stage 4 tumors than in stages 1-3 and 4S tumors in neuroblastoma [320].

Using the GEO2R tool, the analysis of microarray gene expression data of the GSE45547 dataset showed that the expression of 59 genes [aldehyde dehydrogenase 3 family member A2 (224, *ALDH3A2*), adenylate cyclase 1 (107, *ADCY1*), microtubule-associated protein 7 (9053, *MAP7*), EPS8-like 1 (54869, *EPS8L1*), peripheral myelin protein 22 (5376, *PMP22*), A-kinase anchoring protein 7 (9465, *AKAP7*), serine protease 35 (167681, *PRSS35*), uridine phosphorylase 2 (151531, *UPP2*), hydroxysteroid 17-beta dehydrogenase 3 (3293, *HSD17B3*), ankyrin repeat and fibronectin type III domain-containing 1 (162282, *ANKFN1*), plexin A4 (91584, *PLXNA4*/*PLXNA4B*), BMP/retinoic acid-inducible neural-specific 2 (57795, *BRINP2*/*FAM5B*), myosin XVA (51168, *MYO15A*), fatty acid hydroxylase domain-containing 2 (10826, *FAXDC2*/*C5orf4*), Schlafen-like 1 (200172, *SLFNL1*), cytochrome P450 family 4 subfamily V member 2 (285440, *CYP4V2*), AXL receptor tyrosine kinase (558, *AXL*), Rho guanine nucleotide exchange factor 10-like (55160, *ARHGEF10L*), EYA transcriptional coactivator and phosphatase 4 (2070, *EYA4*), copine 8 (144402, *CPNE8*), family with sequence similarity 228 member A (653140, *FAM228A*/*FLJ30851*), sperm microtubule inner protein 6 (84688, *SPMIP6*/*C9orf24*), peroxidasin (7837, *PXDN*), kinesin family member 13A (63971, *KIF13A*), coiled-coil domain-containing 158 (339965, *CCDC158*/*FLJ25770*), copine 2 (221184, *CPNE2*), secreted frizzled-related protein 1 (6422, *SFRP1*), erythrocyte membrane protein band 4.1-like 3 (23136, *EPB41L3*), cingulin-like 1 (84952, *CGNL1*), parathyroid hormone 1 receptor (5745, *PTH1R*/*PTHR1*), synaptotagmin 2 (127833, *SYT2*/*LOC100127887*), macrophage stimulating 1 like (pseudogene) (11223, *MST1L*/*MSTP9*), collagen type XXVII alpha 1 chain (85301, *COL27A1*), collagen type XXI alpha 1 chain (81578, *COL21A1*), uncharacterized LOC439938 (439938, *LOC439938*), angiotensinogen (183, *AGT*), mitogen-activated protein kinase kinase kinase 5 (4217, *MAP3K5*), phospholipase C delta 4 (84812, *PLCD4*), solute carrier organic anion transporter family member 3A1 (28232, *SLCO3A1*), macrophage stimulating 1 (4485, *MST1*), high mobility group AT-hook 2 (8091, *HMGA2*), erythrocyte membrane protein band 4.1-like 4B (54566, *EPB41L4B*), leucine rich single-pass membrane protein 1 (286006, *LSMEM1*/*C7orf53*), aldehyde dehydrogenase 8 family member A1 (64577, *ALDH8A1*), matrilin 2 (4147, *MATN2*), endothelial PAS domain protein 1 (2034, *EPAS1*), hypothetical protein FLJ23556 (79938, *FLJ23556*/*LOC100505968*), zinc finger and BTB domain-containing 20 (26137, *ZBTB20*), TLR4 interactor with leucine rich repeats (9865, *TRIL*/*KIAA0644*), platelet endothelial aggregation receptor 1 (375033, *PEAR1*), cytochrome b5 reductase 2 (51700, *CYB5R2*), POF1B actin binding protein (79983, *POF1B*), WD repeat and SOCS box-containing 1 (26118, *WSB1*), immunoglobulin superfamily member 10 (285313, *IGSF10*), phosphoinositide-3-kinase regulatory subunit 1 (5295, *PIK3R1*), transmembrane protein with EGF-like and two follistatin-like domains 2 (23671, *TMEFF2*), neurotrophic receptor tyrosine kinase 1 (4914, *NTRK1*), laminin subunit alpha 4 (3910, *LAMA4*), and LON peptidase N-terminal domain and ring finger 2 (164832, *LONRF2*)] was upregulated in primary tumors from 78 stage 4S neuroblastoma cases compared to primary tumors from 214 stage 4 neuroblastoma cases (Data Appendix B, Data S25). The DEGs were determined based on the criteria of adjusted *p* ≤ 0.05 and │log2FC│ ≥ 1.0.

*PIK3R1* encodes three regulatory subunits p85α, p55α, and p50α of class IA phosphatidylinositol 3-kinase (PI3K) [305]. The p85α subunit enhances the lipid phosphatase activity of phosphatase and tensin homolog (PTEN) via directly binding it, causing decreased phosphorylated AKT serine/threonine kinase (pAKT) levels [306]. The expression of tumor suppressor PTEN protein is downregulated in aggressive undifferentiated neuroblastomas compared to well-differentiated neuroblastomas [307]. The mRNA of *PIK3R1* and p85α, p55α, and p50α isoforms encoded by *PIK3R1* are significantly higher expressed in stage 1-2 neuroblastoma than in stage 4 neuroblastoma. But pAKT is significantly downregulated in stage 1-2 neuroblastoma compared to stage 4 neuroblastoma [321,322]. A pan-cancer analysis demonstrates that higher expression of *PIK3R1* is significantly associated with a better overall survival rate in many solid tumors, including lung adenocarcinoma, stomach adenocarcinoma, kidney renal clear cell carcinoma, and liver hepatocellular carcinoma, suggesting a tumor-suppressive role for PIK3R1 [323] (Figure 9D).

NGF upregulates the expression of the TYRO3 protein tyrosine kinase (TYRO3) and AXL receptors via the activation of the TRKA (also known as NTRK1) receptor during neuronal differentiation of PC12 cells [308]. The high expression of *TRKA* is significantly associated with increased leukocyte infiltration in low-stage neuroblastoma. These results suggest that TRKA may contribute to the spontaneous regression of neuroblastoma tumors through both the induction of differentiation and the enhancement of the immune response [309]. The expression of *NTRK1* in neuroblastoma cells stimulates the proliferation and migration of Schwann cells by inducing the secretion of neuregulin 1 (NRG1). Schwann cells then secrete NGF that promotes the differentiation of neuroblastoma cells with NTRK1 expression [310] (Figure 9E).

The withdrawal of NGF from terminally differentiated SH-SY5Y neuroblastoma cell cultures causes apoptotic cell death [311]. The withdrawal of NGF strongly increases the expression of unc-5 netrin receptor D (UNC5D), E2F1, and p53 in favorable human primary neuroblastoma cells. The cleavage of UNC5D by caspases 2 and 3 triggers the translocation of its intracellular fragment into the nucleus. Later, this fragment interacts with E2F1, resulting in the transactivation of pro-apoptotic target genes. These findings suggest that UNC5D mediates NGF withdrawal-induced apoptosis during spontaneous regression of neuroblastoma [312]. Kinesin family member 1B beta (KIF1Bβ) and XIAP-associated factor 1 (XAF1) mediate apoptosis in neuroblastoma cells. XAF1 is required for NGF withdrawal-induced apoptosis [313] (Figure 9E). On the other hand, NGF promotes apoptosis in human SK-N-MC neuroblastoma cells expressing nerve growth factor receptor (p75^NTR^/NGFR) but not TRKA; however, it does not cause the death of human IMR32 neuroblastoma cells expressing TRKA but not p75^NTR^ [324].

The high expression of *PLXNA4* is significantly associated with a better event-free survival rate in neuroblastoma patients [325]. In line with the findings of the GEO2R analysis of the GSE45547 dataset, the expression of *MAP7* related to neuronal differentiation is significantly increased in stage 4S neuroblastoma compared to stage 4 neuroblastoma [314] (Figure 9E).

PMP22, an integral membrane glycoprotein found in the myelin sheath, is mainly expressed by Schwann cells in the peripheral nervous system [315]. Downregulation of tumor suppressor genes *PMP22* and *MATN2* is associated with high-risk neuroblastoma tumors [326]. *MATN2* and glial differentiation marker *PMP22* are among the upregulated genes induced by GLI family zinc finger 1 (GLI1) in SH-SY5Y neuroblastoma cells. GLI1 promotes the differentiation of SH-SY5Y cells by inducing a specific gene expression profile resembling that of benign ganglioneuroma. GLI1 has the potential to promote the differentiation into both neurons and glia [316]. MATN2 protein is upregulated and secreted into the extracellular matrix by Schwann cells around the injured peripheral nerves of adult mice. MATN2 promotes the adhesion and migration of Schwann cells. MATN2 contributes to axonal regeneration upon damage to the peripheral nerves [317] (Figure 9E).

The expression of *CPNE2* is significantly reduced in stage 4 neuroblastoma. The low expression of *CPNE2* is significantly associated with a worse overall survival rate [327]. Apoptosis signal-regulating kinase 1 (ASK1, also known as MAP3K5) induces apoptosis through the activation of p38 MAPK and JNK in human SH-SY5Y dopaminergic neuroblastoma cells exposed to 6-hydroxydopamine (6-OHDA) [318] (Figure 9E). The high expression of *EPAS1*, also known as hypoxia-inducible factor 2 alpha (*HIF2α*), is significantly associated with a better overall survival rate in neuroblastoma. The expression of *EPAS1* is significantly positively correlated with the expression of some genes, including *NTRK1*, which are involved in neuronal differentiation, in neuroblastoma [328]. *WSB1* is overexpressed in stages 1-3 and 4S neuroblastoma tumors compared to stage 4 neuroblastoma tumors. The higher expression of *WSB1* is significantly associated with a better outcome in neuroblastoma [329].

Limitations of the present study

Taken together, the present analytical review provides novel insights into potential molecular and cellular mechanisms underlying neuroprotection, neurodegeneration, and spontaneous regression based on the DEG analyses and relevant published literature information in normal brain aging, AD, and stage 4S neuroblastoma. However, the present analytical review has some limitations. In this analytical review, the DEG analyses have been performed on microarray gene expression data from the GEO datasets using the GEO2R tool. Differential gene expressions identified in the present analytical review need to be verified at the protein level. The conclusions derived from the DEG analyses and relevant published literature information of the present analytical review should be further experimentally validated.

## Conclusions

The present analytical review exhibits gender- and region-specific differential gene expressions and related differential molecular and cellular mechanisms in the normal aging brain and AD brain. PRH1 and *MIR181A1HG* may exert neuroprotective effects in certain brain areas of elderly females. The loss of myelinating oligodendrocytes in the EC and repressed adult hippocampal neurogenesis may contribute to cognitive impairment and spatial learning and memory deficits, respectively, in both females and males with AD. The downregulation of the CARNS1 enzyme that catalyzes the synthesis of carnosine can repress the production of carnosine and thereby increase neuronal vulnerability to the toxic effects of Aβ_1-42_ oligomers in the EC of female AD patients. The present analytical review suggests that endogenous carnosine is likely to exert neuroprotective effects against impaired synaptic plasticity and neuronal loss during normal brain aging. Exogeneous carnosine treatment may hold promise for both prevention and therapy of AD. The canonical Wnt/β-catenin signaling pathway may play a neuroprotective role by suppressing gliosis and Aβ pathology in AD. SFRP1 may enhance Aβ pathology via both the repression of the canonical Wnt signaling pathway and its accumulation in Aβ plaques in the EC of female AD patients. Glial cell-derived SFRP1 can localize to the synapses and cause synaptic dysfunction, resulting in cognitive decline. Upregulation of ERBB2 may cause impaired autophagy via binding to Beclin 1 and thus lead to increased Aβ pathology in the EC of male AD patients. The high expression of *ERBB2* in the EC may be associated with worse cognitive performance in AD. The downregulation of *NAPA*, *NRXN1*, and *PGK1* in the HC of female AD patients may contribute to synaptic impairments, leading to learning and memory deficits. Downregulation of *PGK1* may contribute to cognitive decline in female AD patients by increasing Aβ and tau pathology through the suppression of glycolysis and autophagy in the HC.

Neurodegeneration of mature neurons and spontaneous regression of immature sympathetic neuroblast-derived neuroblastomas may occur via similar cellular behavior of mature and immature nerve cells. Impaired autophagy and apoptotic cell death can play central roles in both neurodegeneration of AD and spontaneous regression of stage 4S neuroblastoma. However, adult hippocampal neurogenesis may be repressed in AD. Conversely, neuronal differentiation can contribute to spontaneous regression in stage 4S neuroblastoma. The present analytical review provides novel insights into potential mechanisms underlying spontaneous regression such as apoptosis, neuronal differentiation, immune response, impaired autophagy, and impaired DSB repair in stage 4S neuroblastoma. Downregulation of repair proteins FEN1 and CBX8 may cause impaired DSB repair that leads to enhanced DSB accumulation, increasing apoptosis in stage 4S neuroblastoma. The downregulation of FEN1 involving MMEJ may also help protect stage 4S neuroblastoma tumors from chromosomal rearrangements resulting from the error-prone repair mechanism. NTRK1 may play a pivotal role in spontaneous regression of stage 4S neuroblastoma through both the induction of the NGF-dependent neuronal differentiation and the enhancement of the immune response. However, the exact role of the AXL receptor, which functions in the NGF-dependent neuronal differentiation pathway, remains unknown in stage 4S neuroblastoma. Also, the mechanisms underlying the enhancement of the immune response by NTRK1 and the connection between the immune response and spontaneous regression remain to be identified in stage 4S neuroblastoma. On the other hand, NGF deficiency-dependent apoptotic pathways remain to be investigated in spontaneous regression of stage 4S neuroblastoma.

## Figures and Tables

**Figure 10:**
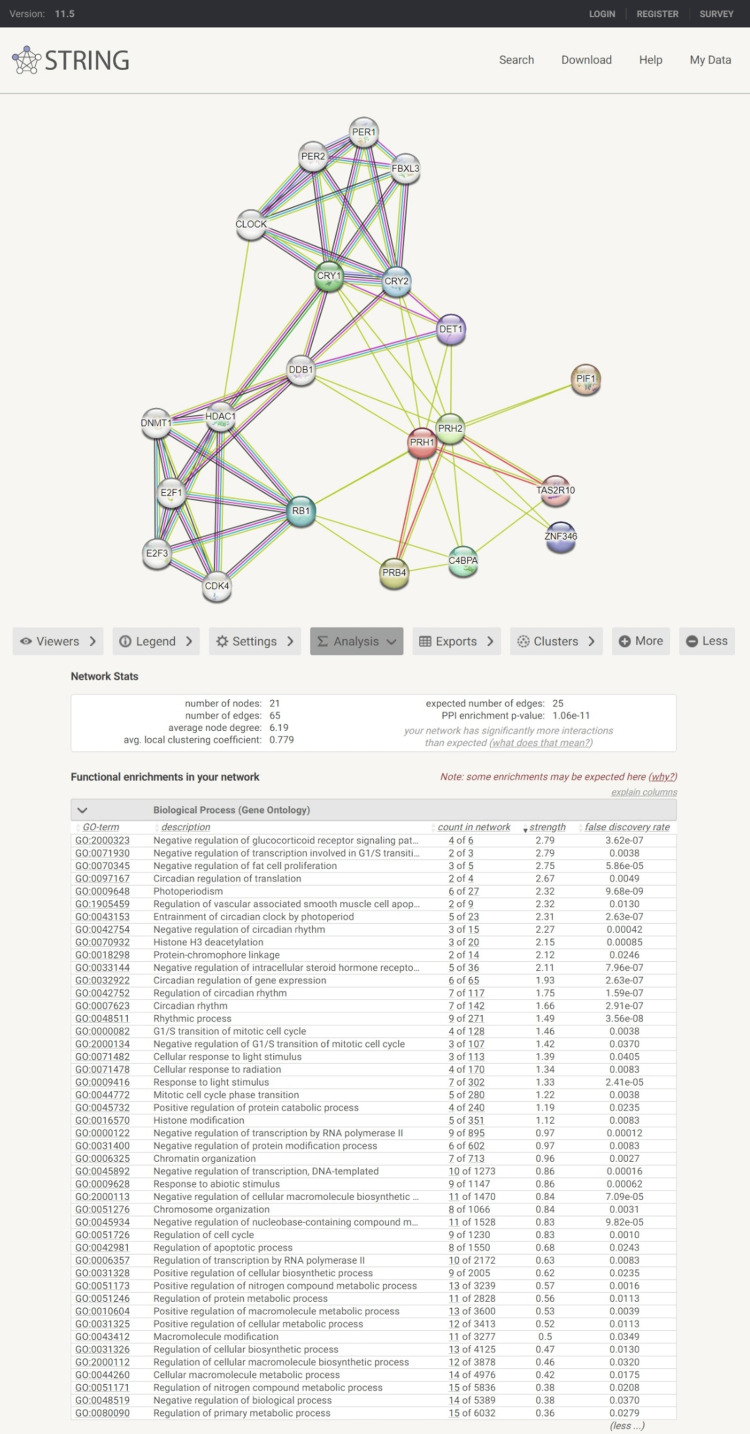
Functional enrichment analysis of PRH1 performed using the STRING database

**Figure 11:**
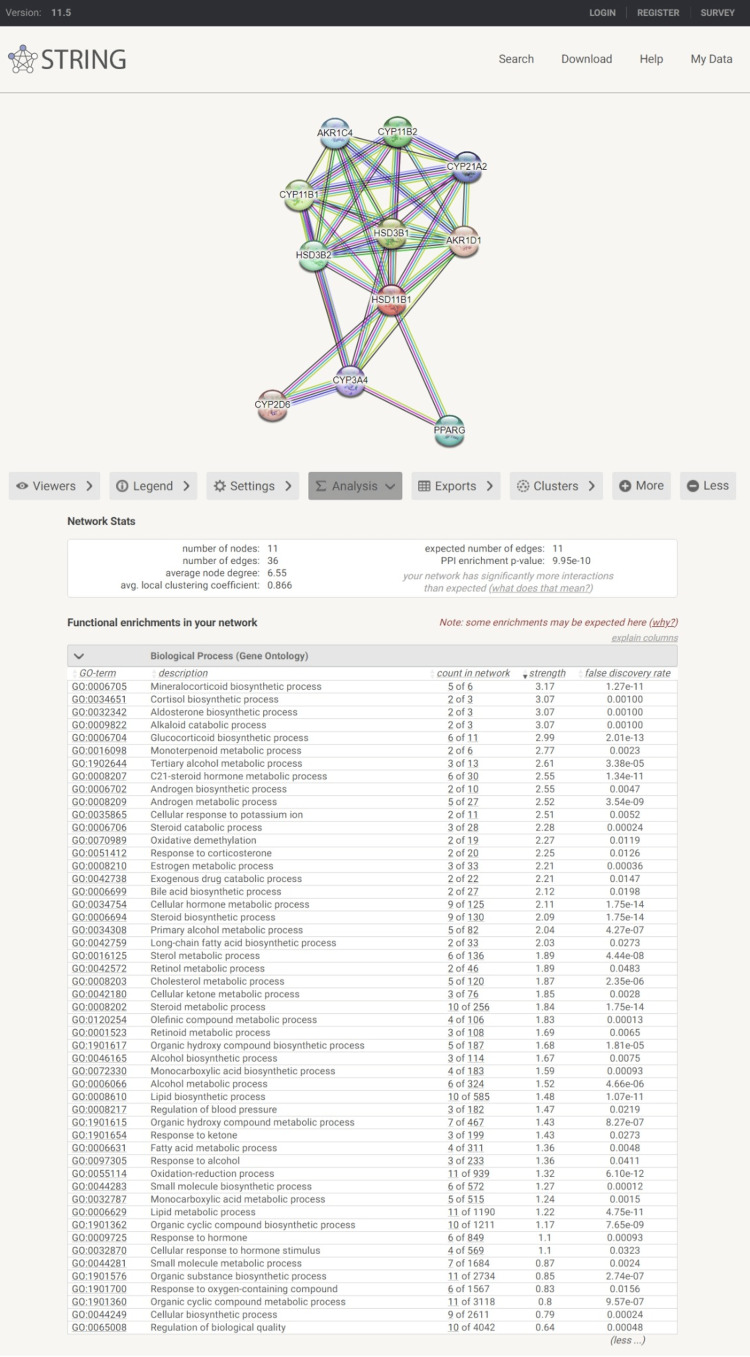
Functional enrichment analysis of HSD11B1 performed using the STRING database

**Figure 12:**
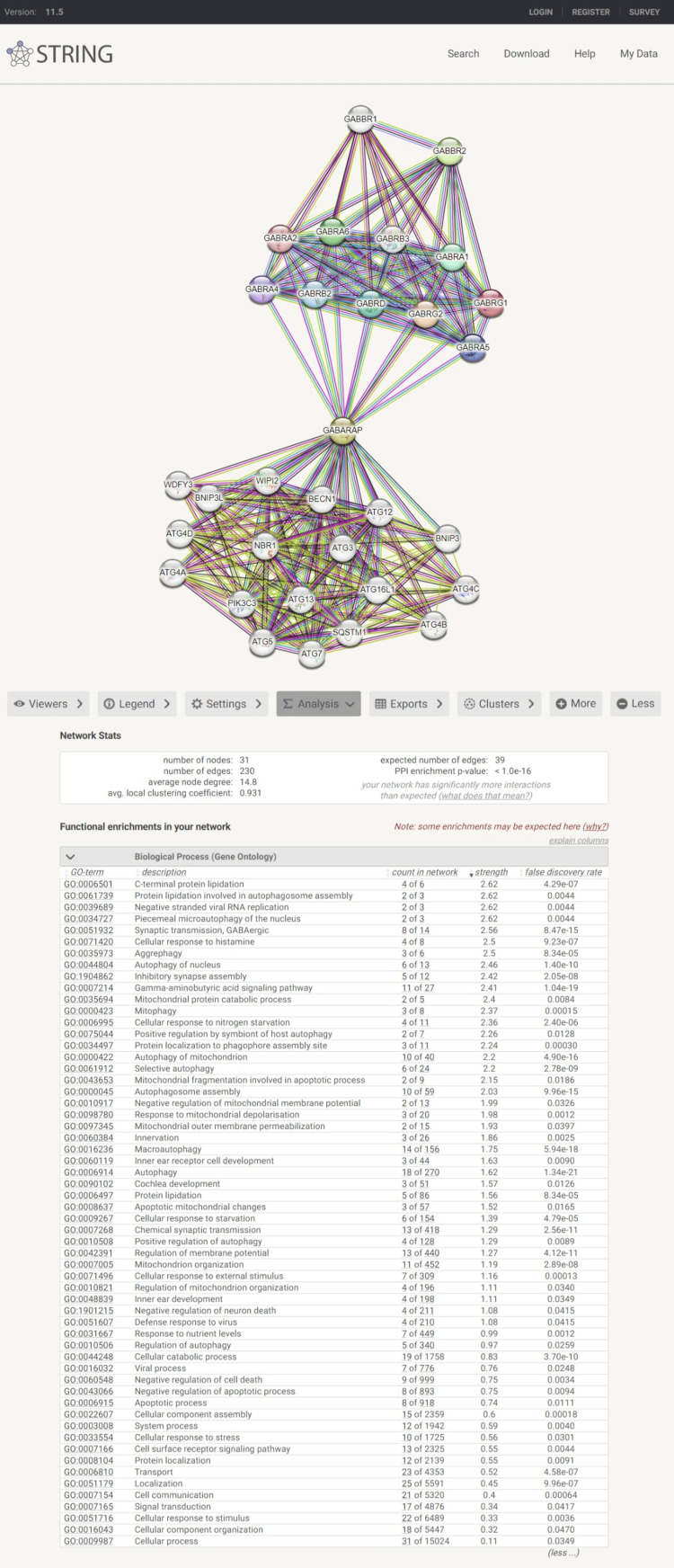
Functional enrichment analysis of GABRG1 performed using the STRING database

**Figure 13:**
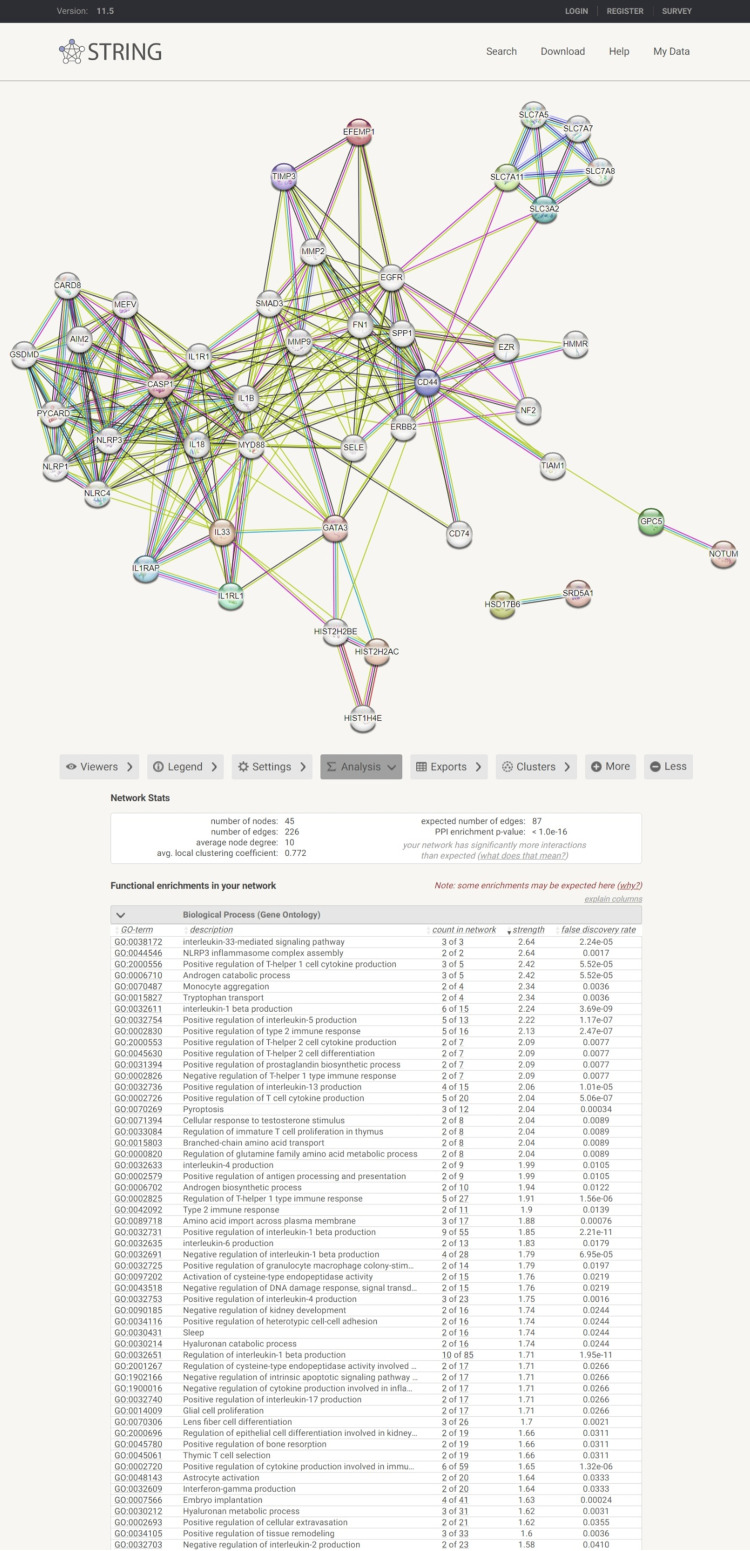
Functional enrichment analysis of IL33, EFEMP1, SLC7A11, GPC5, and HSD17B6 performed using the STRING database

**Figure 14:**
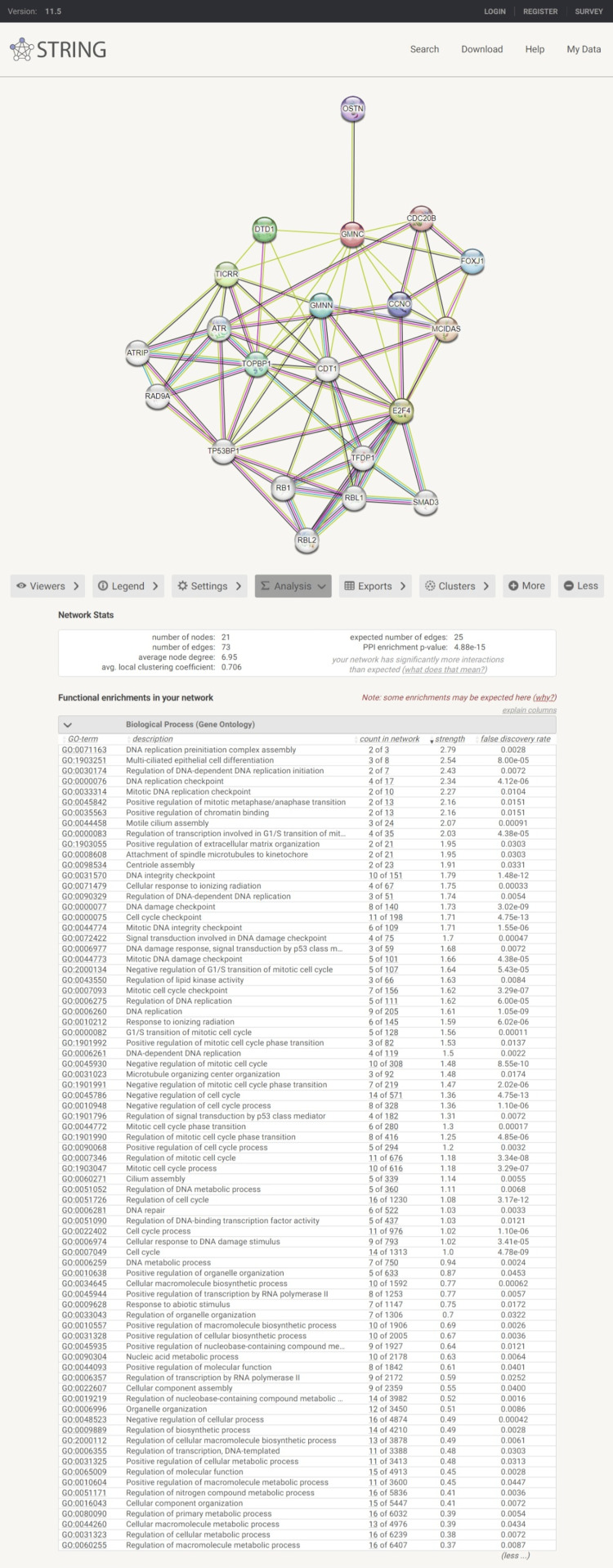
Functional enrichment analysis of GMNC performed using the STRING database

**Figure 15:**
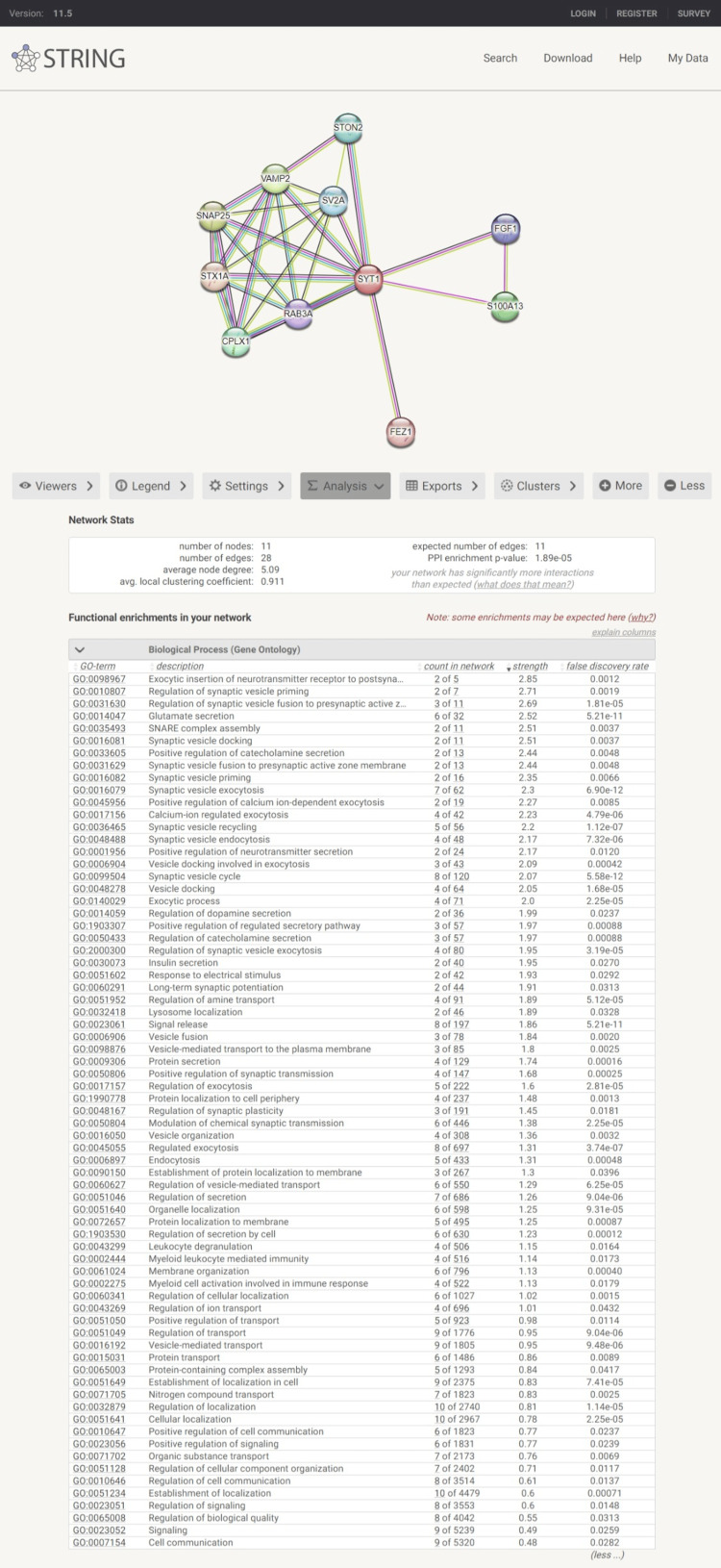
Functional enrichment analysis of SYT1 performed using the STRING database

**Figure 16:**
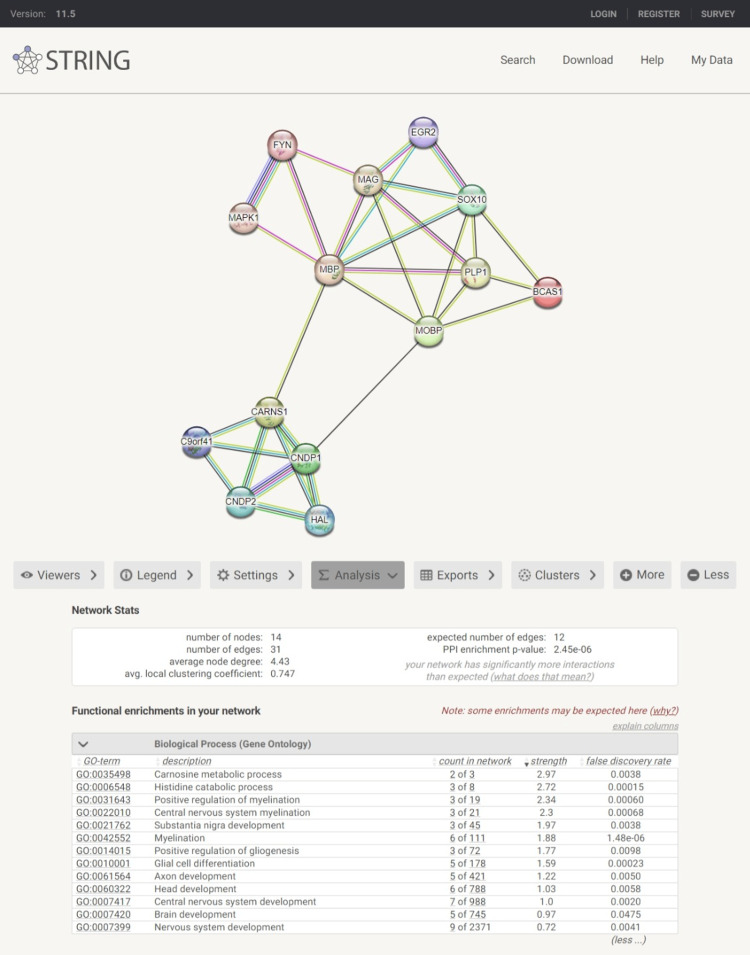
Functional enrichment analysis of BCAS1, MOBP, MBP, and CARNS1 performed using the STRING database

**Figure 17:**
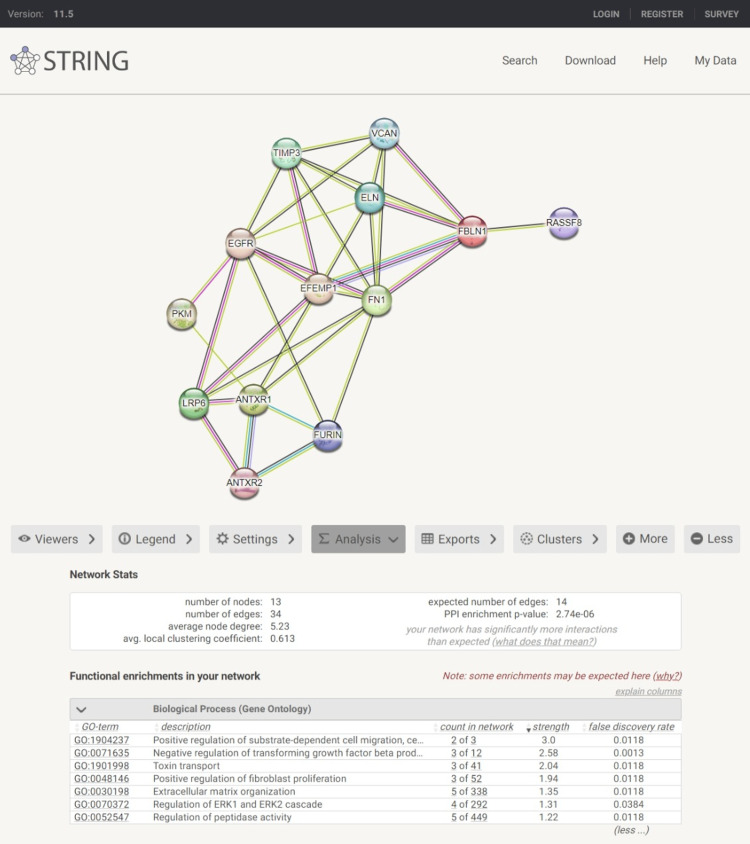
Functional enrichment analysis of FBLN1, EFEMP1, and ANTXR1 performed using the STRING database

**Figure 18:**
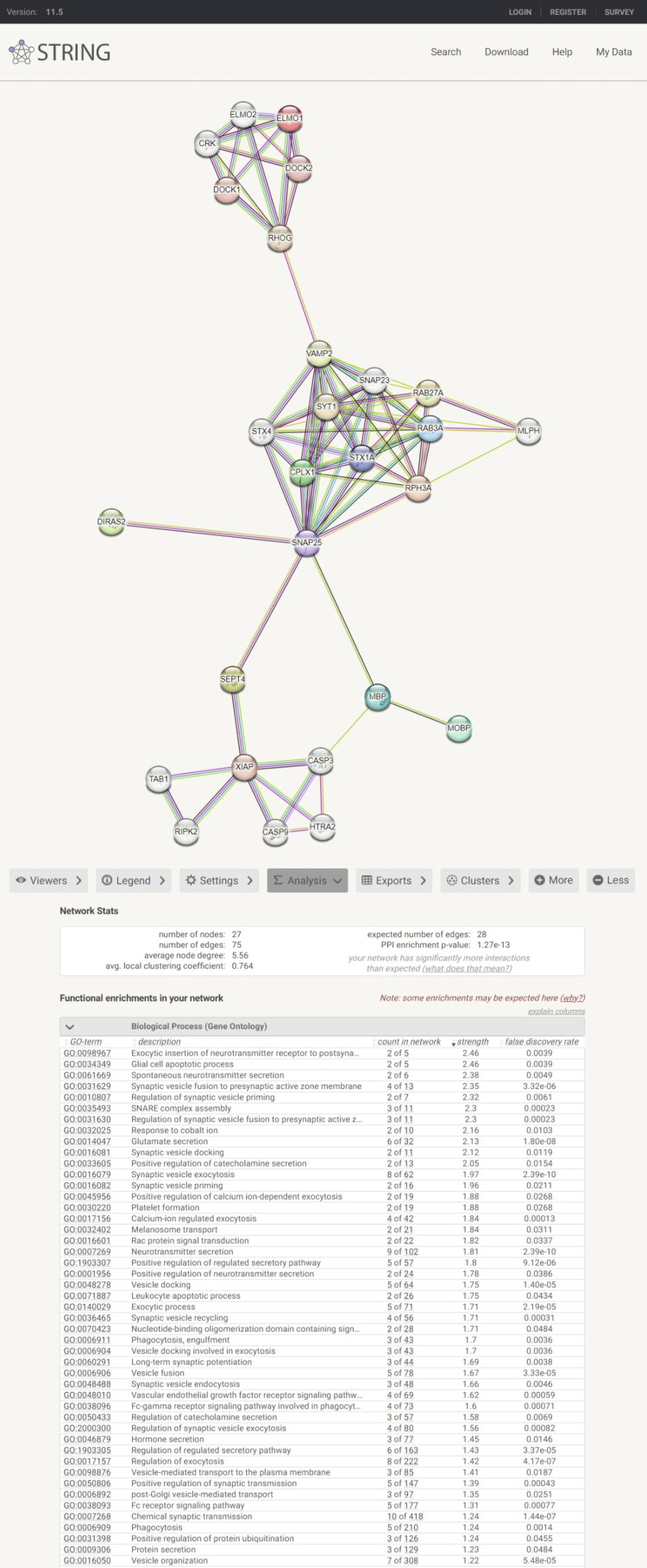
Functional enrichment analysis of DIRAS2, CPLX1, MOBP, MBP, RPH3A, SEPT4, and ELMO1 performed using the STRING database

**Figure 19:**
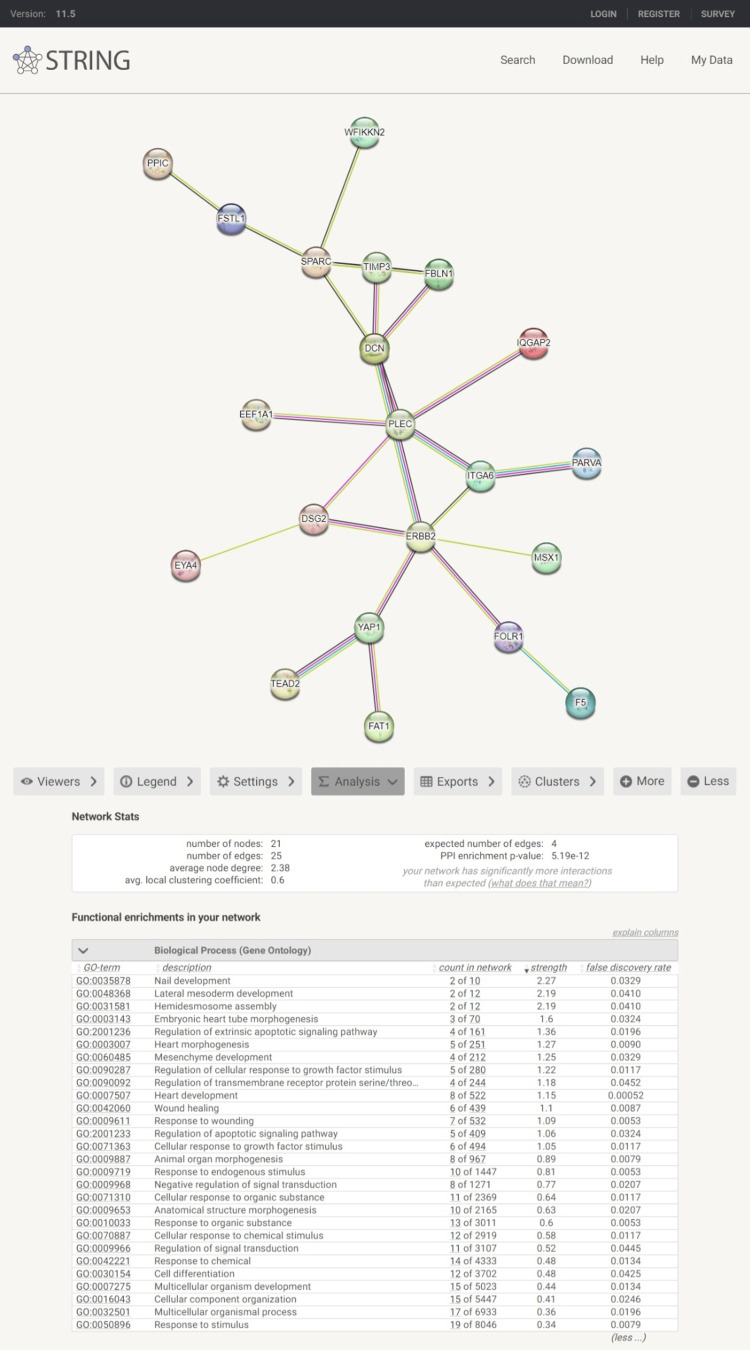
Functional enrichment analysis of FBLN1, FAT1, DCN, FSTL1, DSG2, WFIKKN2, YAP1, IQGAP2, FOLR1, PPIC, ERBB2, MSX1, TIMP3, PLEC, F5, SPARC, EEF1A1, ITGA6, PARVA, EYA4, and TEAD2 performed using the STRING database

## Appendices

Data appendix A

Appendices A-J cited as appendices in the main text of the present review are presented here. These figures demonstrate the results of functional enrichment analysis of proteins performed using the STRING database. Appendices A-F present raw data showing complex interaction networks of protein products of the differentially expressed genes (DEGs) identified in postmortem tissue samples dissected from brain regions of neurologically and neuropathologically normal elderly adults compared to middle-aged adults. Appendices G-J present raw data showing complex interaction networks of protein products of the DEGs identified in postmortem tissue samples dissected from the entorhinal cortex (EC) of patients with Alzheimer’s disease (AD) compared to controls.

Appendix A demonstrates the result of functional enrichment analysis of the protein product of the *PRH1* gene upregulated in the hippocampus of females aged 71 years and older compared to females aged 40-70 years. 

Appendix B demonstrates the result of functional enrichment analysis of the protein product of the HSD11B1 gene upregulated in the cerebellar cortex (CC) of females aged 71 years and over compared to females aged 40-70 years.

Appendix C demonstrates the result of functional enrichment analysis of the protein product of the GABRG1 gene upregulated in the CC of females and males aged 71 years and over compared to females and males aged 40-70 years, respectively.  

Appendix D demonstrates the result of functional enrichment analysis of protein products of IL33, EFEMP1, SLC7A11, GPC5, and HSD17B6 genes downregulated in the putamen of elderly females aged 71 years and over compared to females aged 40-70 years. 

Appendix E demonstrates the result of functional enrichment analysis of the protein product of the GMNC gene upregulated in the white matter (WM) of males aged 71 years and over compared to males aged 40-70 years.

Appendix F demonstrates the result of functional enrichment analysis of the protein product of the SYT1 gene downregulated in the WM of males aged 71 years and over compared to males aged 40-70 years.

Appendix G demonstrates the result of functional enrichment analysis of protein products of BCAS1, MOBP, MBP, and CARNS1 genes downregulated in the EC of female AD patients compared to female controls.

Appendix H demonstrates the result of functional enrichment analysis of protein products of FBLN1, EFEMP1, and ANTXR1 genes upregulated in the EC of female AD patients compared to female controls.

Appendix I demonstrates the result of functional enrichment analysis of protein products of DIRAS2, CPLX1, MOBP, MBP, RPH3A, SEPT4, and ELMO1 genes downregulated in the EC of male AD patients compared to male controls.  

Appendix J demonstrates the result of functional enrichment analysis of protein products of FBLN1, FAT1, DCN, FSTL1, DSG2, WFIKKN2, YAP1, IQGAP2, FOLR1, PPIC, ERBB2, MSX1, TIMP3, PLEC, F5, SPARC, EEF1A1, ITGA6, PARVA, EYA4, and TEAD2 genes upregulated in the EC of male AD patients compared to male controls. 

Data appendix B

The legends of data S1-S25 cited as appendices in the main text of the present review are presented here. Each supplementary data B is stored in an Excel file. The top 250 differentially expressed genes (DEGs) based on adjusted *p*-value identified using the GEO2R tool are included in these Excel files. Excel files demonstrate the results of differential gene expression analysis performed by comparing microarray data between groups from GEO datasets related to postmortem brain tissue samples of neurologically and neuropathologically normal persons and patients with AD, and primary tumor samples from neuroblastoma patients. However, these Excel files cannot be presented as tables due to their huge size. In addition, certain sections of the Excel worksheets from these 25 data files cannot be added as figures due to the limitation of up to 25 media items. Therefore, supplementary data S1-S25 of the present study are available from the corresponding author (N.A.) upon reasonable request. However, the legends of these supplementary data B can be seen below.

Data S1

The results of differential gene expression analysis performed by comparing microarray data of postmortem tissue samples dissected from the frontal cortex of neurologically and neuropathologically normal elderly and middle-aged females. Gene expression data (GSE46706 dataset/GPL5175 platform) of females aged 40-70 years (group 1) and females aged 71 and over (group 2) were compared with each other using the GEO2R tool. An Excel table of the top 250 DEGs based on adjusted *p*-value is presented. 

Data S2

The results of differential gene expression analysis performed by comparing microarray data of postmortem tissue samples dissected from the frontal cortex of neurologically and neuropathologically normal elderly and middle-aged males. Gene expression data (GSE46706 dataset/GPL5175 platform) of males aged 40-70 years (group 1) and males aged 71 and over (group 2) were compared with each other using the GEO2R tool. An Excel table of the top 250 DEGs based on adjusted *p*-value is presented.

Data S3

The results of differential gene expression analysis performed by comparing microarray data of postmortem tissue samples dissected from the occipital cortex of neurologically and neuropathologically normal elderly and middle-aged females. Gene expression data (GSE46706 dataset/GPL5175 platform) of females aged 40-70 years (group 1) and females aged 71 and over (group 2) were compared with each other using the GEO2R tool. An Excel table of the top 250 DEGs based on adjusted *p*-value is presented.

Data S4

The results of differential gene expression analysis performed by comparing microarray data of postmortem tissue samples dissected from the occipital cortex of neurologically and neuropathologically normal elderly and middle-aged males. Gene expression data (GSE46706 dataset/GPL5175 platform) of males aged 40-70 years (group 1) and males aged 71 and over (group 2) were compared with each other using the GEO2R tool. An Excel table of the top 250 DEGs based on adjusted *p*-value is presented.

*Data S5* 

The results of differential gene expression analysis performed by comparing microarray data of postmortem tissue samples dissected from the temporal cortex of neurologically and neuropathologically normal elderly and middle-aged females. Gene expression data (GSE46706 dataset/GPL5175 platform) of females aged 40-70 years (group 1) and females aged 71 and over (group 2) were compared with each other using the GEO2R tool. An Excel table of the top 250 DEGs based on adjusted *p*-value is presented.

Data S6

The results of differential gene expression analysis performed by comparing microarray data of postmortem tissue samples dissected from the temporal cortex of neurologically and neuropathologically normal elderly and middle-aged males. Gene expression data (GSE46706 dataset/GPL5175 platform) of males aged 40-70 years (group 1) and males aged 71 and over (group 2) were compared with each other using the GEO2R tool. An Excel table of the top 250 DEGs based on adjusted *p*-value is presented.

Data S7

The results of differential gene expression analysis performed by comparing microarray data of postmortem tissue samples dissected from the thalamus of neurologically and neuropathologically normal elderly and middle-aged females. Gene expression data (GSE46706 dataset/GPL5175 platform) of females aged 40-70 years (group 1) and females aged 71 and over (group 2) were compared with each other using the GEO2R tool. An Excel table of the top 250 DEGs based on adjusted *p*-value is presented.

Data S8

The results of differential gene expression analysis performed by comparing microarray data of postmortem tissue samples dissected from the thalamus of neurologically and neuropathologically normal elderly and middle-aged males. Gene expression data (GSE46706 dataset/GPL5175 platform) of males aged 40-70 years (group 1) and males aged 71 and over (group 2) were compared with each other using the GEO2R tool. An Excel table of the top 250 DEGs based on adjusted *p*-value is presented.

Data S9

The results of differential gene expression analysis performed by comparing microarray data of postmortem tissue samples dissected from the medulla of neurologically and neuropathologically normal elderly and middle-aged females. Gene expression data (GSE46706 dataset/GPL5175 platform) of females aged 40-70 years (group 1) and females aged 71 and over (group 2) were compared with each other using the GEO2R tool. An Excel table of the top 250 DEGs based on adjusted *p*-value is presented.

Data S10

The results of differential gene expression analysis performed by comparing microarray data of postmortem tissue samples dissected from the medulla of neurologically and neuropathologically normal elderly and middle-aged males. Gene expression data (GSE46706 dataset/GPL5175 platform) of males aged 40-70 years (group 1) and males aged 71 and over (group 2) were compared with each other using the GEO2R tool. An Excel table of the top 250 DEGs based on adjusted *p*-value is presented.

Data S11

The results of differential gene expression analysis performed by comparing microarray data of postmortem tissue samples dissected from the hippocampus of neurologically and neuropathologically normal elderly and middle-aged males. Gene expression data (GSE46706 dataset/GPL5175 platform) of males aged 40-70 years (group 1) and males aged 71 and over (group 2) were compared with each other using the GEO2R tool. An Excel table of the top 250 DEGs based on adjusted *p*-value is presented.

Data S12

The results of differential gene expression analysis performed by comparing microarray data of postmortem tissue samples dissected from the substantia nigra of neurologically and neuropathologically normal elderly and middle-aged males. Gene expression data (GSE46706 dataset/GPL5175 platform) of males aged 40-70 years (group 1) and males aged 71 and over (group 2) were compared with each other using the GEO2R tool. An Excel table of the top 250 DEGs based on adjusted *p*-value is presented.

Data S13

The results of differential gene expression analysis performed by comparing microarray data of postmortem tissue samples dissected from the white matter of neurologically and neuropathologically normal elderly and middle-aged females. Gene expression data (GSE46706 dataset/GPL5175 platform) of females aged 40-70 years (group 1) and females aged 71 and over (group 2) were compared with each other using the GEO2R tool. An Excel table of the top 250 DEGs based on adjusted *p*-value is presented.

Data S14

The results of differential gene expression analysis performed by comparing microarray data of postmortem tissue samples dissected from the cerebellar cortex of neurologically and neuropathologically normal elderly and middle-aged females. Gene expression data (GSE46706 dataset/GPL5175 platform) of females aged 40-70 years (group 1) and females aged 71 and over (group 2) were compared with each other using the GEO2R tool. An Excel table of the top 250 DEGs based on adjusted *p*-value is presented.

Data S15

The results of differential gene expression analysis performed by comparing microarray data of postmortem tissue samples dissected from the cerebellar cortex of neurologically and neuropathologically normal elderly and middle-aged males. Gene expression data (GSE46706 dataset/GPL5175 platform) of males aged 40-70 years (group 1) and males aged 71 and over (group 2) were compared with each other using the GEO2R tool. An Excel table of the top 250 DEGs based on adjusted *p*-value is presented.

Data S16

The results of differential gene expression analysis performed by comparing microarray data of postmortem tissue samples dissected from the hippocampus of neurologically and neuropathologically normal elderly and middle-aged females. Gene expression data (GSE46706 dataset/GPL5175 platform) of females aged 40-70 years (group 1) and females aged 71 and over (group 2) were compared with each other using the GEO2R tool. An Excel table of the top 250 DEGs based on adjusted *p*-value is presented.

Data S17

The results of differential gene expression analysis performed by comparing microarray data of postmortem tissue samples dissected from the putamen of neurologically and neuropathologically normal elderly and middle-aged females. Gene expression data (GSE46706 dataset/GPL5175 platform) of females aged 40-70 years (group 1) and females aged 71 and over (group 2) were compared with each other using the GEO2R tool. An Excel table of the top 250 DEGs based on adjusted *p*-value is presented.

Data S18

The results of differential gene expression analysis performed by comparing microarray data of postmortem tissue samples dissected from the putamen of neurologically and neuropathologically normal elderly and middle-aged males. Gene expression data (GSE46706 dataset/GPL5175 platform) of males aged 40-70 years (group 1) and males aged 71 and over (group 2) were compared with each other using the GEO2R tool. An Excel table of the top 250 DEGs based on adjusted *p*-value is presented.

Data S19

The results of differential gene expression analysis performed by comparing microarray data of postmortem tissue samples dissected from the substantia nigra of neurologically and neuropathologically normal elderly and middle-aged females. Gene expression data (GSE46706 dataset/GPL5175 platform) of females aged 40-70 years (group 1) and females aged 71 and over (group 2) were compared with each other using the GEO2R tool. An Excel table of the top 250 DEGs based on adjusted *p*-value is presented.

Data S20

The results of differential gene expression analysis performed by comparing microarray data of postmortem tissue samples dissected from the white matter of neurologically and neuropathologically normal elderly and middle-aged males. Gene expression data (GSE46706 dataset/GPL5175 platform) of males aged 40-70 years (group 1) and males aged 71 and over (group 2) were compared with each other using the GEO2R tool. An Excel table of the top 250 DEGs based on adjusted *p*-value is presented.

Data S21

The results of differential gene expression analysis performed by comparing microarray transcriptome data (Illumina beadArray platform) of postmortem tissue samples dissected from the entorhinal cortex of female AD patients and female controls. Microarray gene expression data (GSE118553 dataset) of female AD patients with an age range of 61-98 years (group 1) and female controls with an age range of 51-92 years (group 2) were compared with each other using the GEO2R tool. An Excel table of the top 250 DEGs based on adjusted *p*-value is presented.

Data S22

The results of differential gene expression analysis performed by comparing microarray transcriptome data (Illumina beadArray platform) of postmortem tissue samples dissected from the entorhinal cortex of male AD patients and male controls. Microarray gene expression data (GSE118553 dataset) of male AD patients with an age range of 67-105 years (group 1) and male controls with an age range of 65-81 years (group 2) were compared with each other using the GEO2R tool. An Excel table of the top 250 DEGs based on adjusted *p*-value is presented.

Data S23

The results of differential gene expression analysis performed by comparing microarray gene expression data of postmortem tissue samples dissected from the hippocampus of female AD patients and female controls. Microarray gene expression data (GSE48350 dataset) of female AD patients with an age range of 60-91 years (group 1) and female controls with an age range of 64-99 years (group 2) were compared with each other using the GEO2R tool. An Excel table of the top 250 DEGs based on adjusted *p*-value is presented.

Data S24

The results of differential gene expression analysis performed by comparing microarray gene expression data of postmortem tissue samples dissected from the hippocampus of male AD patients and male controls. Microarray gene expression data (GSE48350 dataset) of male AD patients with an age range of 76-94 years (group 1) and male controls with an age range of 75-97 years (group 2) were compared with each other using the GEO2R tool. An Excel table of the top 250 DEGs based on adjusted *p*-value is presented.

Data S25

The results of differential gene expression analysis performed by comparing microarray gene expression data of primary tumor samples from stage 4S neuroblastoma patients and stage 4 neuroblastoma patients. Microarray gene expression data (GSE45547 dataset) of stage 4S neuroblastoma patients (group 1) and stage 4 neuroblastoma patients (group 2) were compared with each other using the GEO2R tool. An Excel table of the top 250 DEGs based on adjusted *p*-value is presented.

## References

[1] Mattson MP, Arumugam TV (2018). Hallmarks of brain aging: adaptive and pathological modification by metabolic states. Cell Metab.

[2] Hou Y, Dan X, Babbar M, Wei Y, Hasselbalch SG, Croteau DL, Bohr VA (2019). Ageing as a risk factor for neurodegenerative disease. Nat Rev Neurol.

[3] Regalado-Santiago C, Juárez-Aguilar E, Olivares-Hernández JD, Tamariz E (2016). Mimicking neural stem cell niche by biocompatible substrates. Stem Cells Int.

[4] Tanaka M, Toldi J, Vécsei L (2020). Exploring the etiological links behind neurodegenerative diseases: inflammatory cytokines and bioactive kynurenines. Int J Mol Sci.

[5] Butler CA, Popescu AS, Kitchener EJ, Allendorf DH, Puigdellívol M, Brown GC (2021). Microglial phagocytosis of neurons in neurodegeneration, and its regulation. J Neurochem.

[6] Bloomingdale P, Karelina T, Ramakrishnan V (2022). Hallmarks of neurodegenerative disease: a systems pharmacology perspective. CPT Pharmacometrics Syst Pharmacol.

[7] Hipp MS, Kasturi P, Hartl FU (2019). The proteostasis network and its decline in ageing. Nat Rev Mol Cell Biol.

[8] Kundra R, Dobson CM, Vendruscolo M (2020). A cell- and tissue-specific weakness of the protein homeostasis system underlies brain vulnerability to protein aggregation. iScience.

[9] Wang H, Kodavati M, Britz GW, Hegde ML (2021). DNA damage and repair deficiency in ALS/FTD-associated neurodegeneration: from molecular mechanisms to therapeutic implication. Front Mol Neurosci.

[10] Michalska P, León R (2020). When it comes to an end: oxidative stress crosstalk with protein aggregation and neuroinflammation induce neurodegeneration. Antioxidants (Basel).

[11] Ghemrawi R, Khair M (2020). Endoplasmic reticulum stress and unfolded protein response in neurodegenerative diseases. Int J Mol Sci.

[12] Medinas DB, Rozas P, Martínez Traub F, Woehlbier U, Brown RH, Bosco DA, Hetz C (2018). Endoplasmic reticulum stress leads to accumulation of wild-type SOD1 aggregates associated with sporadic amyotrophic lateral sclerosis. Proc Natl Acad Sci U S A.

[13] Hamdan N, Kritsiligkou P, Grant CM (2017). ER stress causes widespread protein aggregation and prion formation. J Cell Biol.

[14] Sweeney MD, Sagare AP, Zlokovic BV (2018). Blood-brain barrier breakdown in Alzheimer disease and other neurodegenerative disorders. Nat Rev Neurol.

[15] Riera-Tur I, Schäfer T, Hornburg D (2022). Amyloid-like aggregating proteins cause lysosomal defects in neurons via gain-of-function toxicity. Life Sci Alliance.

[16] Alcendor DJ (2020). Interactions between amyloid-b proteins and human brain pericytes: implications for the pathobiology of Alzheimer’s disease. J Clin Med.

[17] Griffin JW, Bradshaw PC (2017). Amino acid catabolism in Alzheimer’s disease brain: friend or foe?. Oxid Med Cell Longev.

[18] Jadiya P, Garbincius JF, Elrod JW (2021). Reappraisal of metabolic dysfunction in neurodegeneration: focus on mitochondrial function and calcium signaling. Acta Neuropathol Commun.

[19] Wyttenbach A, Carmichael J, Swartz J, Furlong RA, Narain Y, Rankin J, Rubinsztein DC (2000). Effects of heat shock, heat shock protein 40 (HDJ-2), and proteasome inhibition on protein aggregation in cellular models of Huntington's disease. Proc Natl Acad Sci U S A.

[20] Piazzon N, Rage F, Schlotter F, Moine H, Branlant C, Massenet S (2008). In vitro and in cellulo evidences for association of the survival of motor neuron complex with the fragile X mental retardation protein. J Biol Chem.

[21] Martínez-Pinilla E, Rubio-Sardón N, Villar-Conde S (2021). Cuprizone-induced neurotoxicity in human neural cell lines is mediated by a reversible mitochondrial dysfunction: relevance for demyelination models. Brain Sci.

[22] Rey F, Marcuzzo S, Bonanno S (2021). LncRNAs associated with neuronal development and oncogenesis are deregulated in SOD1-G93A murine model of amyotrophic lateral sclerosis. Biomedicines.

[23] Cetin S, Knez D, Gobec S, Kos J, Pišlar A (2022). Cell models for Alzheimer's and Parkinson's disease: at the interface of biology and drug discovery. Biomed Pharmacother.

[24] Bell M, Zempel H (2022). SH-SY5Y-derived neurons: a human neuronal model system for investigating TAU sorting and neuronal subtype-specific TAU vulnerability. Rev Neurosci.

[25] Yang Y, Herrup K (2007). Cell division in the CNS: protective response or lethal event in post-mitotic neurons?. Biochim Biophys Acta.

[26] Ghosh HS (2019). Adult neurogenesis and the promise of adult neural stem cells. J Exp Neurosci.

[27] Isaev NK, Stelmashook EV, Genrikhs EE (2019). Neurogenesis and brain aging. Rev Neurosci.

[28] Walton CC, Zhang W, Patiño-Parrado I, Barrio-Alonso E, Garrido JJ, Frade JM (2019). Primary neurons can enter M-phase. Sci Rep.

[29] Terreros-Roncal J, Moreno-Jiménez EP, Flor-García M (2021). Impact of neurodegenerative diseases on human adult hippocampal neurogenesis. Science.

[30] Prusiner SB (2001). Shattuck lecture--neurodegenerative diseases and prions. N Engl J Med.

[31] Peng C, Trojanowski JQ, Lee VM (2020). Protein transmission in neurodegenerative disease. Nat Rev Neurol.

[32] Andreone BJ, Larhammar M, Lewcock JW (2020). Cell death and neurodegeneration. Cold Spring Harb Perspect Biol.

[33] Moujalled D, Strasser A, Liddell JR (2021). Molecular mechanisms of cell death in neurological diseases. Cell Death Differ.

[34] Schulte JH, Lindner S, Bohrer A (2013). MYCN and ALKF1174L are sufficient to drive neuroblastoma development from neural crest progenitor cells. Oncogene.

[35] Jansky S, Sharma AK, Körber V (2021). Single-cell transcriptomic analyses provide insights into the developmental origins of neuroblastoma. Nat Genet.

[36] Bown N (2001). Neuroblastoma tumour genetics: clinical and biological aspects. J Clin Pathol.

[37] Aygun N (2018). Biological and genetic features of neuroblastoma and their clinical importance. Curr Pediatr Rev.

[38] Li F, Zhang W, Hu H, Zhang Y, Li J, Huang D (2022). Factors of recurrence after complete response in children with neuroblastoma: a 16-year retrospective study of 179 cases. Cancer Manag Res.

[39] Thomas CG, Spyrou G (2009). ERdj5 sensitizes neuroblastoma cells to endoplasmic reticulum stress-induced apoptosis. J Biol Chem.

[40] París-Coderch L, Soriano A, Jiménez C (2020). The antitumour drug ABTL0812 impairs neuroblastoma growth through endoplasmic reticulum stress-mediated autophagy and apoptosis. Cell Death Dis.

[41] Ogata M, Hino S, Saito A (2006). Autophagy is activated for cell survival after endoplasmic reticulum stress. Mol Cell Biol.

[42] Belounis A, Nyalendo C, Le Gall R (2016). Autophagy is associated with chemoresistance in neuroblastoma. BMC Cancer.

[43] Arai K, Lee SR, van Leyen K, Kurose H, Lo EH (2004). Involvement of ERK MAP kinase in endoplasmic reticulum stress in SH-SY5Y human neuroblastoma cells. J Neurochem.

[44] Berchtold NC, Cribbs DH, Coleman PD (2008). Gene expression changes in the course of normal brain aging are sexually dimorphic. Proc Natl Acad Sci U S A.

[45] Barrett T, Wilhite SE, Ledoux P (2013). NCBI GEO: archive for functional genomics data sets--update. Nucleic Acids Res.

[46] Trabzuni D, Ramasamy A, Imran S (2013). Widespread sex differences in gene expression and splicing in the adult human brain. Nat Commun.

[47] Ramasamy A, Trabzuni D, Guelfi S (2014). Genetic variability in the regulation of gene expression in ten regions of the human brain. Nat Neurosci.

[48] NCBI GEO (2022). NCBI GEO: GEO DataSets, Gene Expression Omnibus (GEO) Datasets. https://www.ncbi.nlm.nih.gov/gds.

[49] (2022). NCBI Gene: Home - Gene - National Center for Biotechnology Information (NCBI). https://www.ncbi.nlm.nih.gov/gene/.

[50] Carlson DM (1993). Salivary proline-rich proteins: biochemistry, molecular biology, and regulation of expression. Crit Rev Oral Biol Med.

[51] (2023). UniProt: UniProt database-protein function. https://www.uniprot.org/.

[52] (2022). HPA: The Human Protein Atlas: the open access resource for human proteins. https://www.proteinatlas.org/.

[53] Szklarczyk D, Gable AL, Nastou KC (2021). The STRING database in 2021: customizable protein-protein networks, and functional characterization of user-uploaded gene/measurement sets. Nucleic Acids Res.

[54] (2022). STRING database: STRING: functional protein association networks. https://string-db.org/cgi/input?sessionId=bWs20SlIn691&input_page_show_search=on.

[55] Sapolsky RM, Krey LC, McEwen BS (1985). Prolonged glucocorticoid exposure reduces hippocampal neuron number: implications for aging. J Neurosci.

[56] Sorrells SF, Sapolsky RM (2007). An inflammatory review of glucocorticoid actions in the CNS. Brain Behav Immun.

[57] Gruver-Yates AL, Cidlowski JA (2013). Tissue-specific actions of glucocorticoids on apoptosis: a double-edged sword. Cells.

[58] Duque Ede A, Munhoz CD (2016). The pro-inflammatory effects of glucocorticoids in the brain. Front Endocrinol (Lausanne).

[59] Zhang B, Zhang Y, Wu W (2017). Chronic glucocorticoid exposure activates BK-NLRP1 signal involving in hippocampal neuron damage. J Neuroinflammation.

[60] Adhya D, Annuario E, Lancaster MA, Price J, Baron-Cohen S, Srivastava DP (2018). Understanding the role of steroids in typical and atypical brain development: advantages of using a "brain in a dish" approach. J Neuroendocrinol.

[61] Liberman AC, Budziñski ML, Sokn C, Gobbini RP, Steininger A, Arzt E (2018). Regulatory and mechanistic actions of glucocorticoids on T and inflammatory cells. Front Endocrinol (Lausanne).

[62] Gerber AN, Newton R, Sasse SK (2021). Repression of transcription by the glucocorticoid receptor: a parsimonious model for the genomics era. J Biol Chem.

[63] Xia P, Liu Y, Chen J, Cheng Z (2019). Cell cycle proteins as key regulators of postmitotic cell death. Yale J Biol Med.

[64] Frade JM, Ovejero-Benito MC (2015). Neuronal cell cycle: the neuron itself and its circumstances. Cell Cycle.

[65] López-Sánchez N, Fontán-Lozano Á, Pallé A, González-Álvarez V, Rábano A, Trejo JL, Frade JM (2017). Neuronal tetraploidization in the cerebral cortex correlates with reduced cognition in mice and precedes and recapitulates Alzheimer's-associated neuropathology. Neurobiol Aging.

[66] Zhao XS, Wu Q, Peng J (2017). Hyperlipidemia-induced apoptosis of hippocampal neurons in apoE(-/-) mice may be associated with increased PCSK9 expression. Mol Med Rep.

[67] Van Essen DC, Donahue CJ, Glasser MF (2018). Development and evolution of cerebral and cerebellar cortex. Brain Behav Evol.

[68] Tam WY, Wang X, Cheng AS, Cheung KK (2021). In search of molecular markers for cerebellar neurons. Int J Mol Sci.

[69] Amore G, Spoto G, Ieni A, Vetri L, Quatrosi G, Di Rosa G, Nicotera AG (2021). A focus on the cerebellum: from embryogenesis to an age-related clinical perspective. Front Syst Neurosci.

[70] Gellersen HM, Guo CC, O'Callaghan C, Tan RH, Sami S, Hornberger M (2017). Cerebellar atrophy in neurodegeneration-a meta-analysis. J Neurol Neurosurg Psychiatry.

[71] Sandeep TC, Yau JL, MacLullich AM, Noble J, Deary IJ, Walker BR, Seckl JR (2004). 11Beta-hydroxysteroid dehydrogenase inhibition improves cognitive function in healthy elderly men and type 2 diabetics. Proc Natl Acad Sci U S A.

[72] Moisan MP, Seckl JR, Edwards CR (1990). 11 beta-hydroxysteroid dehydrogenase bioactivity and messenger RNA expression in rat forebrain: localization in hypothalamus, hippocampus, and cortex. Endocrinology.

[73] Wyrwoll CS, Holmes MC, Seckl JR (2011). 11β-hydroxysteroid dehydrogenases and the brain: from zero to hero, a decade of progress. Front Neuroendocrinol.

[74] Holmes MC, Carter RN, Noble J (2010). 11beta-hydroxysteroid dehydrogenase type 1 expression is increased in the aged mouse hippocampus and parietal cortex and causes memory impairments. J Neurosci.

[75] Yau JL, Seckl JR (2012). Local amplification of glucocorticoids in the aging brain and impaired spatial memory. Front Aging Neurosci.

[76] Schouten M, Bielefeld P, Garcia-Corzo L (2020). Circadian glucocorticoid oscillations preserve a population of adult hippocampal neural stem cells in the aging brain. Mol Psychiatry.

[77] Noguchi KK, Walls KC, Wozniak DF, Olney JW, Roth KA, Farber NB (2008). Acute neonatal glucocorticoid exposure produces selective and rapid cerebellar neural progenitor cell apoptotic death. Cell Death Differ.

[78] Harlé G, Lalonde R, Fonte C, Ropars A, Frippiat JP, Strazielle C (2017). Repeated corticosterone injections in adult mice alter stress hormonal receptor expression in the cerebellum and motor coordination without affecting spatial learning. Behav Brain Res.

[79] Morimoto M, Morita N, Ozawa H, Yokoyama K, Kawata M (1996). Distribution of glucocorticoid receptor immunoreactivity and mRNA in the rat brain: an immunohistochemical and in situ hybridization study. Neurosci Res.

[80] Swinny JD, Metzger F, IJkema-Paassen J, Gounko NV, Gramsbergen A, van der Want JJ (2004). Corticotropin-releasing factor and urocortin differentially modulate rat Purkinje cell dendritic outgrowth and differentiation in vitro. Eur J Neurosci.

[81] Choi JS, Pham TT, Jang YJ (2006). Corticotropin-releasing factor (CRF) and urocortin promote the survival of cultured cerebellar GABAergic neurons through the type 1 CRF receptor. J Korean Med Sci.

[82] Price RH Jr, Handa RJ (2000). Expression of estrogen receptor-beta protein and mRNA in the cerebellum of the rat. Neurosci Lett.

[83] Andreescu CE, Milojkovic BA, Haasdijk ED (2007). Estradiol improves cerebellar memory formation by activating estrogen receptor beta. J Neurosci.

[84] Davis SR, Bell RJ, Robinson PJ (2019). Testosterone and estrone increase from the age of 70 years: findings from the sex hormones in older women study. J Clin Endocrinol Metab.

[85] Moffat SD, An Y, Resnick SM, Diamond MP, Ferrucci L (2020). Longitudinal change in cortisol levels across the adult life span. J Gerontol A Biol Sci Med Sci.

[86] Woodruff-Pak DS, Foy MR, Akopian GG (2010). Differential effects and rates of normal aging in cerebellum and hippocampus. Proc Natl Acad Sci U S A.

[87] Zhu Y, Lee CC, Lam WP, Wai MS, Rudd JA, Yew DT (2007). Cell death in the Purkinje cells of the cerebellum of senescence accelerated mouse (SAMP(8)). Biogerontology.

[88] Wang DD, Kriegstein AR (2009). Defining the role of GABA in cortical development. J Physiol.

[89] Rissman RA, De Blas AL, Armstrong DM (2007). GABA(A) receptors in aging and Alzheimer's disease. J Neurochem.

[90] Rozycka A, Liguz-Lecznar M (2017). The space where aging acts: focus on the GABAergic synapse. Aging Cell.

[91] Ghit A, Assal D, Al-Shami AS, Hussein DE (2021). GABA(A) receptors: structure, function, pharmacology, and related disorders. J Genet Eng Biotechnol.

[92] Vela J, Gutierrez A, Vitorica J, Ruano D (2003). Rat hippocampal GABAergic molecular markers are differentially affected by ageing. J Neurochem.

[93] Aman Y, Schmauck-Medina T, Hansen M (2021). Autophagy in healthy aging and disease. Nat Aging.

[94] Gómez-Virgilio L, Silva-Lucero MD, Flores-Morelos DS (2022). Autophagy: a key regulator of homeostasis and disease: an overview of molecular mechanisms and modulators. Cells.

[95] Hui KK, Tanaka M (2019). Autophagy links MTOR and GABA signaling in the brain. Autophagy.

[96] Hui KK, Takashima N, Watanabe A (2019). GABARAPs dysfunction by autophagy deficiency in adolescent brain impairs GABA(A) receptor trafficking and social behavior. Sci Adv.

[97] Lakhani R, Vogel KR, Till A, Liu J, Burnett SF, Gibson KM, Subramani S (2014). Defects in GABA metabolism affect selective autophagy pathways and are alleviated by mTOR inhibition. EMBO Mol Med.

[98] Sato M, Ohta T, Morikawa Y (2021). Ataxic phenotype and neurodegeneration are triggered by the impairment of chaperone-mediated autophagy in cerebellar neurons. Neuropathol Appl Neurobiol.

[99] Chakrabarti L, Eng J, Ivanov N, Garden GA, La Spada AR (2009). Autophagy activation and enhanced mitophagy characterize the Purkinje cells of pcd mice prior to neuronal death. Mol Brain.

[100] Hirano T (2013). GABA and Synaptic Transmission in the Cerebellum. Handbook of the cerebellum and cerebellar disorders.

[101] Das S, Kohr M, Dunkerly-Eyring B (2017). Divergent effects of miR-181 family members on myocardial function through protective cytosolic and detrimental mitochondrial microRNA targets. J Am Heart Assoc.

[102] (2022). GeneCards: the human gene database. https://www.genecards.org/.

[103] Li JG, Ding Y, Huang YM (2017). FAMLF is a target of miR-181b in Burkitt lymphoma. Braz J Med Biol Res.

[104] Huang YM, Zheng Y, Li JG (2017). Lentivirus-mediated RNA interference targeting FAMLF-1 inhibits cell growth and enhances cell differentiation of acute myeloid leukemia partially differentiated cells via inhibition of AKT and c-MYC. Oncotarget.

[105] Zhang SF, Chen JC, Zhang J, Xu JG (2017). miR-181a involves in the hippocampus-dependent memory formation via targeting PRKAA1. Sci Rep.

[106] Fastenrath M, Spalek K, Coynel D (2022). Human cerebellum and corticocerebellar connections involved in emotional memory enhancement. Proc Natl Acad Sci U S A.

[107] Xu XF, Jing X, Ma HX (2018). miR-181a participates in contextual fear memory formation via activating mTOR signaling pathway. Cereb Cortex.

[108] Frontera JL, Baba Aissa H, Sala RW, Mailhes-Hamon C, Georgescu IA, Léna C, Popa D (2020). Bidirectional control of fear memories by cerebellar neurons projecting to the ventrolateral periaqueductal grey. Nat Commun.

[109] Luo Y, Zhou J, Li MX (2015). Reversal of aging-related emotional memory deficits by norepinephrine via regulating the stability of surface AMPA receptors. Aging Cell.

[110] Carmona V, Cunha-Santos J, Onofre I, Simões AT, Vijayakumar U, Davidson BL, Pereira de Almeida L (2017). Unravelling endogenous microRNA system dysfunction as a new pathophysiological mechanism in Machado-Joseph disease. Mol Ther.

[111] Freeman SH, Kandel R, Cruz L (2008). Preservation of neuronal number despite age-related cortical brain atrophy in elderly subjects without Alzheimer disease. J Neuropathol Exp Neurol.

[112] Andersen BB, Gundersen HJ, Pakkenberg B (2003). Aging of the human cerebellum: a stereological study. J Comp Neurol.

[113] Nishiyama J, Miura E, Mizushima N, Watanabe M, Yuzaki M (2007). Aberrant membranes and double-membrane structures accumulate in the axons of Atg5-null Purkinje cells before neuronal death. Autophagy.

[114] Schaefer A, O'Carroll D, Tan CL, Hillman D, Sugimori M, Llinas R, Greengard P (2007). Cerebellar neurodegeneration in the absence of microRNAs. J Exp Med.

[115] Liu Y, Song Y, Zhu X (2017). MicroRNA-181a regulates apoptosis and autophagy process in Parkinson’s disease by inhibiting p38 mitogen-activated protein kinase (MAPK)/c-Jun N-terminal kinases (JNK) signaling pathways. Med Sci Monit.

[116] Ye Y, He X, Lu F (2018). A lincRNA-p21/miR-181 family feedback loop regulates microglial activation during systemic LPS- and MPTP- induced neuroinflammation. Cell Death Dis.

[117] Lyu X, Li J, Yun X (2017). miR-181a-5p, an inducer of Wnt-signaling, facilitates cell proliferation in acute lymphoblastic leukemia. Oncol Rep.

[118] Ji J, Yamashita T, Wang XW (2011). Wnt/beta-catenin signaling activates microRNA-181 expression in hepatocellular carcinoma. Cell Biosci.

[119] Santiago L, Daniels G, Wang D, Deng FM, Lee P (2017). Wnt signaling pathway protein LEF1 in cancer, as a biomarker for prognosis and a target for treatment. Am J Cancer Res.

[120] Wu C, Bendriem RM, Freed WJ, Lee CT (2021). Transcriptome analysis of human dorsal striatum implicates attenuated canonical WNT signaling in neuroinflammation and in age-related impairment of striatal neurogenesis and synaptic plasticity. Restor Neurol Neurosci.

[121] Xie W, Li M, Xu N, Lv Q, Huang N, He J, Zhang Y (2013). MiR-181a regulates inflammation responses in monocytes and macrophages. PLoS One.

[122] Nunnemann S, Wohlschläger AM, Ilg R (2009). Accelerated aging of the putamen in men but not in women. Neurobiol Aging.

[123] Wang S, Qiu M, Xia W (2016). Glypican-5 suppresses epithelial-mesenchymal transition of the lung adenocarcinoma by competitively binding to Wnt3a. Oncotarget.

[124] Yuan S, Yu Z, Liu Q (2016). GPC5, a novel epigenetically silenced tumor suppressor, inhibits tumor growth by suppressing Wnt/β-catenin signaling in lung adenocarcinoma. Oncogene.

[125] Rao X, Hua F, Zhang L (2022). Dual roles of interleukin-33 in cognitive function by regulating central nervous system inflammation. J Transl Med.

[126] Fearnley JM, Lees AJ (1991). Ageing and Parkinson's disease: substantia nigra regional selectivity. Brain.

[127] Noda S, Sato S, Fukuda T, Tada N, Hattori N (2020). Aging-related motor function and dopaminergic neuronal loss in C57BL/6 mice. Mol Brain.

[128] Wood-Kaczmar A, Gandhi S, Yao Z (2008). PINK1 is necessary for long term survival and mitochondrial function in human dopaminergic neurons. PLoS One.

[129] Lee YI, Kang H, Ha YW (2016). Diaminodiphenyl sulfone-induced parkin ameliorates age-dependent dopaminergic neuronal loss. Neurobiol Aging.

[130] Ham SJ, Lee D, Yoo H, Jun K, Shin H, Chung J (2020). Decision between mitophagy and apoptosis by Parkin via VDAC1 ubiquitination. Proc Natl Acad Sci U S A.

[131] Lewis MM, Du G, Lee EY (2016). The pattern of gray matter atrophy in Parkinson's disease differs in cortical and subcortical regions. J Neurol.

[132] Moisan F, Kab S, Mohamed F (2016). Parkinson disease male-to-female ratios increase with age: French nationwide study and meta-analysis. J Neurol Neurosurg Psychiatry.

[133] Zhang Y, Marmorstein LY (2010). Focus on molecules: fibulin-3 (EFEMP1). Exp Eye Res.

[134] Zhang N, Liao Z, Wu P, Fang H, Cai G (2020). Hypermethylation of EFEMP1 in the hippocampus may be related to the deficit in spatial memory of rat neonates triggered by repeated administration of propofol. Biomed Res Int.

[135] Salvesen L, Ullerup BH, Sunay FB (2015). Changes in total cell numbers of the basal ganglia in patients with multiple system atrophy - a stereological study. Neurobiol Dis.

[136] Gadani SP, Walsh JT, Smirnov I, Zheng J, Kipnis J (2015). The glia-derived alarmin IL-33 orchestrates the immune response and promotes recovery following CNS injury. Neuron.

[137] Kempuraj D, Thangavel R, Selvakumar GP (2019). Mast cell proteases activate astrocytes and glia-neurons and release interleukin-33 by activating p38 and ERK1/2 MAPKs and NF-κB. Mol Neurobiol.

[138] Koppula P, Zhang Y, Zhuang L, Gan B (2018). Amino acid transporter SLC7A11/xCT at the crossroads of regulating redox homeostasis and nutrient dependency of cancer. Cancer Commun (Lond).

[139] Wang W, Shi F, Cui J, Pang S, Zheng G, Zhang Y (2022). MiR-378a-3p/ SLC7A11 regulate ferroptosis in nerve injury induced by lead exposure. Ecotoxicol Environ Saf.

[140] Galli S, Lopes DM, Ammari R, Kopra J, Millar SE, Gibb A, Salinas PC (2014). Deficient Wnt signalling triggers striatal synaptic degeneration and impaired motor behaviour in adult mice. Nat Commun.

[141] Provost JS, Hanganu A, Monchi O (2015). Neuroimaging studies of the striatum in cognition Part I: healthy individuals. Front Syst Neurosci.

[142] Fukuda M (2002). Vesicle-associated membrane protein-2/synaptobrevin binding to synaptotagmin I promotes O-glycosylation of synaptotagmin I. J Biol Chem.

[143] Ullah N, Maaiden EE, Uddin MS, Ashraf GM (2021). Synaptotagmin- 1: a multi-functional protein that mediates vesicle docking, priming, and fusion. Curr Protein Pept Sci.

[144] Riggs E, Shakkour Z, Anderson CL, Carney PR (2022). SYT1-associated neurodevelopmental disorder: a narrative review. Children (Basel).

[145] Stahon KE, Bastian C, Griffith S, Kidd GJ, Brunet S, Baltan S (2016). Age-related changes in axonal and mitochondrial ultrastructure and function in white matter. J Neurosci.

[146] Ali Moussa HY, Shin KC, Ponraj J, Kim SJ, Ryu JK, Mansour S, Park Y (2023). Requirement of cholesterol for calcium-dependent vesicle fusion by strengthening synaptotagmin-1-induced membrane bending. Adv Sci (Weinh).

[147] Egawa J, Pearn ML, Lemkuil BP, Patel PM, Head BP (2016). Membrane lipid rafts and neurobiology: age-related changes in membrane lipids and loss of neuronal function. J Physiol.

[148] Coelho A, Fernandes HM, Magalhães R (2021). Signatures of white-matter microstructure degradation during aging and its association with cognitive status. Sci Rep.

[149] Cui J, Zhao S, Li Y, Zhang D, Wang B, Xie J, Wang J (2021). Regulated cell death: discovery, features and implications for neurodegenerative diseases. Cell Commun Signal.

[150] Fricker M, Tolkovsky AM, Borutaite V, Coleman M, Brown GC (2018). Neuronal cell death. Physiol Rev.

[151] Ramos Bernardes da Silva Filho S, Oliveira Barbosa JH, Rondinoni C (2017). Neuro-degeneration profile of Alzheimer's patients: a brain morphometry study. Neuroimage Clin.

[152] Jucker M, Walker LC (2011). Pathogenic protein seeding in Alzheimer disease and other neurodegenerative disorders. Ann Neurol.

[153] Grothe MJ, Barthel H, Sepulcre J, Dyrba M, Sabri O, Teipel SJ (2017). In vivo staging of regional amyloid deposition. Neurology.

[154] Braak H, Braak E (1991). Neuropathological stageing of Alzheimer-related changes. Acta Neuropathol.

[155] Berron D, Vogel JW, Insel PS (2021). Early stages of tau pathology and its associations with functional connectivity, atrophy and memory. Brain.

[156] Bloom GS (2014). Amyloid-β and tau: the trigger and bullet in Alzheimer disease pathogenesis. JAMA Neurol.

[157] Kametani F, Hasegawa M (2018). Reconsideration of amyloid hypothesis and tau hypothesis in Alzheimer’s disease. Front Neurosci.

[158] Dunning CJ, McGauran G, Willén K, Gouras GK, O'Connell DJ, Linse S (2016). Direct high affinity interaction between Aβ42 and GSK3α stimulates hyperphosphorylation of tau. A new molecular link in Alzheimer’s disease?. ACS Chem Neurosci.

[159] Kummer MP, Ising C, Kummer C (2021). Microglial PD-1 stimulation by astrocytic PD-L1 suppresses neuroinflammation and Alzheimer's disease pathology. EMBO J.

[160] Hickman SE, Allison EK, El Khoury J (2008). Microglial dysfunction and defective beta-amyloid clearance pathways in aging Alzheimer's disease mice. J Neurosci.

[161] Tan MS, Tan L, Jiang T, Zhu XC, Wang HF, Jia CD, Yu JT (2014). Amyloid-β induces NLRP1-dependent neuronal pyroptosis in models of Alzheimer's disease. Cell Death Dis.

[162] Salvadores N, Moreno-Gonzalez I, Gamez N (2022). Aβ oligomers trigger necroptosis-mediated neurodegeneration via microglia activation in Alzheimer's disease. Acta Neuropathol Commun.

[163] Saha A, Saleem S, Paidi RK, Biswas SC (2021). BH3-only proteins Puma and Beclin1 regulate autophagic death in neurons in response to Amyloid-β. Cell Death Discov.

[164] Li MZ, Liu EJ, Zhou QZ (2022). Intracellular accumulation of tau inhibits autophagosome formation by activating TIA1-amino acid-mTORC1 signaling. Mil Med Res.

[165] Hu Y, Li XC, Wang ZH (2016). Tau accumulation impairs mitophagy via increasing mitochondrial membrane potential and reducing mitochondrial Parkin. Oncotarget.

[166] Wong YC, Luk K, Purtell K (2019). Neuronal vulnerability in Parkinson disease: should the focus be on axons and synaptic terminals?. Mov Disord.

[167] Pan L, Meng L, He M, Zhang Z (2021). Tau in the pathophysiology of Parkinson’s disease. J Mol Neurosci.

[168] Compta Y, Parkkinen L, O'Sullivan SS (2011). Lewy- and Alzheimer-type pathologies in Parkinson's disease dementia: which is more important?. Brain.

[169] Tran J, Anastacio H, Bardy C (2020). Genetic predispositions of Parkinson's disease revealed in patient-derived brain cells. NPJ Parkinsons Dis.

[170] Chai C, Lim KL (2013). Genetic insights into sporadic Parkinson's disease pathogenesis. Curr Genomics.

[171] Hartmann A, Troadec JD, Hunot S (2001). Caspase-8 is an effector in apoptotic death of dopaminergic neurons in Parkinson's disease, but pathway inhibition results in neuronal necrosis. J Neurosci.

[172] Zhang Q, Hu C, Huang J (2019). ROCK1 induces dopaminergic nerve cell apoptosis via the activation of Drp1-mediated aberrant mitochondrial fission in Parkinson's disease. Exp Mol Med.

[173] Ganjam GK, Bolte K, Matschke LA (2019). Mitochondrial damage by α-synuclein causes cell death in human dopaminergic neurons. Cell Death Dis.

[174] Zhang X, Zhang Y, Li R, Zhu L, Fu B, Yan T (2020). Salidroside ameliorates Parkinson's disease by inhibiting NLRP3-dependent pyroptosis. Aging (Albany NY).

[175] Wang W, Nguyen LT, Burlak C (2016). Caspase-1 causes truncation and aggregation of the Parkinson's disease-associated protein α-synuclein. Proc Natl Acad Sci U S A.

[176] Tang Q, Gao P, Arzberger T, Höllerhage M, Herms J, Höglinger G, Koeglsperger T (2021). Alpha-synuclein defects autophagy by impairing SNAP29-mediated autophagosome-lysosome fusion. Cell Death Dis.

[177] Decressac M, Mattsson B, Weikop P, Lundblad M, Jakobsson J, Björklund A (2013). TFEB-mediated autophagy rescues midbrain dopamine neurons from α-synuclein toxicity. Proc Natl Acad Sci U S A.

[178] Shaltouki A, Hsieh CH, Kim MJ, Wang X (2018). Alpha-synuclein delays mitophagy and targeting Miro rescues neuron loss in Parkinson's models. Acta Neuropathol.

[179] Eberhardt EL, Ludlam AV, Tan Z, Cianfrocco MA (2020). Miro: a molecular switch at the center of mitochondrial regulation. Protein Sci.

[180] Wilkaniec A, Lenkiewicz AM, Babiec L (2021). Exogenous alpha-synuclein evoked Parkin downregulation promotes mitochondrial dysfunction in neuronal cells. Implications for Parkinson’s disease pathology. Front Aging Neurosci.

[181] Creed RB, Goldberg MS (2020). Enhanced susceptibility of PINK1 knockout rats to α-synuclein fibrils. Neuroscience.

[182] Outeiro TF, Koss DJ, Erskine D (2019). Dementia with Lewy bodies: an update and outlook. Mol Neurodegener.

[183] Hansen D, Ling H, Lashley T, Holton JL, Warner TT (2019). Review: clinical, neuropathological and genetic features of Lewy body dementias. Neuropathol Appl Neurobiol.

[184] Khadhraoui E, Müller SJ, Hansen N (2022). Manual and automated analysis of atrophy patterns in dementia with Lewy bodies on MRI. BMC Neurol.

[185] Aarsland D, Batzu L, Halliday GM, Geurtsen GJ, Ballard C, Ray Chaudhuri K, Weintraub D (2021). Parkinson disease-associated cognitive impairment. Nat Rev Dis Primers.

[186] Gomperts SN, Locascio JJ, Makaretz SJ (2016). Tau positron emission tomographic imaging in the Lewy body diseases. JAMA Neurol.

[187] Guerreiro R, Ross OA, Kun-Rodrigues C (2018). Investigating the genetic architecture of dementia with Lewy bodies: a two-stage genome-wide association study. Lancet Neurol.

[188] Orme T, Guerreiro R, Bras J (2018). The genetics of dementia with Lewy bodies: current understanding and future directions. Curr Neurol Neurosci Rep.

[189] Keogh MJ, Kurzawa-Akanbi M, Griffin H (2016). Exome sequencing in dementia with Lewy bodies. Transl Psychiatry.

[190] Vergouw LJ, Bosman B, van de Beek M (2020). Family history is associated with phenotype in dementia with Lewy bodies. J Alzheimers Dis.

[191] Smolders S, Philtjens S, Crosiers D (2021). Contribution of rare homozygous and compound heterozygous VPS13C missense mutations to dementia with Lewy bodies and Parkinson's disease. Acta Neuropathol Commun.

[192] Kramer ML, Schulz-Schaeffer WJ (2007). Presynaptic alpha-synuclein aggregates, not Lewy bodies, cause neurodegeneration in dementia with Lewy bodies. J Neurosci.

[193] Andersen KB, Hansen AK, Damholdt MF (2021). Reduced synaptic density in patients with Lewy body dementia: an [11C]UCB-J PET imaging study. Mov Disord.

[194] Desplats P, Lee HJ, Bae EJ (2009). Inclusion formation and neuronal cell death through neuron-to-neuron transmission of alpha-synuclein. Proc Natl Acad Sci U S A.

[195] Crews L, Spencer B, Desplats P (2010). Selective molecular alterations in the autophagy pathway in patients with Lewy body disease and in models of alpha-synucleinopathy. PLoS One.

[196] Bano D, Zanetti F, Mende Y, Nicotera P (2011). Neurodegenerative processes in Huntington's disease. Cell Death Dis.

[197] Nopoulos PC (2016). Huntington disease: a single-gene degenerative disorder of the striatum. Dialogues Clin Neurosci.

[198] Rüb U, Seidel K, Heinsen H, Vonsattel JP, den Dunnen WF, Korf HW (2016). Huntington's disease (HD): the neuropathology of a multisystem neurodegenerative disorder of the human brain. Brain Pathol.

[199] Li S, Li XJ (2006). Multiple pathways contribute to the pathogenesis of Huntington disease. Mol Neurodegener.

[200] Blumenstock S, Dudanova I (2020). Cortical and striatal circuits in Huntington’s disease. Front Neurosci.

[201] Zhou H, Cao F, Wang Z (2003). Huntingtin forms toxic NH2-terminal fragment complexes that are promoted by the age-dependent decrease in proteasome activity. J Cell Biol.

[202] Marsh JL, Walker H, Theisen H, Zhu YZ, Fielder T, Purcell J, Thompson LM (2000). Expanded polyglutamine peptides alone are intrinsically cytotoxic and cause neurodegeneration in Drosophila. Hum Mol Genet.

[203] Xiao G, Fan Q, Wang X, Zhou B (2013). Huntington disease arises from a combinatory toxicity of polyglutamine and copper binding. Proc Natl Acad Sci U S A.

[204] Duennwald ML, Lindquist S (2008). Impaired ERAD and ER stress are early and specific events in polyglutamine toxicity. Genes Dev.

[205] Li SH, Lam S, Cheng AL, Li XJ (2000). Intranuclear huntingtin increases the expression of caspase-1 and induces apoptosis. Hum Mol Genet.

[206] Ochaba J, Lukacsovich T, Csikos G (2014). Potential function for the Huntingtin protein as a scaffold for selective autophagy. Proc Natl Acad Sci U S A.

[207] Shibata M, Lu T, Furuya T (2006). Regulation of intracellular accumulation of mutant Huntingtin by Beclin 1. J Biol Chem.

[208] Štětkářová I, Ehler E (2021). Diagnostics of amyotrophic lateral sclerosis: up to date. Diagnostics (Basel).

[209] Wijesekera LC, Leigh PN (2009). Amyotrophic lateral sclerosis. Orphanet J Rare Dis.

[210] Parakh S, Atkin JD (2016). Protein folding alterations in amyotrophic lateral sclerosis. Brain Res.

[211] Fecto F, Deng HX, Siddique T (2010). Discovering the connection between familial and sporadic amyotrophic lateral sclerosis: pathology trumps genetics. Future Neurol.

[212] Blokhuis AM, Groen EJ, Koppers M, van den Berg LH, Pasterkamp RJ (2013). Protein aggregation in amyotrophic lateral sclerosis. Acta Neuropathol.

[213] Paré B, Lehmann M, Beaudin M (2018). Misfolded SOD1 pathology in sporadic amyotrophic lateral sclerosis. Sci Rep.

[214] Cicardi ME, Marrone L, Azzouz M, Trotti D (2021). Proteostatic imbalance and protein spreading in amyotrophic lateral sclerosis. EMBO J.

[215] Grassano M, Calvo A, Moglia C (2022). Systematic evaluation of genetic mutations in ALS: a population-based study. J Neurol Neurosurg Psychiatry.

[216] Maor-Nof M, Shipony Z, Lopez-Gonzalez R (2021). p53 is a central regulator driving neurodegeneration caused by C9orf72 poly(PR). Cell.

[217] Pasinelli P, Houseweart MK, Brown RH Jr, Cleveland DW (2000). Caspase-1 and -3 are sequentially activated in motor neuron death in Cu,Zn superoxide dismutase-mediated familial amyotrophic lateral sclerosis. Proc Natl Acad Sci U S A.

[218] Ditsworth D, Maldonado M, McAlonis-Downes M (2017). Mutant TDP-43 within motor neurons drives disease onset but not progression in amyotrophic lateral sclerosis. Acta Neuropathol.

[219] Medchalmi S, Tare P, Sayyad Z, Swarup G (2021). A glaucoma- and ALS-associated mutant of OPTN induces neuronal cell death dependent on Tbk1 activity, autophagy and ER stress. FEBS J.

[220] Moore AS, Holzbaur EL (2016). Dynamic recruitment and activation of ALS-associated TBK1 with its target optineurin are required for efficient mitophagy. Proc Natl Acad Sci U S A.

[221] Filippi M, Bar-Or A, Piehl F, Preziosa P, Solari A, Vukusic S, Rocca MA (2018). Multiple sclerosis. Nat Rev Dis Primers.

[222] Vercellino M, Masera S, Lorenzatti M (2009). Demyelination, inflammation, and neurodegeneration in multiple sclerosis deep gray matter. J Neuropathol Exp Neurol.

[223] Anderson JM, Hampton DW, Patani R (2008). Abnormally phosphorylated tau is associated with neuronal and axonal loss in experimental autoimmune encephalomyelitis and multiple sclerosis. Brain.

[224] Gourraud PA, Harbo HF, Hauser SL, Baranzini SE (2012). The genetics of multiple sclerosis: an up-to-date review. Immunol Rev.

[225] Ofengeim D, Ito Y, Najafov A (2015). Activation of necroptosis in multiple sclerosis. Cell Rep.

[226] Feng X, Hou H, Zou Y, Guo L (2017). Defective autophagy is associated with neuronal injury in a mouse model of multiple sclerosis. Bosn J Basic Med Sci.

[227] Picon C, Jayaraman A, James R (2021). Neuron-specific activation of necroptosis signaling in multiple sclerosis cortical grey matter. Acta Neuropathol.

[228] Haile Y, Deng X, Ortiz-Sandoval C (2017). Rab32 connects ER stress to mitochondrial defects in multiple sclerosis. J Neuroinflammation.

[229] Patel H, Hodges AK, Curtis C, Lee SH, Troakes C, Dobson RJ, Newhouse SJ (2019). Transcriptomic analysis of probable asymptomatic and symptomatic Alzheimer brains. Brain Behav Immun.

[230] Bellia F, Vecchio G, Rizzarelli E (2014). Carnosinases, their substrates and diseases. Molecules.

[231] Caruso G, Caraci F, Jolivet RB (2019). Pivotal role of carnosine in the modulation of brain cells activity: multimodal mechanism of action and therapeutic potential in neurodegenerative disorders. Prog Neurobiol.

[232] Solana-Manrique C, Sanz FJ, Martínez-Carrión G, Paricio N (2022). Antioxidant and neuroprotective effects of carnosine: therapeutic implications in neurodegenerative diseases. Antioxidants (Basel).

[233] Distefano A, Caruso G, Oliveri V (2022). Neuroprotective effect of carnosine is mediated by insulin-degrading enzyme. ACS Chem Neurosci.

[234] Kuhn S, Gritti L, Crooks D, Dombrowski Y (2019). Oligodendrocytes in development, myelin generation and beyond. Cells.

[235] Gao Y, Liu J, Wang J, Liu Y, Zeng LH, Ge W, Ma C (2022). Proteomic analysis of human hippocampal subfields provides new insights into the pathogenesis of Alzheimer's disease and the role of glial cells. Brain Pathol.

[236] Lau SF, Cao H, Fu AK, Ip NY (2020). Single-nucleus transcriptome analysis reveals dysregulation of angiogenic endothelial cells and neuroprotective glia in Alzheimer's disease. Proc Natl Acad Sci U S A.

[237] Ishimoto T, Ninomiya K, Inoue R, Koike M, Uchiyama Y, Mori H (2017). Mice lacking BCAS1, a novel myelin-associated protein, display hypomyelination, schizophrenia-like abnormal behaviors, and upregulation of inflammatory genes in the brain. Glia.

[238] Desai MK, Sudol KL, Janelsins MC, Mastrangelo MA, Frazer ME, Bowers WJ (2009). Triple-transgenic Alzheimer's disease mice exhibit region-specific abnormalities in brain myelination patterns prior to appearance of amyloid and tau pathology. Glia.

[239] Zhang X, Wang R, Hu D (2020). Oligodendroglial glycolytic stress triggers inflammasome activation and neuropathology in Alzheimer's disease. Sci Adv.

[240] Olajide OJ, Suvanto ME, Chapman CA (2021). Molecular mechanisms of neurodegeneration in the entorhinal cortex that underlie its selective vulnerability during the pathogenesis of Alzheimer's disease. Biol Open.

[241] Rangaraju S, Dammer EB, Raza SA (2018). Identification and therapeutic modulation of a pro-inflammatory subset of disease-associated-microglia in Alzheimer's disease. Mol Neurodegener.

[242] Tesseur I, Zou K, Esposito L (2006). Deficiency in neuronal TGF-beta signaling promotes neurodegeneration and Alzheimer's pathology. J Clin Invest.

[243] Yan X, Xiong X, Chen YG (2018). Feedback regulation of TGF-β signaling. Acta Biochim Biophys Sin (Shanghai).

[244] Esteve P, Rueda-Carrasco J, Inés Mateo M (2019). Elevated levels of secreted-frizzled-related-protein 1 contribute to Alzheimer's disease pathogenesis. Nat Neurosci.

[245] Petrache AL, Rajulawalla A, Shi A (2019). Aberrant excitatory-inhibitory synaptic mechanisms in entorhinal cortex microcircuits during the pathogenesis of Alzheimer’s disease. Cereb Cortex.

[246] Miao N, Bian S, Lee T (2018). Opposite roles of Wnt7a and Sfrp1 in modulating proper development of neural progenitors in the mouse cerebral cortex. Front Mol Neurosci.

[247] Xu G, Cui Y, Wang L (2015). Temporospatial expression of fibulin-1 after acute spinal cord injury in rats. J Spinal Cord Med.

[248] Kim H, Yoo J, Shin J (2017). Modelling APOE ɛ3/4 allele-associated sporadic Alzheimer's disease in an induced neuron. Brain.

[249] Ota M, Sato N, Kimura Y, Shigemoto Y, Kunugi H, Matsuda H (2019). Changes of myelin organization in patients with Alzheimer’s disease shown by q-space myelin map imaging. Dement Geriatr Cogn Dis Extra.

[250] Gómez-Isla T, Price JL, McKeel DW Jr, Morris JC, Growdon JH, Hyman BT (1996). Profound loss of layer II entorhinal cortex neurons occurs in very mild Alzheimer's disease. J Neurosci.

[251] Price JL, Ko AI, Wade MJ, Tsou SK, McKeel DW, Morris JC (2001). Neuron number in the entorhinal cortex and CA1 in preclinical Alzheimer disease. Arch Neurol.

[252] Hernández-Ortega K, Arias C (2012). ERK activation and expression of neuronal cell cycle markers in the hippocampus after entorhinal cortex lesion. J Neurosci Res.

[253] Hernández-Ortega K, Ferrera P, Arias C (2007). Sequential expression of cell-cycle regulators and Alzheimer's disease-related proteins in entorhinal cortex after hippocampal excitotoxic damage. J Neurosci Res.

[254] Domínguez-Álvaro M, Montero-Crespo M, Blazquez-Llorca L, Plaza-Alonso S, Cano-Astorga N, DeFelipe J, Alonso-Nanclares L (2021). 3D analysis of the synaptic organization in the entorhinal cortex in Alzheimer’s disease. eNeuro.

[255] Ramos-Miguel A, Sawada K, Jones AA (2017). Presynaptic proteins complexin-I and complexin-II differentially influence cognitive function in early and late stages of Alzheimer's disease. Acta Neuropathol.

[256] Kumari E, Xu A, Chen R, Yan Y, Yang Z, Zhang T (2023). FSTL1-knockdown improves neural oscillation via decreasing neuronal-inflammation regulating apoptosis in Aβ(1-42) induced AD model mice. Exp Neurol.

[257] Podvin S, Miller MC, Rossi R (2016). The orphan C2orf40 gene is a neuroimmune factor in Alzheimer’s disease. JSM Alzheimers Dis Relat Dement.

[258] Hoe HS, Cooper MJ, Burns MP (2007). The metalloprotease inhibitor TIMP-3 regulates amyloid precursor protein and apolipoprotein E receptor proteolysis. J Neurosci.

[259] Wang BJ, Her GM, Hu MK (2017). ErbB2 regulates autophagic flux to modulate the proteostasis of APP-CTFs in Alzheimer's disease. Proc Natl Acad Sci U S A.

[260] Janelsins MC, Mastrangelo MA, Oddo S, LaFerla FM, Federoff HJ, Bowers WJ (2005). Early correlation of microglial activation with enhanced tumor necrosis factor-alpha and monocyte chemoattractant protein-1 expression specifically within the entorhinal cortex of triple transgenic Alzheimer's disease mice. J Neuroinflammation.

[261] Wang Y, Santa-Cruz K, DeCarli C, Johnson JA (2000). NAD(P)H:quinone oxidoreductase activity is increased in hippocampal pyramidal neurons of patients with Alzheimer’s disease. Neurobiol Aging.

[262] Rashid MH, Babu D, Siraki AG (2021). Interactions of the antioxidant enzymes NAD(P)H: Quinone oxidoreductase 1 (NQO1) and NRH: Quinone oxidoreductase 2 (NQO2) with pharmacological agents, endogenous biochemicals and environmental contaminants. Chem Biol Interact.

[263] Berchtold NC, Coleman PD, Cribbs DH, Rogers J, Gillen DL, Cotman CW (2013). Synaptic genes are extensively downregulated across multiple brain regions in normal human aging and Alzheimer's disease. Neurobiol Aging.

[264] Akanuma T, Koshida S, Kawamura A, Kishimoto Y, Takada S (2007). Paf1 complex homologues are required for Notch-regulated transcription during somite segmentation. EMBO Rep.

[265] Park J, Park S, Lee JS (2023). Role of the Paf1 complex in the maintenance of stem cell pluripotency and development. FEBS J.

[266] Van Oss SB, Shirra MK, Bataille AR (2016). The histone modification domain of Paf1 complex subunit Rtf1 directly stimulates H2B ubiquitylation through an interaction with Rad6. Mol Cell.

[267] Jarome TJ, Perez GA, Webb WM (2021). Ubiquitination of histone H2B by proteasome subunit RPT6 controls histone methylation chromatin dynamics during memory formation. Biol Psychiatry.

[268] Tu M, Zhu P, Hu S (2017). Notch1 signaling activation contributes to adult hippocampal neurogenesis following traumatic brain injury. Med Sci Monit.

[269] Grill B, Chen L, Tulgren ED (2012). RAE-1, a novel PHR binding protein, is required for axon termination and synapse formation in Caenorhabditis elegans. J Neurosci.

[270] Hanley JG, Khatri L, Hanson PI, Ziff EB (2002). NSF ATPase and α-/β-SNAPs disassemble the AMPA receptor-PICK1 complex. Neuron.

[271] Tanaka H, Sakaguchi D, Hirano T (2019). Amyloid-β oligomers suppress subunit-specific glutamate receptor increase during LTP. Alzheimers Dement (N Y).

[272] Qian X, Li X, Cai Q (2017). Phosphoglycerate kinase 1 phosphorylates Beclin1 to induce autophagy. Mol Cell.

[273] Liu H, Wang X, Shen P, Ni Y, Han X (2022). The basic functions of phosphoglycerate kinase 1 and its roles in cancer and other diseases. Eur J Pharmacol.

[274] Qiang SJ, Shi YQ, Wu TY (2022). The discovery of novel PGK1 activators as apoptotic inhibiting and neuroprotective agents. Front Pharmacol.

[275] Zhang X, Alshakhshir N, Zhao L (2021). Glycolytic metabolism, brain resilience, and Alzheimer’s disease. Front Neurosci.

[276] Yuan M, Wang Y, Huang Z (2022). Impaired autophagy in amyloid-beta pathology: a traditional review of recent Alzheimer's research. J Biomed Res.

[277] Carroll J, He J, Ding S, Fearnley IM, Walker JE (2021). TMEM70 and TMEM242 help to assemble the rotor ring of human ATP synthase and interact with assembly factors for complex I. Proc Natl Acad Sci U S A.

[278] Schägger H, Ohm TG (1995). Human diseases with defects in oxidative phosphorylation. 2. F1F0 ATP-synthase defects in Alzheimer disease revealed by blue native polyacrylamide gel electrophoresis. Eur J Biochem.

[279] Jęśko H, Wieczorek I, Wencel PL, Gąssowska-Dobrowolska M, Lukiw WJ, Strosznajder RP (2021). Age-related transcriptional deregulation of genes coding synaptic proteins in Alzheimer’s disease murine model: potential neuroprotective effect of fingolimod. Front Mol Neurosci.

[280] Wan J, Steffen J, Yourshaw M (2016). Loss of function of SLC25A46 causes lethal congenital pontocerebellar hypoplasia. Brain.

[281] Cai Q, Tammineni P (2016). Alterations in mitochondrial quality control in Alzheimer’s disease. Front Cell Neurosci.

[282] Manczak M, Calkins MJ, Reddy PH (2011). Impaired mitochondrial dynamics and abnormal interaction of amyloid beta with mitochondrial protein Drp1 in neurons from patients with Alzheimer's disease: implications for neuronal damage. Hum Mol Genet.

[283] Manczak M, Reddy PH (2012). Abnormal interaction between the mitochondrial fission protein Drp1 and hyperphosphorylated tau in Alzheimer's disease neurons: implications for mitochondrial dysfunction and neuronal damage. Hum Mol Genet.

[284] Eftekharzadeh B, Daigle JG, Kapinos LE (2018). Tau protein disrupts nucleocytoplasmic transport in Alzheimer’s disease. Neuron.

[285] Pritchard CE, Fornerod M, Kasper LH, van Deursen JM (1999). RAE1 is a shuttling mRNA export factor that binds to a GLEBS-like NUP98 motif at the nuclear pore complex through multiple domains. J Cell Biol.

[286] Ricci SB, Cerchiari U (2010). Spontaneous regression of malignant tumors: importance of the immune system and other factors (Review). Oncol Lett.

[287] Canete A, Gerrard M, Rubie H (2009). Poor survival for infants with MYCN-amplified metastatic neuroblastoma despite intensified treatment: the International Society of Paediatric Oncology European Neuroblastoma Experience. J Clin Oncol.

[288] Iehara T, Hiyama E, Tajiri T (2012). Is the prognosis of stage 4s neuroblastoma in patients 12 months of age and older really excellent?. Eur J Cancer.

[289] Kocak H, Ackermann S, Hero B (2013). Hox-C9 activates the intrinsic pathway of apoptosis and is associated with spontaneous regression in neuroblastoma. Cell Death Dis.

[290] Asagoshi K, Tano K, Chastain PD 2nd (2010). FEN1 functions in long patch base excision repair under conditions of oxidative stress in vertebrate cells. Mol Cancer Res.

[291] Balakrishnan L, Bambara RA (2013). Flap endonuclease 1. Annu Rev Biochem.

[292] Wu X, Wilson TE, Lieber MR (1999). A role for FEN-1 in nonhomologous DNA end joining: the order of strand annealing and nucleolytic processing events. Proc Natl Acad Sci U S A.

[293] Kikuchi K, Taniguchi Y, Hatanaka A (2005). Fen-1 facilitates homologous recombination by removing divergent sequences at DNA break ends. Mol Cell Biol.

[294] Huang M, Zeki J, Sumarsono N (2020). Epigenetic targeting of TERT-associated gene expression signature in human neuroblastoma with TERT overexpression. Cancer Res.

[295] Oza J, Ganguly B, Kulkarni A, Ginjala V, Yao M, Ganesan S (2016). A novel role of chromodomain protein CBX8 in DNA damage response. J Biol Chem.

[296] Chiruvella KK, Liang Z, Wilson TE (2013). Repair of double-strand breaks by end joining. Cold Spring Harb Perspect Biol.

[297] Frit P, Barboule N, Yuan Y, Gomez D, Calsou P (2014). Alternative end-joining pathway(s): bricolage at DNA breaks. DNA Repair (Amst).

[298] Aygun N (2017). Acquired Chromosomal Abnormalities and Their Potential Formation Mechanisms in Solid Tumours. Chromosomal abnormalities - a hallmark manifestation of genomic instability.

[299] Caracciolo D, Riillo C, Di Martino MT, Tagliaferri P, Tassone P (2021). Alternative non-homologous end-joining: error-prone DNA repair as cancer’s Achilles’ heel. Cancers (Basel).

[300] Sharma S, Javadekar SM, Pandey M, Srivastava M, Kumari R, Raghavan SC (2015). Homology and enzymatic requirements of microhomology-dependent alternative end joining. Cell Death Dis.

[301] Newman EA, Lu F, Bashllari D, Wang L, Opipari AW, Castle VP (2015). Alternative NHEJ pathway components are therapeutic targets in high-risk neuroblastoma. Mol Cancer Res.

[302] Keane S, de Weerd HA, Ejeskär K (2022). DLG2 impairs dsDNA break repair and maintains genome integrity in neuroblastoma. DNA Repair (Amst).

[303] Chiu CC, Weng YH, Yeh TH (2023). Deficiency of RAB39B activates ER stress-induced pro-apoptotic pathway and causes mitochondrial dysfunction and oxidative stress in dopaminergic neurons by impairing autophagy and upregulating α-synuclein. Mol Neurobiol.

[304] Pilgrim A, Cuya S, Chen D, Schnepp R (2019). Abstract 3657: defining the role of the RNA-binding protein MSI2 in neuroblastoma. Cancer Res.

[305] Fox M, Mott HR, Owen D (2020). Class IA PI3K regulatory subunits: p110-independent roles and structures. Biochem Soc Trans.

[306] Chagpar RB, Links PH, Pastor MC, Furber LA, Hawrysh AD, Chamberlain MD, Anderson DH (2010). Direct positive regulation of PTEN by the p85 subunit of phosphatidylinositol 3-kinase. Proc Natl Acad Sci U S A.

[307] Qiao J, Kang J, Cree J, Evers BM, Chung DH (2005). Gastrin-releasing peptide-induced down-regulation of tumor suppressor protein PTEN (phosphatase and tensin homolog deleted on chromosome ten) in neuroblastomas. Ann Surg.

[308] Zheng Y, Zhang L, Lu Q, Wang X, Yu F, Wang X, Lu Q (2009). NGF-induced Tyro3 and Axl function as survival factors for differentiating PC12 cells. Biochem Biophys Res Commun.

[309] Pajtler KW, Rebmann V, Lindemann M (2013). Expression of NTRK1/TrkA affects immunogenicity of neuroblastoma cells. Int J Cancer.

[310] Pajtler KW, Mahlow E, Odersky A (2014). Neuroblastoma in dialog with its stroma: NTRK1 is a regulator of cellular cross-talk with Schwann cells. Oncotarget.

[311] Jensen LM, Zhang Y, Shooter EM (1992). Steady-state polypeptide modulations associated with nerve growth factor (NGF)-induced terminal differentiation and NGF deprivation-induced apoptosis in human neuroblastoma cells. J Biol Chem.

[312] Zhu Y, Li Y, Haraguchi S (2013). Dependence receptor UNC5D mediates nerve growth factor depletion-induced neuroblastoma regression. J Clin Invest.

[313] Choo Z, Koh RY, Wallis K (2016). XAF1 promotes neuroblastoma tumor suppression and is required for KIF1Bβ-mediated apoptosis. Oncotarget.

[314] Fischer M, Oberthuer A, Brors B (2006). Differential expression of neuronal genes defines subtypes of disseminated neuroblastoma with favorable and unfavorable outcome. Clin Cancer Res.

[315] Naef R, Suter U (1998). Many facets of the peripheral myelin protein PMP22 in myelination and disease. Microsc Res Tech.

[316] Gershon TR, Shiraz A, Qin LX, Gerald WL, Kenney AM, Cheung NK (2009). Enteric neural crest differentiation in ganglioneuromas implicates Hedgehog signaling in peripheral neuroblastic tumor pathogenesis. PLoS One.

[317] Malin D, Sonnenberg-Riethmacher E, Guseva D, Wagener R, Aszódi A, Irintchev A, Riethmacher D (2009). The extracellular-matrix protein matrilin 2 participates in peripheral nerve regeneration. J Cell Sci.

[318] Ouyang M, Shen X (2006). Critical role of ASK1 in the 6-hydroxydopamine-induced apoptosis in human neuroblastoma SH-SY5Y cells. J Neurochem.

[319] Zhong X, Liu Y, Liu H, Zhang Y, Wang L, Zhang H (2018). Identification of potential prognostic genes for neuroblastoma. Front Genet.

[320] Oberthuer A, Hero B, Spitz R, Berthold F, Fischer M (2004). The tumor-associated antigen PRAME is universally expressed in high-stage neuroblastoma and associated with poor outcome. Clin Cancer Res.

[321] Fransson S, Kogner P, Martinsson T, Ejeskär K (2013). Aggressive neuroblastomas have high p110alpha but low p110delta and p55alpha/p50alpha protein levels compared to low stage neuroblastomas. J Mol Signal.

[322] Fransson S, Abel F, Kogner P, Martinsson T, Ejeskär K (2013). Stage-dependent expression of PI3K/Akt‑pathway genes in neuroblastoma. Int J Oncol.

[323] Liu Y, Wang D, Li Z (2022). Pan-cancer analysis on the role of PIK3R1 and PIK3R2 in human tumors. Sci Rep.

[324] Kuner P, Hertel C (1998). NGF induces apoptosis in a human neuroblastoma cell line expressing the neurotrophin receptor p75NTR. J Neurosci Res.

[325] Delloye-Bourgeois C, Bertin L, Thoinet K (2017). Microenvironment-driven shift of cohesion/detachment balance within tumors induces a switch toward metastasis in neuroblastoma. Cancer Cell.

[326] Sepporta MV, Praz V, Balmas Bourloud K (2022). TWIST1 expression is associated with high-risk neuroblastoma and promotes primary and metastatic tumor growth. Commun Biol.

[327] Zhang J, Han Y, Yan D, Zhou D, Yuan X, Zhao W, Zhang D (2022). Identification of key genes associated with risk and prognosis of neuroblastoma. J Mol Neurosci.

[328] Westerlund I, Shi Y, Holmberg J (2019). EPAS1/HIF2α correlates with features of low-risk neuroblastoma and with adrenal chromaffin cell differentiation during sympathoadrenal development. Biochem Biophys Res Commun.

[329] Chen QR, Bilke S, Wei JS (2006). Increased WSB1 copy number correlates with its over-expression which associates with increased survival in neuroblastoma. Genes Chromosomes Cancer.

